# Parameters and determinants of responses to selection in antibody libraries

**DOI:** 10.1371/journal.pcbi.1008751

**Published:** 2021-03-25

**Authors:** Steven Schulz, Sébastien Boyer, Matteo Smerlak, Simona Cocco, Rémi Monasson, Clément Nizak, Olivier Rivoire

**Affiliations:** 1 Center for Interdisciplinary Research in Biology (CIRB), Collège de France, CNRS UMR 7241, INSERM U1050, PSL University, Paris, France; 2 Département de biochimie, Faculté de Médecine, Université de Montréal, Montréal, Canada; 3 Max Planck Institute for Mathematics in the Sciences, Leipzig, Germany; 4 Laboratory of Physics of École Normale Supérieure, UMR 8023, CNRS & PSL University, Paris, France; 5 Laboratory of Biochemistry, CBI, UMR 8231, ESPCI Paris, PSL University, CNRS, Paris, France; Rutgers University, UNITED STATES

## Abstract

The sequences of antibodies from a given repertoire are highly diverse at few sites located on the surface of a genome-encoded larger scaffold. The scaffold is often considered to play a lesser role than highly diverse, non-genome-encoded sites in controlling binding affinity and specificity. To gauge the impact of the scaffold, we carried out quantitative phage display experiments where we compare the response to selection for binding to four different targets of three different antibody libraries based on distinct scaffolds but harboring the same diversity at randomized sites. We first show that the response to selection of an antibody library may be captured by two measurable parameters. Second, we provide evidence that one of these parameters is determined by the degree of affinity maturation of the scaffold, affinity maturation being the process by which antibodies accumulate somatic mutations to evolve towards higher affinities during the natural immune response. In all cases, we find that libraries of antibodies built around maturated scaffolds have a lower response to selection to other arbitrary targets than libraries built around germline-based scaffolds. We thus propose that germline-encoded scaffolds have a higher selective potential than maturated ones as a consequence of a selection for this potential over the long-term evolution of germline antibody genes. Our results are a first step towards quantifying the evolutionary potential of biomolecules.

## Author summary

Antibodies in the immune system consist of a genetically encoded scaffold that exposes a few highly diverse, non-genetically encoded sites. This focused diversity is sufficient to produce antibodies that bind to any target molecule. To understand the role of the scaffold, which acquires hypermutations during the immune response, over the selective response, we analyze quantitative in vitro experiments where large antibody populations based on different scaffolds are selected against different targets. We show that selective responses are described statistically by two parameters, one of which depends on prior evolution of the scaffold as part of a previous response. Our work provides methods to assay whether naïve antibody scaffolds are endowed with a distinctively high selective potential.

## Introduction

The idea that evolution by natural selection is not only leading to adaptations but to a propensity to adapt, or “evolvability”, has been repeatedly put forward [1–3]. As demonstrated by a number of mathematical models, evolvability can indeed emerge from evolutionary dynamics without any direct selection for it [4–7]. Yet, theoretical insights have not translated into experimental assays for measuring and controlling evolvability in actual biological systems. Biomolecules as RNAs and proteins are ideal model systems for developing such assays as they are amenable to controlled experimental evolution [8]. For proteins, in particular, several biophysical and structural features have been proposed to correlate with their evolvability, most notably their thermal stability [9, 10] and the modularity and polarity of their native fold [11]. A major limitation, however, is the absence of a measurable index of evolvability quantifying evolutionary responses to compare to biophysical or structural quantities.

Here, we introduce a quantitative approach to address this issue and present experimental results that point towards an evolutionary determinant of evolvability in the case of antibodies. Antibodies are particularly well suited to devise and test new approaches to measure and control evolvability, as diverse libraries of billions of different antibodies can be manipulated in vitro by well-established screening techniques [12]. The natural diversity of antibodies is remarkable. Their variable regions span a large phenotypic diversity, allowing specific binding to virtually any molecular target. At the sequence level, this diversity has different origins. First, the variable regions of naïve antibody genes are formed by combining two or three out of tens of genomic segments, with additional randomization at the junction between segments. Second, variable regions of antibodies undergo random somatic mutations along their sequence and selection for higher affinity through the fast evolutionary process of affinity maturation [13]. At the structural level, antibody variable regions consist of a framework displaying variable surface loops called complementary determining regions (CDRs), the most variable one, CDR3, being partially encoded by the randomized sites at junctions between segments [14]. The surface loops, which contain most but not all of the substitutions found in maturated antibodies, and especially the CDR3 loop, are thought to be the primary determinants of binding affinity and specificity [14]. However, the framework has been shown to play an essential role in several cases. In particular the large fraction of framework somatic mutations found in many broadly neutralizing antibodies to HIV have been reported to be required to confer neutralization towards a broad range of viral strains [15].

Antibody variable regions are thus subject to evolution by natural selection on two distinct time scales: their genome-encoded segments evolve on the time scale of many generations of their host, as all other genes, while naïve antibodies assembled from those genome-encoded segments additionally evolve on a much shorter time scale as part of the immune response in the process of affinity maturation. Importantly, affinity maturation-associated mutations are somatic and the sequences of maturated antibodies are not transmitted to subsequent generations. Germline antibody genomic segments, whose transmitted sequences are the starting point of affinity maturation, are thus well positioned to be particularly evolvable, as evolving to increase antibody affinity to antigens is part of their physiological role.

As a first step towards quantifying and controlling the evolvability of antibodies, we previously characterized the response to selection of antibody libraries built around different scaffolds [16]. We define scaffold as the genome-encoded sites of an antibody sequence. In a naïve antibody, the scaffold amino acids are identical to germline amino acids; in affinity maturated antibodies, some scaffold sites are somatically mutated. We took for these scaffolds the heavy chains (V_H_) of natural antibodies, including their framework regions and CDR1 and CDR2 loops, and built libraries by introducing all combinations of amino acids at four consecutive sites in their CDR3 loop. Using phage display [17], we selected sequences from these libraries for their ability to bind different molecular targets and analyzed the relative enrichment of different antibody sequences through successive cycles of selection and re-amplification by high-throughput sequencing [18]. Comparing experiments with libraries built on different scaffolds and selected against different targets led us to two conclusions. First, we quantified the variability of responses to selection of different sequences within a library and found this variability to differ widely across experiments involving different libraries and/or different targets. Second, we observed a hierarchy of enrichments between libraries, with multiple sequences from one particular library dominating selections involving a mixture of different libraries. These results raised two questions: (i) How to relate the hierarchies of enrichments between and within libraries? (ii) How to rationalize the differences between scaffolds that are all homologous?

Here, we address these two questions through the presentation of new data and new analyses. First, we propose to characterize the hierarchies within and between libraries with two parameters for which we provide interpretations from the three standpoints of physics, information theory and sequence content. One of these parameters, *σ*, reports the phenotypic variability within a library and thus quantifies the potential of a library to respond to selection. Second, we present new experimental results and re-analyze previous results to provide evidence that the degree of maturation of an antibody scaffold is a control parameter for its selective potential. Our approach thus provides a general and quantitative framework to study experimentally the selective potential of biomolecules. Our results are also, to our knowledge, the first to indicate that long-term evolution may have endowed germline antibodies with a special ability to respond to selection.

## Experimental design

In the absence of mutations, the outcome of an evolutionary process is determined by the properties of its initial population. Our initial populations are libraries made of sequences with a common part, which we call a scaffold, and 4 positions *x* = (*x*_1_, *x*_2_, *x*_3_, *x*_4_) that are randomized to all *N* = 20^4^ combinations, where 20 is the number of natural amino acids. We subject these populations to successive cycles of selection for binding against a target *T* and amplification. The critical property of a sequence *x* present in the initial population is its enrichment *s*(*x*), the factor by which it is enriched or depleted from one cycle to the next (see Box). The mapping *x* ↦ *s*_*L*,*T*_(*x*) from 4-position sequences *x* to enrichments generally depends both on the scaffold that defines the library *L* and on the target *T* that defines the selective pressure.

Experiments are designed for *s*(*x*) to reflect the binding affinity of an antibody with CDR3 sequence *x* to the chosen target *T* (S1 Text 1.1). In effect, however, selection does not depend exclusively on the CDR3 sequence *x* and the target *T* as phage-displayed antibodies may also be selected because they bind to something else than the target (the recipient or another phage) or because they bind to the target through their antibody scaffold. Such non-specific binding is generally negligible for the CDR3 sequences *x* of antibodies with top binding affinities to the target, but it dominates the selection of the majority of antibodies, which typically show no or weak CDR3 sequence-specific binding to the target. Following common practice in the field, we therefore perform three cycles of selection to enrich the population in strong binders. We are interested in properties of the scaffold that favor these large enrichment values, either relative to other sequences within the same library (same scaffold) or relative to sequences from different libraries (different scaffolds).

Our previous experiments involved 24 different libraries, each built on a different scaffold consisting of a natural V_H_ fragment [16]. These fragments originate from the germline or the B cells of organisms of various species. Scaffolds from the germline have not been subject to any affinity maturation, while scaffolds from B cells are taken from maturated antibodies which have evolved from naïve antibodies to bind strongly to antigens encountered by the organisms. We previously performed experiments where the initial population consisted either of a single library or a mixture of different libraries [16]. In particular, in two experiments using very different targets (a neutral polymer and a DNA loop) we co-selected all 24 libraries together. Strikingly, while only 2 of the 24 libraries were built on germline-based scaffolds, the final population of one experiment was dominated by antibodies built on one of the two germline-based scaffolds, and the second by the other one. This suggests that germline scaffolds may have an intrinsically higher selective potential.

To investigate this hypothesis, we performed the selection against 4 different targets of 3 libraries built on scaffolds with varying degrees of maturation. The 3 single-domain V_H_ libraries are based on V genes from the heavy chain of 3 human antibodies that have evolved to different degrees as part of the immune response to HIV (S1 Fig). They bear identically randomized CDR3 at 4 sites (upstream of a common human framework FWR4 region JH4 and no light chain). The Lim and Bnab scaffolds are derived from antibodies isolated from patients (6-187 and PGT128) [19, 20] and have respectively limited and broad spectrum of neutralization of HIV strains [15, 21]. Previous studies [15] concluded that the heavy chain V genes of these antibodies result from distinct affinity maturation trajectoires originating from a common germline origin (IGHV4-39) on which our Germ scaffold is based. Our Germ scaffold has thus not undergone any maturation. The Lim scaffold differs from Germ, from which it originates, by 14% of its amino acids. The Bnab scaffold also originates from Germ, to which it differs by 34% of its amino acids, and has evolved independently of Lim, to which it differs by 38%; the CDR2 of the Bnab scaffold also includes an insertion of 6 amino acids. The 3 single-domain V_H_ libraries, which are built around these V_H_ scaffolds by introducing all combinations of amino acids at 4 positions of their CDR3, were part of the 24 libraries used in our previous experiments [16]. Here, to systematically compare the selective potential of these libraries, we present experiments where they are selected against four different targets, two DNA targets (DNA hairpins with a common stem but different loops, denoted DNA1 and DNA2, S2 Fig) and two structurally related protein targets (the fluorescent proteins eGFP and mCherry, denoted prot1 and prot2), each unrelated to the HIV virus against which the Lim and Bnab scaffolds had been maturated.

## Results

**Fig 1:**
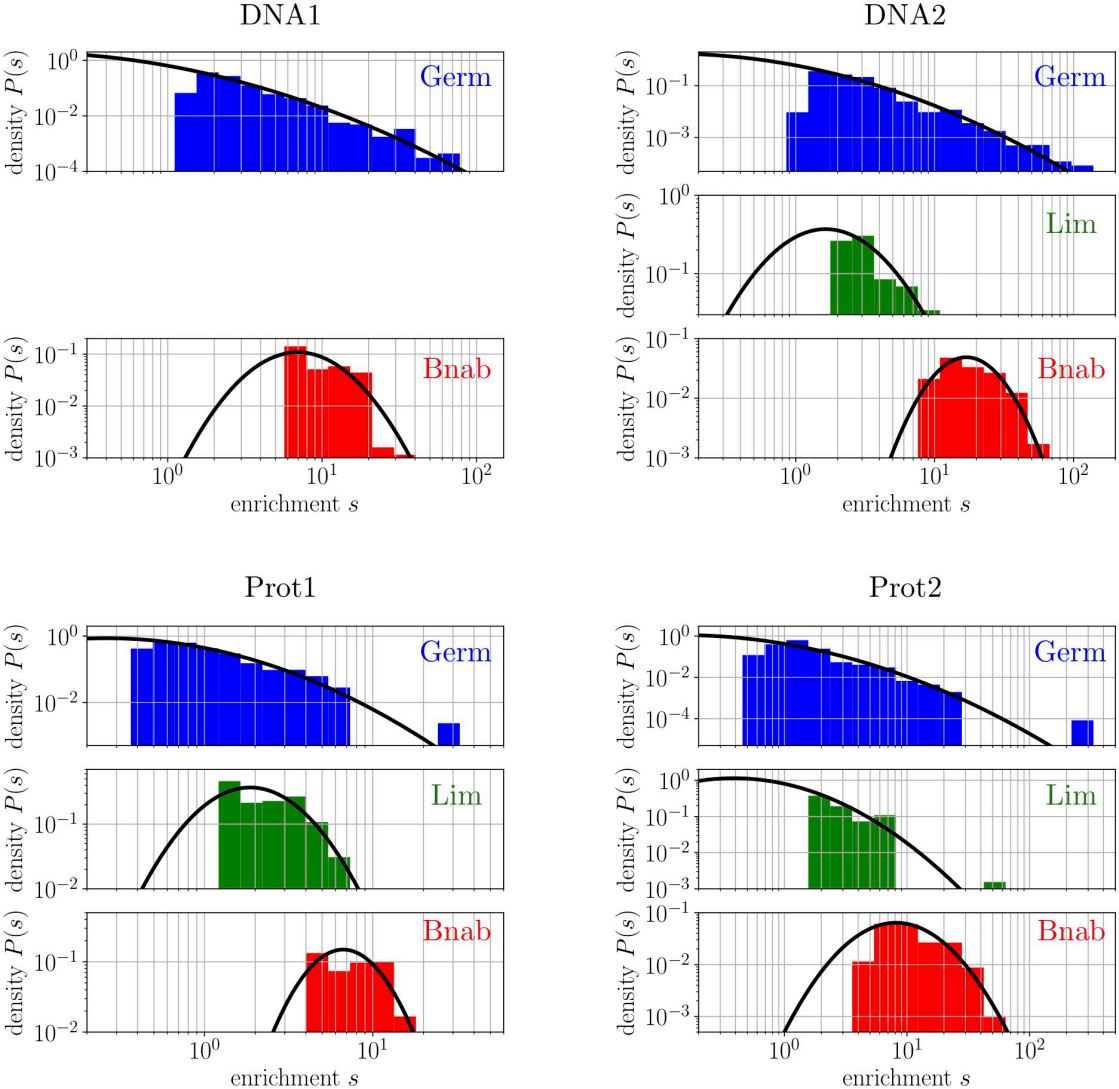
Fitting empirical distributions of top enrichments with log-normal distributions. The Germ, Lim and Bnab libraries were jointly selected against 4 targets. The top enrichments are fitted for each library independently to the tail of a log-normal distribution (black curve). The quality of the fits is validated by probability-probability and quantile-quantile plots (S16–S18 Figs). Data for the Lim library against the DNA1 target is not available as sequences from these libraries were too few at the rounds 2 and 3 of selection at which the enrichments are measured. The *σ* (but not the *μ*) of the log-normal was obtained for this case by selecting the library in isolation (S8 Fig). The value of *σ* quantifies the observation that top enrichments from the Germ library are spread over a larger range of values than top enrichments from the Bnab library, irrespectively of the target. The Lim library displays an intermediate behavior (S1 Table).

The distributions of top enrichments obtained from selecting jointly the three libraries against each of the four targets are shown in Fig 1. One result is immediately apparent: top enrichments from the Germ library are spread over a larger range of values than top enrichments from the other libraries, irrespectively of the target. This suggests that Germ libraries have a larger selective potential than their maturated counterparts. To justify the threshold above which enrichments are displayed in Fig 1, quantify the spread, and show that differences are also present between the Lim and Bnab libraries, we introduce and apply a simple model where the top enrichments are fitted to the tail of a lognormal distribution. We also report additional analyses to show how sequences with top enrichments differ from one experiment to the next.

### Parametrization

To quantitatively compare the outcome of different experiments with different libraries and targets, we introduce here two parameters, *σ* and *μ*, which respectively quantify intra and inter-library differences in enrichments. These parameters derive from a statistical approach that considers only the distribution *P*(*s*) of values that enrichments take across the different sequences of a library [22–24]. They correspond to the assumption that this distribution is log-normal,

$$\begin{array}{c} P\left(s\right)=\frac{1}{\sqrt{2\pi }\sigma s}\text{exp}\;\left(-\frac{{\left(\text{ln}\;s-\mu \right)}^{2}}{2\sigma^{2}}\right). \end{array}$$ (1)

The parameter *σ* captures intra-library differences in response to selection while the parameter *μ* provides the additional information required to describe inter-library differences.

The parametrization of the distributions of enrichments by log-normal distributions has several motivations. First, it empirically provides a good fit of the data, not only in our experiments as we show below, but in a number of previous studies of antibody-antigen interactions [25] and protein-DNA interactions [26], including studies that had access to the complete distribution *P*(*s*) [26]. Second, log-normal distributions are stable upon iteration of the selective process: if two successive selections are performed so that *s* = *s*_1_ *s*_2_ with *s*_1_ and *s*_2_ independently described by log-normal distributions, then *s* also follows a log-normal distribution; more generally, log-normal distributions are attractors of evolutionary dynamics [27]. Third, log-normal distributions are physically justified from the simplest model of interaction, an additive model where the interaction energy between sequence *x* = (*x*_1_, …, *x*_*ℓ*_) of length *ℓ* and its target takes the form $\beta \Delta G\left(x\right)=\sum_{i=1}^{\ell }h_{i}\left(x_{i}\right)$ with contributions *h*_*i*_(*x*_*i*_) from each position *i* and amino acid *x*_*i*_, and thus its enrichment is *s*(*x*) ≃ e^−*β*Δ*G*(*x*)^, where *T* is the temperature and *k*_*B*_ the Boltzmann constant (S1 Text 1.1). At thermal equilibrium and for sufficiently large *ℓ*, a log-normal distribution of the affinities is then expected with *μ* ∼ −*ℓ*〈*h*〉 and *σ* ∼ *ℓ*^1/2^(〈*h*^2^〉 − 〈*h*〉^2^)^1/2^, where 〈*h*〉 and 〈*h*^2^〉 − 〈*h*〉^2^ are respectively the mean and variance of the values of binding energies per position *h*_*i*_(*x*_*i*_). This additive model, which ignores epistasis between the sites *i* is not expected to be exact but can provide a first approximation of the data (S1 Text 3.3). The central limit theorem, on which the above argument is based, in fact remains valid in presence of weak epistasis. We also note that the model does not exclude epistasis between the sites *i* and the scaffold, which will be shown to be essential. The parameter *σ*, which quantifies the diversity of enrichment values within a library, also corresponds to a natural measure of diversity from the standpoint of information theory (S1 Text 1.3). These multiple empirical and theoretical justifications motivate a description of the distributions of enrichments from selections of antibody libraries by log-normal distributions. We show below that our data does not exclude descriptions by other distributions, from which the same main conclusions can be drawn.

### Inference of parameters

The enrichment *s*(*x*) of a sequence *x* is obtained from comparing the frequency of *x* in the population before and after a round of selection. As only the largest enrichments are expected to reflect specific binding to the target, we obtain the parameters *σ* and *μ* by fitting the values with truncated log-normal distributions, when *s*(*x*) exceeds a threshold s* (Fig 1A and Methods). The threshold s* is chosen so that larger thresholds s** > s* yield comparable values of *σ* and *μ*, with the exclusion of very large thresholds s** that leave too few data-points to make a sensible inference (S26, S27 and S28 Figs for an illustration with simulated data). A complication is that enrichments are defined only up to a multiplicative factor (see Box). While the parameter *σ* is independent of this multiplicative factor, comparing the parameters *μ* between libraries requires performing selections where different libraries are mixed in the initial population.

**Fig 2:**
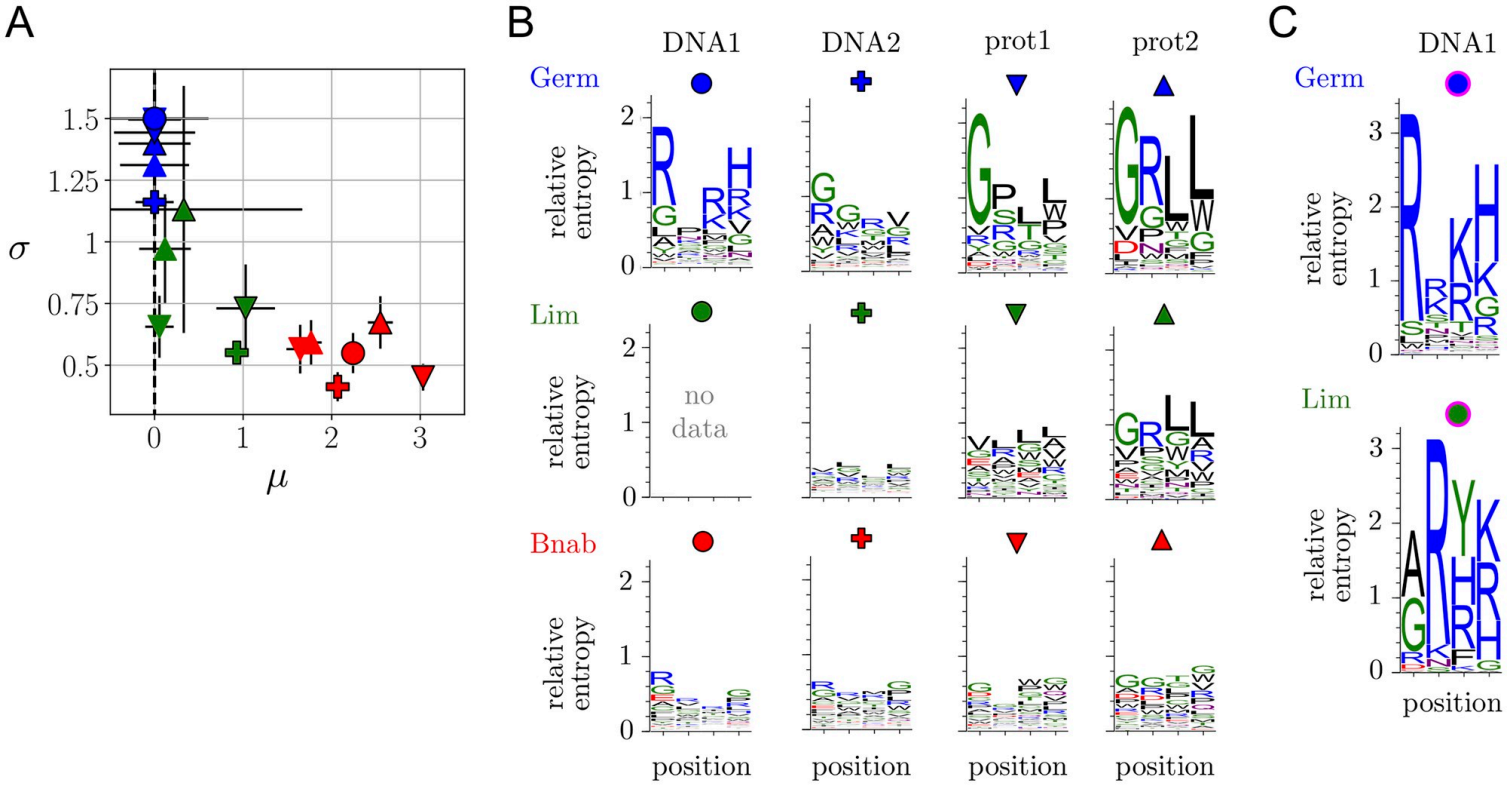
Comparing selections of libraries built on scaffolds with different degrees of maturation. **A**. Parameters (*μ*, *σ*) of the distributions of enrichments for our 3 libraries selected against 4 targets. The color of the symbols indicates the library (Germ, Lim or Bnab) and its shape the target (DNA1, DNA2, prot1 or prot2) with the conventions defined in B. Symbols with a black or no contour indicate results from replicate experiments where the 3 libraries are mixed in the initial population. *μ*_Germ,*T*_ is conventionally set to *μ*_Germ,*T*_ = 0 for all targets *T* (Methods). *μ* is generally more challenging to infer than *σ* and it shows here more variations across replicate experiments. **B**. Sequence logos for ${\tilde{s}}_{i}\left(a\right)$, which represent the contribution of the different amino acids to the enrichments (see Box), for the selections of the three libraries, Germ, Lim and Bnab against the two DNA targets (DNA1 and DNA2) and the two protein targets (prot1 and prot2). These results correspond to the experiments of Fig 1 where the 3 libraries are mixed in the initial population. The Lim library is outcompeted by the other two libraries when selected against the DNA1 target, which does not leave enough sequences to make a meaningful inference (see also S10 Fig for more details on the sequence logos for the Bnab library). **C**. Sequence logos for ${\tilde{s}}_{i}\left(a\right)$ for the Germ and Lim libraries selected in isolation against the DNA1 target. For the Lim library, this palliates the absence of data in B. For the Germ library, it shows that the same motif with *x*_1_ = *R*, *x*_3_ = *R* or *K* and *x*_4_ = *H* dominates whether the library is selected in a mixture as in B or on its own; the area under the logos is, however, different: it would be *σ*^2^/2 with infinite sampling, but major deviations are caused by limited sampling (S9 Fig).

The values of *σ* and *μ* that we infer for the 3 libraries Germ, Lim and Bnab when selected against each of the 4 targets DNA1, DNA2, prot1 and prot2 are presented in Fig 2A. We validated the quality of the fits by probability-probability and quantile-quantile plots (S16–S22 Figs), and by comparing experiments where a library is selected either alone or in mixture with the other two (S1 Table, S19 Fig). We verified that the results are unchanged whether enrichments are measured by comparing frequencies between the 2nd and 3rd cycles, or between the 3rd and 4th cycles (S1 Table and S20 and S21 Figs). Finally, we also performed selection experiments where we mixed a very small number of random and top enrichment sequences, which allows for a very precise estimation of the relative enrichments (S12, S13 and S25 Figs). These experiments verify that sequences identified to have top enrichments are significantly more enriched than random sequences when *σ* is large, as in the case of the Germ library, but not when *σ* is small, as in the case of the Bnab library. The Lim library shows an intermediate behavior consistent with its intermediate value of *σ*.

### Intra-library hierarchy

The hierarchy of enrichments within a library is quantified by the parameter *σ*: a small *σ* indicates that all sequences in the library are equally selected while a large *σ* indicates that the response to selection varies widely between sequences in the library. When comparing the *σ*_*L*,*T*_ inferred from the selections of the 3 libraries *L* against each of the 4 targets *T*, a remarkable pattern emerges: the more a scaffold is maturated, the smaller is *σ*, *σ*_Germ,*T*_ > *σ*_Lim,*T*_ ≥ *σ*_Bnab,*T*_ for all targets *T*, and even min_*T*_(*σ*_Germ,*T*_) > max_*T*_(*σ*_Lim,*T*_, *σ*_Bnab,*T*_) (Fig 2A). Statistically, if considering the inequalities to be strict, the experiments to be independent and any result to be *a priori* equally likely, the probability of this finding is only *p* = (3!)^−4^ ≃ 7.10^−4^.

Although selections of the Germ library are characterized by a similarly high value of *σ* for the 4 targets, the sequences that are selected against each target are different. This is illustrated through sequence logos (Fig 2B and 2C). These sequence logos do not fully capture the specificity against each target, as they ignore any epistasis between the sites, but observing that they are different is sufficient to conclude that selection is target-specific. The amino acids found to be enriched are consistent with the nature of the targets: selections against the DNA targets are dominated by positively charged amino acids (letters in blue) and selections against the two protein targets, which are closely structurally related, are dominated by similar amino acid motifs.

In contrast, sequences logos for the Bnab library show motifs that are less dependent on the target (Fig 2B and S10 Fig). This observation is rationalized by an experiment where only the amplification step is performed, in the absence of any selection for binding. Sequence-specific amplification biases are then revealed, with sequence motifs that are similar to those observed when selection for binding is present (S10 Fig). With protein targets at least, the motifs are nevertheless sufficiently different to infer that selection for binding to the target contributes significantly to the enrichments (see also S6 Fig). Target-specific selection for binding, which is dominating the top enrichments in the Germ library (S11 Fig), is thus of the same order of magnitude as amplification biases for the top enrichments in the Bnab library.

Remarkably, the Lim library behaves either like the Germ library or the BnAb library, depending on the target. In particular, a motif of positively charged amino acids emerges when selecting it against one of the two DNA targets (DNA1), but no clear motif emerges when selecting it against the other one (DNA2) (Fig 2B). Besides, when a clear motif emerges, it can be identical to the motif emerging from the Germ library as in case of a selection against the prot2 target (Fig 2B), or different, as in the case of a selection against the DNA1 target (but with a similar selection of positively charged amino acids) (Fig 2C).

### Inter-library hierarchy

The hierarchy of enrichments between libraries is quantified by the parameter *μ*. This parameter also shows a pattern that is independent of the target: *μ*_Germ,*T*_ ≃ *μ*_Lim,*T*_ < *μ*_Bnab,*T*_ and even max_*T*_ (*μ*_Germ,*T*_, *μ*_Lim,*T*_) < min_*T*_ (*μ*_Bnab,*T*_) (Fig 2A). Inferring *μ* is more challenging than inferring *σ* and the differences observed between the Germ and Lim libraries are most likely not significant, as apparent from the observed variations between replicate experiments. The *μ* of the Bnab library is, on the other hand, systematically larger. The difference is explained by an experiment where selection is performed in the absence of DNA or protein targets but in the presence of streptavidin-coated magnetic beads to which these targets are usually attached. This experiment reproduces the differences in *μ*_*L*,*T*_, which indicates a small but significant affinity of the Bnab scaffold for the magnetic beads, independent of the sequence *x* (S12 Fig). While the differences in *σ* appear to be independent of the target, the differences in *μ* are thus related to a common feature of the targets. Given these different origins, the correlation between *σ* and *μ* that we observe may be fortuitous.

### Implications for evolutionary dynamics

**Fig 3:**
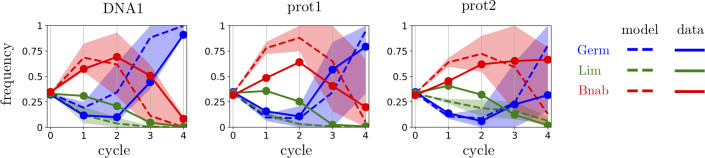
Dynamics of library frequencies. A mixture of the three libraries, Germ (blue), Lim (green) and Bnab (red) was subject to four successive cycles of selection and amplification against different targets. The full lines report the evolution of the relative frequencies of the three scaffolds. The dotted lines represent the estimated dynamics using the characterization of each library by a log-normal distribution with the parameters *σ*, *μ* estimated from the selection of the libraries against the same target (S1 Text 1.5). The shaded area correspond to one standard deviation in the estimation of the parameters *σ*, *μ*. The fit is only qualitative as we assume here that sequences are uniformly represented in each initial library, which is not the case in experiments. The trends, which are controlled by the two parameters *σ* and *μ*, are nevertheless well reproduced.

The different patterns of intra- and inter-library hierarchies lead to non-trivial evolutionary dynamics when selecting from an initial population that is composed of different libraries. In particular, a non-monotonic enrichment is expected when mixing two libraries characterized by (*μ*_1_, *σ*_1_) and (*μ*_2_, *σ*_2_) with *μ*_1_ > *μ*_2_ but *σ*_1_ < *σ*_2_: the library with largest *μ* dominates the first cycles while the one with largest *σ* dominates the later ones. This is indeed observed in experiments where different libraries are mixed in the initial population (Fig 3). The dynamics of the relative frequencies of different libraries are globally predicted by a calculation of library frequencies in the mix based on the parameters (*μ*_*L*_, *σ*_*L*_) inferred for each library *L* independently (S1 Text). We verify that the short-term dynamics are dominated by the library with largest *μ* while the long-term dynamics are dominated with the library with largest *σ*: which of the two parameters is most important thus depends on the considered time scale. The predictions reported in Fig 3 are based on two assumptions: (i) the distributions of enrichments in different libraries *L* are log-normal; (ii) the sequences in the initial population have equal frequencies. This second hypothesis is only an approximation for our experiments, which limits the validity of the predictions. Nevertheless, the results illustrate how parametrizing the response to selection of a library by the two parameters (*μ*,*σ*) is not only useful to characterize its intrinsic response but also to rationalize the evolutionary dynamics of mixtures of libraries.

### Additional data

**Fig 4:**
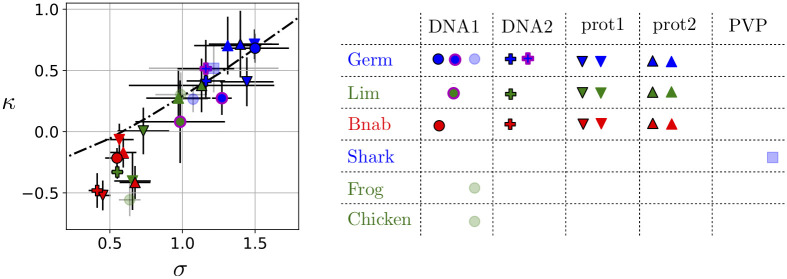
Shape parameter *κ* from fits of the enrichments to generalized Pareto distributions versus *σ* from fits to log-normal distributions. Results from different libraries selected against different targets are represented here with the same convention as in Fig 2: blue, green and red plain colors for the Germ, Lim and Bnab libraries, circle, cross, downward and upward triangles for the DNA1, DNA2, prot1 and prot2 targets. In addition, results from our previous work [16] are indicated in transparent blue if they involve a library built onto a germline scaffold and in transparent green if they involve a library built onto a maturated scaffold. The hierarchy indicated by *κ* is essentially the same as the hierarchy indicated by *σ*, consistent with the expected relationship between *κ* and *σ* (black dotted line, S14 Fig). By the two approaches, libraries built onto germline scaffolds are found to have a more diverse response to selection than libraries built onto maturated scaffolds irrespectively of the target (all values of *σ* and *κ* are given in S1 Table).

Beyond the 3 libraries analyzed so far, our conclusions are supported by re-analyzing our previous results [16]. These previous results involved a library based on another germline scaffold, 19 libraries built on other maturated scaffolds, and a completely different target, in addition to some of the same frameworks and targets presented in this work. Inferring *σ* from these data, we observe again that libraries built around germline scaffolds have larger *σ* than libraries built around maturated scaffolds (Fig 4 and S1 Table). These supplementary results corroborate the hypothesis that our measure of selective potential *σ* decreases in the course of affinity maturation.

## Discussion

The log-normal model provides a simple quantitative description of the data. As we discuss here, other statistical models may be considered that lead to similar conclusions. More elaborate analyses may also be performed, which go beyond the limitations of the present approach.

### An alternative to the log-normal model: Extreme value statistics

In our previous work [16], we fitted the tail of the distribution of enrichments with generalized Pareto distributions, a family of distributions with two parameters, a shape parameter *κ* and a scaling parameter *τ*. This was motivated by extreme value theory, which establishes that these parameters are sufficient to describe the tail of any distribution (S1 Text 1.2). For different libraries *L* and different targets *T*, we found that generalized Pareto distributions provide a good fit of the upper tail of *P*_*L*,*T*_(*s*), with, depending on the scaffold *L* and target *T*, either *κ* > 0 (heavy tail), *κ* < 0 (bounded tail) or *κ* = 0 (exponential tail). The origin of these different values of *κ* was, however, unclear.

Comparing probability-probability plots to assess the quality of the fits, our data appears equally well fitted by generalized Pareto distributions and log-normal distributions (S16–S22 Figs). This finding is at first sight puzzling as some of the fits with generalized Pareto distributions involve a non-zero shape parameter *κ* ≠ 0 but extreme value theory states that the tail of log-normal distributions is asymptotically described by a shape parameter *κ* = 0 for all values of *σ*, *μ* [28]. Extreme value theory is, however, only valid in the double asymptotic limit *N* → ∞ and s* → ∞, where *N* is the total number of samples and s* the threshold above which these samples are considered. With finite data, determining whether this asymptotic regime is reached is notoriously difficult when the underlying distribution is log-normal [29]. More precisely, *N* points randomly sampled from a log-normal distribution with parameter *σ* are known to display an apparent *κ*_*N*_ = *σ*/(2 ln *N*)^1/2^ which tends to zero only very slowly with increasing values of *N* [29]. In fact, this relationship itself requires *N* (or *σ*) to be sufficiently large and finite size effects can even produce an apparent *κ*_*N*_ < 0 (S14 Fig).

While casting doubt on the practical applicability of extreme value theory, these statistical effects do not call into question the main conclusion of our previous work [16]: different combinations of scaffolds *L* and targets *T* exhibit different within-library hierarchies, which are quantified by the different values of their (apparent) shape parameter *κ*. Fits with a log-normal distribution provide another parameter *σ* that report essentially the same differences (Fig 4). More importantly, we verify on our previous data, which partly involves different scaffolds and different targets, that libraries built on germline scaffolds have a higher *σ* than libraries built around maturated scaffolds (Fig 4 and S1 Table).

### Beyond the log-normal model

The log-normal model makes several assumptions that are only approximatively valid. First, it assumes that the measured enrichments faithfully reflect the probability for a sequence to be selected, which is exact only in the limit of infinitely large populations of selected and sequenced antibodies. Second, it assumes that this probability reflects equilibrium binding to the target through a simple non-epistatic relation between sequence and free energy of the type $x\mapsto s(x)=e^{-\sum_{i}h_{i}(x_{i})}$ with negligible contributions from other factors when considering the most enriched antibodies. Our populations of selected antibodies are large (∼ 10^12^) relative to the number of different sequences (∼ 10^5^) but the chance for a phage to be randomly selected is of order 10^−6^ and our sequencing depth is of order 10^5^, which induce stochastic effects. Following previous works [30, 31], we can account for the sampling noise due to sequencing by introducing a stochastic model for the observed numbers of sequences. We can also consider deviations from the simplest model *x* ↦ *s*(*x*) and account for other factors that may contribute to the enrichments, as non-specific binding. An analysis along those lines show how the selective potential of a library against a target can be more finely analyzed (S1 Text 1.5 and S23 Fig). This approach may be pushed further to account systematically for the different factors that contribute to the selection of antibodies in phage display experiments. Such an analysis may profitably replace the introduction of cut-offs to define top enrichments that reflect specific binding and allow for a joint treatment of all consecutive cycles of selection. It should also allow for better predictions of the fate of populations over multiple cycles of selection. The simplified analysis presented here is sufficient, however, for reporting differences in selective potentials between libraries (Fig 2) and for qualitatively reproducing the non-monotonic evolution of mixed libraries (Fig 4).

## Conclusion

In summary, we propose the hypothesis that naïve antibodies which are constructed from germline genes are endowed with a special evolutionary ability to generate selectable diversity, which they lose when undergoing affinity maturation. To study this hypothesis, we introduced an experimental and statistical approach that quantifies the selective potential of antibody scaffolds. In this approach, the response to selection of an antibody library against a given target is summarized by two parameters, *σ* and *μ*, which have different interpretations and implications. The parameter *σ* describes the variability of the responses between sequences in the library, while *μ* describes their common response. These two parameters may be viewed as quantifying the selective potential of a library over different time scales: when competing two libraries, the library with largest *μ* is initially more enriched but in the long-run sequences from the library with largest *σ* eventually dominate.

Applying this approach to data from our high-throughput selection experiments, we find results in favor of the hypothesis that germline-based antibody scaffolds have a higher potential to generate selectable diversity, corresponding to a higher *σ*. In particular, we analyzed new data centered onto 3 libraries, one built on a germline-based scaffold and two built on scaffolds derived from this germline-based scaffold with different degrees of maturation, which we selected against 4 different targets, all unrelated to the target against which the scaffold was originally maturated. We find that *σ* decreases with the degree of maturation. Our hypothesis is also corroborated by a re-analysis of our previous results, which involved a library built on another germline-based scaffold, 19 libraries built on other maturated scaffolds, and a completely different target [16]. Further experiments with additional scaffolds and targets are needed to assess the generality of these results and the limitations of our statistical description by means of only two parameters. The present work provides the motivation and the methodology to generate and analyze such data and study alternative scenarios. We also stress that our analysis is generally applicable to antibody library screening beyond testing our hypothesis, in particular to compare quantitatively in a single plot, as in Fig 2A, the outcome of many selection experiments involving several libraries and/or several targets.

Quantifying the selective potential of an antibody scaffold is a first step towards designing libraries with optimized selectable diversity. Once the property of a biomolecule is measurable, one can indeed resort to directed evolution to attempt to optimize it. Here, the starting point would be a population comprising different libraries with different scaffolds but identical random variations. We previously competed for binding to a target 24 such libraries [16], a number that could be increased. By alternating such selections with the introduction of new mutations in the scaffolds, one may be able to evolve scaffolds with increased *μ* and/or *σ*.

Which physical mechanisms may underly the differences in selective potential that we observe? A number of studies, ranging from structural biology to molecular dynamics simulations, have reported changes in antibody flexibility and target specificity over the course of affinity maturation [32–39]. The emerging picture is that naïve antibodies are flexible and polyspecific and become more rigid and more specific as they undergo affinity maturation. An increase of structural rigidity in the course of evolution is also found in proteins unrelated to antibodies [40]. Germline scaffolds may thus be more flexible than maturated scaffolds. If this scenario is correct, how this structural flexibility translates into evolutionary diversity once different complementary determining regions (CDRs) are grafted onto the scaffolds remains to be explained. Another biophysical property is also known to correlate with evolvability, thermal stability [9, 10]. The loss of selective potential that we observe may thus derive from a loss of thermal stability [41, 42]. Destabilization during affinity maturation might for instance arise from the interaction between the heavy and light chains of antibodies: germline heavy chains, which have to be robust to various light chain pairings, may be more stable than maturated heavy chain whose stability may depend on their associated light chain. Our results may thus be tied to the fact that we are studying heavy chains in isolation. Additional studies are needed to test this and other hypotheses and to identify the mechanisms behind the differences of selective potential that we measure.

Irrespective of mechanisms, our hypothesis and methodology may find applications beyond antibodies, to understand more generally what controls the selective potential of biomolecules. Beyond selection, a next step is to extend this work to quantify evolvability, i.e., the response to successive cycles of selection and mutations. Yet, being able to quantify the selective potential of a scaffold by an index that is systematically reduced in the course of evolution already raises an interesting challenge: can we increase this index to design libraries with better response to selection?

**BOX—Principles of antibody selection experiments**

We perform phage display experiments with different libraries of antibodies as input and different molecular targets (DNA hairpins or proteins) as selective pressures [17]. Our antibodies are single domains from the variable part of the heavy chain (V_H_) of natural antibodies. Antibodies in a library share a common scaffold of ≃ 100 amino acids and differ only at four consecutive sites of their third complementary determining region (CDR3), which is known to be important for binding affinity and specificity. A library comprises all combinations of amino acids at these four sites and therefore consists of a total of *N* = 20^4^ ≃ 10^5^ distinct sequences *x* = (*x*_1_, *x*_2_, *x*_3_, *x*_4_). Initial populations include a total of 10^11^ sequences, corresponding to ∼ 10^6^ copies of each of the distinct ∼ 10^5^ sequences when a single library is considered. Physically, these populations are made of phages, each presenting at its surface one antibody and containing the corresponding sequence.

**Fig 5:**
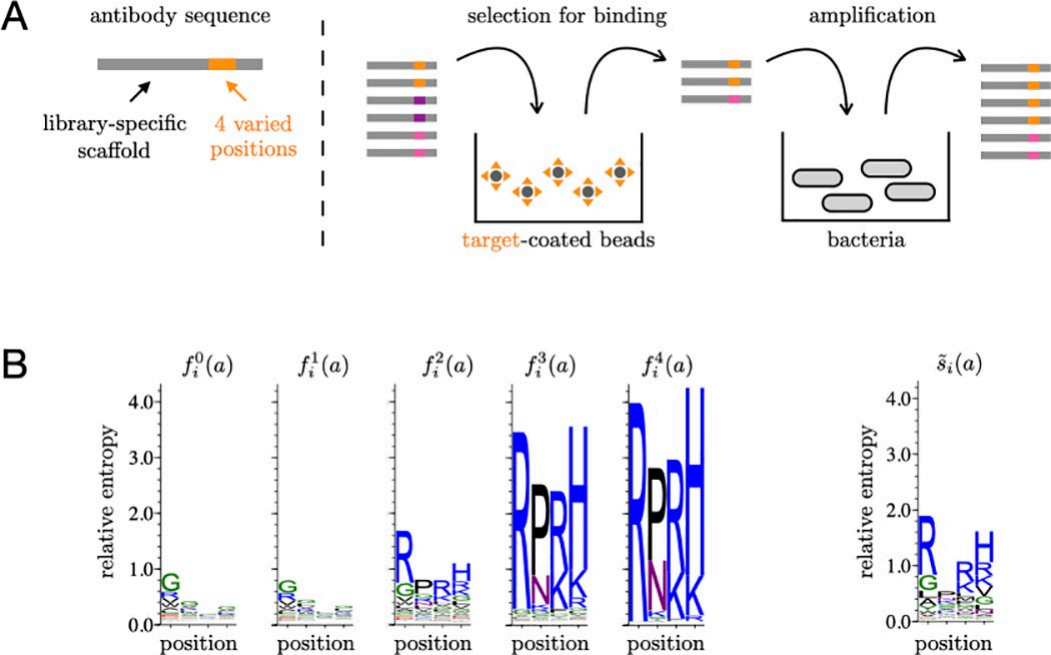
**A** Scheme of the experiment. **B** Sequence logos from selections of the Germ library against the DNA1 target.

An experiment consists in a succession of cycles, each composed of two steps (Fig 5A). In the first step, the phages are in solution with the targets, which are attached to magnetic beads and in excess relative to the phages to limit competitive binding (see S1 Text 1.1). The beads are retrieved with a magnet and washed to retain the bound antibodies. In the second step, the selected phages are put in presence of bacteria which they infect to make new phages, thus amplifying retained sequences. A population of ∼ 10^11^ phages is thus reconstituted. Both the selection for binding to the target and the amplification can possibly depend on the sequence of the antibody.

We define the enrichment *s*(*x*) of sequence *x* to be proportional to the probability for sequence *x* to pass one cycle. As the targets are in excess relative to the antibodies, enrichments are independent of the cycle *c* (see S1 Text 1.1). In the limit of infinite population sizes, *s*(*x*) is proportional to the ratio *f*^*c*^(*x*)/*f*^*c*−1^(*x*) of the frequencies *f*^*c*^(*x*) after any two successive cycles *c* − 1 and *c*. To estimate these enrichments, about 10^6^ sequences are sampled before and after a cycle and read by high-throughput sequencing. Given the counts *n*^*c*−1^(*x*) and *n*^*c*^(*x*) of sequence *x* before and after cycle *c*, we estimate the enrichment of *x* as

$$\begin{array}{c} s\left(x\right)=\alpha_{c}^{-1}\frac{n^{c}\left(x\right)}{n^{c-1}\left(x\right)} \end{array}$$ (2)

where *α*_*c*_ is an arbitrary multiplicative factor.

In practice, two types of noise must be taken into account when applying Eq (2): an experimental noise, which implies that antibodies have a finite probability to pass a round of selection independently of their sequence, and a sampling noise, which arises from the limited number of sequence reads. This sampling noise is negligible if *n*^*c*−1^(*x*) and *n*^*c*^(*x*) are sufficiently large. This is generally not the case for any sequence at the first cycle *c* = 1 where all *N* = 20^4^ sequences are present in too small numbers but becomes the case at the third cycle *c* = 3 for the 100 to 1000 sequences with largest enrichments. We therefore compute *s*(*x*) between the second and third cycles as $s\left(x\right)=\alpha_{3}^{-1}n^{3}\left(x\right)/n^{2}\left(x\right)$ by restricting to sequences *x* that satisfy *n*^2^(*x*) ≥ 10 and *n*^3^(*x*) ≥ 10. Additionally, as only the top enrichments reflect binding affinity, we retain only the sequences with *s*(*x*) > s* where s* is determined self-consistently (Methods and S3 Fig). Enrichments *s*(*x*) obtained by this procedure generally depend on the library (scaffold) *L* and the target *T* but are reproducible between independent experiments using the same library and the same target (S4 Fig).

To visualize the sequence dependence of enrichments, we use sequence logos [43]. In this representation, for each position *i* along the sequence, a bar of total height $\sum_{a}f_{i}^{c}\left(a\right)\;\text{ln}\;[20f_{i}^{c}\left(a\right)]$ is divided into letters, where each letter represents one of the 20 amino acids *a* with a size proportional to $f_{i}^{c}\left(a\right)$, the frequency of *a* at position *i* in the population after cycle *c*; for instance, $f_{2}^{c}\left(a\right)=\sum_{x_{1}=1}^{20}\sum_{x_{3}=1}^{20}\sum_{x_{4}=1}^{20}f^{c}\left(x_{1},a,x_{3},x_{4}\right)$; finally, the letters are colored by chemical properties: polar in green, neutral in purple, basic in blue, acidic in red and hydrophobic in black. It illustrates how some motifs are progressively enriched over successions of selective cycles. This representation is, however, dependent on the frequencies *f*^0^(*x*) of sequences in the initial population. To eliminate this dependency, we define an effective frequency ${\tilde{s}}_{i}\left(a\right)$ per position *i* and amino acid *a* as ${\tilde{s}}_{i}\left(a\right)=\sum_{x}s\left(x\right)\delta \left(x_{i},a\right)/\sum_{x}s\left(x\right)$, which would correspond to the frequency of *a* at position *i* after one round of selection if all sequences *x* were uniformly distributed in the initial population. It can also be represented by a sequence logo but depends only on *s*(*x*), as illustrated in Fig 5B by the Germ library selected against the DNA1 target (see S5–S7 Figs for other cases).

## Methods

Experimental methods are as in our previous work [16], except for target immobilization and sequencing data analysis as summarized in S1 Text. Here we present the methods of data analysis. Further theoretical background and additional statistical analyses are presented in S1 Text, and Python codes are provided in S1 Code.

### Noise cleaning with a threshold

Enrichments are computed from sequencing counts as indicated in Eq (2) in the Box. To account for sampling noise, only sequences whose count is ≥ 10 both at round *c* and *c* + 1 are considered. Moreover, we ignore enrichments *s*(*x*) below a threshold *s*^⋆^, which arise from unspecific binding. Unspecific binding modifies the expression for the enrichment of sequence *x* to include a sequence-independent unspecific binding energy Δ*G*_us_,

$$\begin{array}{c} s\left(x\right)=\frac{e^{-\beta \Delta G(x)}+e^{-\beta \Delta G_{\text{us}}}}{1+e^{-\beta \Delta G(x)}+e^{-\beta \Delta G_{\text{us}}}}. \end{array}$$ (3)

It sets a lower bound for the enrichment given by

$$\begin{array}{c} s_{\text{us}}=\frac{e^{-\beta \Delta G_{\text{us}}}}{1+e^{-\beta \Delta G_{\text{us}}}}=\frac{1}{1+e^{\beta \Delta G_{\text{us}}}}. \end{array}$$ (4)

The argument for log-normality of enrichment distributions applies only when the specific binding contribution Δ*G*(*x*) dominates the enrichment. We therefore eliminate the enrichments dominated by unspecific binding.

This is done by introducing a cut-off s*. The choice is made such that (i) the values of the inferred parameters $\hat{\sigma }$ and $\hat{\mu }$ are approximately constant for all *s* ≥ s* and (ii) s* is large enough to eliminate enrichments due to unspecific binding. Condition (i) is implemented by comparing $\hat{\sigma }$ and $\hat{\mu }$ for many choices of s*, while condition (ii) is implemented by plotting the counts *n*^2^(*x*) and *n*^3^(*x*) at the two successive cycles, as illustrated in S3 Fig (see also S24 Fig): sequences with *s* = *s*_us_ appear in the diagonal with a variance that decreases with increasing counts, as expected from sampling noise, and s* is chosen so as to exclude these sequences. Both criteria are usually simultaneously satisfied if the main source of deviations from lognormality is the presence of more than one binding mode (S28 Fig). In cases where specific binding to the target is very strong, sequences selected for unspecific binding are not present (S15(A) Fig), while in cases where specific binding is too weak, only sequences selected for unspecific binding are present (S15(F) Fig).

The same criteria apply when fitting to generalized Pareto distributions to infer the parameter *κ* but criterion (i) may lead to a higher value of s* if the measured enrichments extend beyond the tail of the distribution. In our previous work [16], we only considered criterion (i). In one case (Frog3 against DNA1), the s* that we define here by accounting for (ii) differs from the s* that we had previously defined (S15 Fig), which leads to a significantly different estimation of *κ*: $\hat{\kappa }=-0.53\pm 0.19$ instead of $\hat{\kappa }=0.97\pm 0.38$. In the other cases, we recover essentially the same results. The new analysis provides, however, additional insights; in the case of Frog3 against PVP, it thus appear that the vanishing value of *κ* can be attributed to the enrichments being dominated by unspecific binding (S15 Fig).

### Fit to log-normal distributions

To infer from experimental data the parameters *σ* and *μ* of a log-normal distribution, as given by Eq (1) in the Box, we focus on the best available enrichments *s*_*i*_ > s*. In practice, it is more convenient to work with the log of the enrichments, *y*_*i*_ = ln(*s*_*i*_), and to fit them with a normal distribution. If restricting to values *y*_*i*_ larger than a given threshold y*, the probability density *P*(*Y* = *y*|*Y* ≥ y*) of observing *y*_*i*_ given that *y*_*i*_ ≥ y* is

$$P(Y=y|Y\geq y^{*})=\frac{P(Y=y)}{P[Y\geq y^{*}]}=\sqrt{\frac{2}{\pi }}\frac{e^{-\frac{{\left(y-\mu \right)}^{2}}{2\sigma^{2}}}}{\sigma [1-\text{erf}\;(\frac{y^{*}-\mu }{\sqrt{2}\sigma })]},$$ (5)

where $\text{erf}\left(x\right)=\frac{2}{\sqrt{\pi }}\int_{0}^{x}e^{-\xi^{2}}d\xi$ is the Gauss error function. The log-likelihood $L(\mu ,\sigma ,y^{*})$ then verifies

$$\begin{array}{cc} -\frac{1}{N}L\left(\mu ,\sigma ,y^{*}\right) & =-\frac{1}{N}\sum_{i=1}^{N}\;\text{ln}\;P\left(Y=y_{i}|Y\geq y^{*}\right)\\ & =\text{ln}\left(\sigma \right)+\text{ln}[1-\text{erf}(\frac{y^{*}-\mu }{\sqrt{2}\sigma })]+\frac{1}{2\sigma^{2}N}\sum_{i=1}^{N}{\left(y_{i}-\mu \right)}^{2}, \end{array}$$ (6)

up to irrelevant additive constants independent of the parameters *μ* and *σ*. For a given y*, we minimize this quantity with respect to the parameters *σ* and *μ* to obtain $\hat{\sigma }\left(y^{*}\right)$ and $\hat{\mu }\left(y^{*}\right)$ and then chose y* such that for any *y* ≥ y* both $\hat{\sigma }\left(y\right)$ and $\hat{\mu }\left(y\right)$ are nearly constant (criterion (i) in previous section). Finally, we obtain a lower bound on the uncertainty of the parameter values using the Fisher information matrix and the Cramér-Rao bound. To assess the quality of fit, we produce P-P plots comparing the cumulative distribution of data to

$$\begin{array}{c} z=F\left(y|y^{*}\right)=P\left[Y\geq y|Y\geq y^{*}\right]=\frac{\text{erf}(\frac{y-\mu }{\sqrt{2}\sigma })-\text{erf}(\frac{y^{*}-\mu }{\sqrt{2}\sigma })}{1-\text{erf}(\frac{y^{*}-\mu }{\sqrt{2}\sigma })} \end{array}$$ (7)

where *z* is the fraction of the data above *y* ≥ y* according to the model, and Q-Q plots comparing the data to the inverse distribution function *y* = *F*^−1^(*z*|y*).

What may be expected in presence of unspecific binding is illustrated in S28 Fig with simulated data: consistent inferences of $\hat{\sigma }\left(y^{*}\right)$ and $\hat{\mu }\left(y^{*}\right)$ are obtained in an intermediate range of thresholds, while divergences may arise outside this range.

### Normalization of *μ* across libraries

The selection of a library *L* against a target *T* yields only the values of the highest enrichments *s*(*x*) up to an unknown multiplicative constant *α* (see Box). The parameter *σ* = *σ*_*L*,*T*_ is independent of *α* but not the parameter *μ* = *μ*_*L*,*T*_. The relative values of *μ*_*L*,*T*_ for different libraries *L* selected against the same target *T* are determined by performing selections where the different libraries are mixed in the initial population: this leaves undetermined one overall multiplicative constant per target which we fix by setting *μ*_Germ,*T*_ = 0 for each target *T*.

## Supporting information

S1 Text Supporting text with further description of the theoretical and experimental methods. (PDF)

S1 Table Parameters obtained from fits of the distribution of enrichments to generalized Pareto distributions (*κ*, *τ*) and log-normal distributions (*σ*, *μ*) for experiments presented here and in our previous work [16]. N/A indicates that the data was insufficient to make a meaningful fit. For enrichments against the protein targets between rounds *c* = 2 and *c* + 1 = 3, values are given for two independent replica of the experiment. The given uncertainties correspond to a single standard deviation around the maximum likelihood estimate as given by the Cramér-Rao bound. In the case of Frog3 against DNA1, and only in this case, the value of *κ* differs from the one reported in our previous work [16] for reasons explained in S15 Fig.
(TIF)

S1 Fig Alignment of the sequences of the three scaffolds, Bnab, Lim and Germ. The 4 randomized positions correspond to the part of the CDR3 indicated by XXXX.
(TIF)

S2 Fig DNA1 and DNA2 binding targets. The targets display a hairpin structure at room temperature. They share a common stem sequence but the sequence of their loop differ. A biotin is placed at the 5’ ends to allow for immobilization on streptavidin-coated magnetic beads.
(TIF)

S3 Fig Illustration of the choice of the cutoff s* below which measured enrichments are attributed to unspecific selection. The number *n*^3^(*x*) of counts in the sequencing data at round *c* = 3 is plotted against the number *n*^2^(*x*) of counts at round *c* − 1 = 2 for a selection of the Bnab library mixed with the two other libraries against the DNA1 target. An accumulation of sequences with similar enrichments is observed along the diagonal, with larger variance for smaller values as expected from an increased sampling noise. This is interpreted as arising from unspecific selection, e.g., through unspecific binding, associated with an enrichment *s*_us_ independent of the sequence. We define a cut-off s* such that sequences *x* with *s* = *n*^3^(*x*)/*n*^2^(*x*) ≥ s* cannot be attributed to unspecific selection. In addition, we restrict to sequences *x* with *n*^2^(*x*) ≥ 10 and *n*^3^(*x*) ≥ 10, as represented by the vertical and horizontal lines, to ensure that the inferred enrichments are not dominated by sampling noise.
(TIF)

S4 Fig Comparisons between results of replicate and non-replicate experiments. **A**. Comparison of the frequencies *f*^3^(*x*) = *n*^3^(*x*)/∑_*x*′_ *n*^3^(*x*′) computed after the third cycle (*c* = 3) between two independent replicate experiments where a mixture of the Germ (in blue), Lim (in green) and Bnab (in red) libraries is selected against the protein target prot1. Due to stochastic sampling, some sequences *x* are well represented in one experiment (*n*^3^(*x*) ≥ 10) but not in the other; they are represented by the points along the two axes. As expected, the frequencies of the most prevalent sequences are the most reproducible. **B**. As in A but for protein target prot2. **C**. Comparing an experiment with prot1 as target with another with prot2 as target: common sequences are enriched in the two cases, although with not exactly the same frequencies. **D**. Comparing an experiment with prot1 as target with another with DNA1 as target, showing that different sequences are enriched in each case. In particular, the most frequent sequences when selecting against one target are absent in the third round when selecting against the other (points along the axes). **E,F,G,H**. Comparison of enrichments *s*(*x*) calculated from the frequencies between the second and third rounds as *s*(*x*) = λ*n*^3^(*x*)/*n*^2^(*x*). Points along the axes correspond to sequences for which the enrichment could be estimated only for one of the two experiments. We verify that in cases E,F,G where the targets are similar the same top enrichments are recovered (up to a multiplicative constant corresponding to a shift in log-log plots). Beyond stochastic effects, reproducibility is mainly limited by the differences in the production of the targets, as shown in S12 Fig.
(TIF)

S5 FigExtension of Fig 5 to the 3 libraries Germ, Lim, Bnab selected either in a mixture (mix) or on their own (alone) against the DNA1 and DNA2 targets.The sequences logos represent the frequencies $f_{i}^{c}\left(a\right)$ of amino acids at each successive cycle *c* = 0, 1, 2, 3, 4.(TIF)

S6 FigExtension of Fig 5 to the 3 libraries Germ, Lim, Bnab selected in mixture against the prot1 and prot2 targets.The sequences logos represent the frequencies $f_{i}^{c}\left(a\right)$ of amino acids at each successive cycle *c* = 0, 1, 2, 3, 4. The data is presented at two different scales for better readability.(TIF)

S7 FigSequence logos for the enrichments $\tilde{s}\left(x\right)$ computed between two successive rounds (1-2, 2-3 or 3-4).The differences between rounds reflect sampling fluctuations.(TIF)

S8 Fig Fitting distributions of top enrichments with log-normal distributions. Top: separate selections of the Germ and Lim libraries against the DNA1 target. Here enrichments are computed between rounds 1 and 2. Note that the *μ* cannot be compared and is fixed to *μ* = 0 in both cases. These experiments complement those of Fig 1 where the libraries are selected together, which does not leave sufficient data for the analysis of the Lim library against the DNA1 target. Bottom: analyses of replicate experiments where the three libraries are jointly selected against the two protein targets, as in the bottom panels of Fig 1.
(TIF)

S9 Fig How the estimation of the entropy is biased by finite sampling. 10^5^ values were drawn from a log-normal distribution with parameters *μ* = 0 and *σ* = 0.5 (green), 1 (red) and 1.5 (blue). The relative entropy *D*(*P*_1_‖*P*_0_) was then estimated using a random subsample of size *N*. For any *N* < 10^5^, this leads to an overestimation of *D*(*P*_1_‖*P*_0_) whose actual value *σ*^2^/2 (see Eq. 14 in S1 Text) is represented by the horizontal lines at the bottom.
(TIF)

S10 FigSequence logos for the enrichments ${\tilde{s}}_{i}\left(a\right)$ of the Bnab library subject to either amplification only or to amplification and selection for binding against the DNA1, DNA2, prot1 or prot2 targets.The enrichments are computed between the first and second cycles (1-2) or between the third and fourth cycles (3-4); for amplification only, the results of two replicate experiments are shown. The sequence logos of enrichments calculated between rounds 2 and 3 are the same as those shown in Fig 2 (Bnab library), except for the scale along the y-axis. All sequences logos share common patterns reflecting a common contribution from amplification biases. Sequence logos against the protein targets show, however, an enrichment for tryptophane (symbol W) that is not observed when selection involves amplification only. Selections of the Bnab library thus have a target-dependent contribution from binding affinity of similar order of magnitude as a common target-independent contribution from amplification biases.(TIF)

S11 Fig Contribution of amplification biases to the enrichments in selection against the DNA1 target. A separate experiment without any selection for binding was performed to estimate the difference of enrichments arising from the amplification step alone. **A**. The resulting *s*_amplif_ is here compared to the enrichments *s*_tot_ from an experiment including a selection for binding. The sequences with top *s*_tot_, which all belong to the Germ library (in blue), are among the sequences with lowest *s*_amplif_, which indicate that they are selected for binding with no contribution from the amplification bias. On the other hand, the sequences with top *s*_tot_ from the Lim and Bnab libraries (respectively in green and red), have also top *s*_amplif_, which indicate a significant contribution from amplification biases. **B**. The ratio *s*_tot_/*s*_amplif_ represents the contribution to enrichment of binding alone. The two selective pressures, binding and amplification, appear here to be orthogonal.
(TIF)

S12 Fig Supplementary experiments with minimal libraries. **A**. Enrichments of top and random sequences from the three libraries, Germ (in blue), Lim (in green) and Bnab (in red), against DNA1. **B**. Results from a replicate experiment using a different stock of beads, showing that the enrichments are reproduced except for the Bnab sequences (in red), which have a systematically higher enrichment. **C**. Similar to A, but when selecting for binding to the beads in absence of the DNA1 target. The top enrichments are from the Bnab sequences (in red), indicating that they bind to the beads, a finding consistent with the discrepancy between A and B. Here, the differences in enrichments are also coming from differences of enrichment during amplification (S11 Fig). Consistent with S11 Fig, the top Germ sequences (blue dots) have in absence of the DNA1 target the worst enrichments.
(TIF)

S13 Fig Cross selections with minimal libraries consisting of mixtures of top sequences against the DNA1 target (full circles) and top sequences against the DNA2 target (full crosses). **A,C**. Selection against the DNA1 target (same as in S12 Fig). **B,D**. Selection against the DNA2 target. The results confirm that some sequences from the Germ and Lim libraries bind specifically to the DNA1 target (blue dots and one of the green dots) and some sequences from the Germ library to the DNA2 target (blue crosses).
(TIF)

S14 FigRelation between the parameter *σ* from log-normal fits and the parameter *κ*_*N*_ from generalized Pareto fits from numerical simulations.**A**. *N* = 10^4^ values were drawn from a log-normal distribution with parameters *μ* = 0 and varying *σ* (x-axis). The largest 25, 50, 75, 100% of these values (i.e., 75, 50, 25, 0% truncation) were fitted to a Pareto model with parameters *κ* and *τ*. The plot shows the estimation $\hat{\kappa }$ as a function of *σ*. Averages and standard deviations are taken over 25 independent realizations of the numerical experiment. It shows that limited sampling may cause a $\hat{\kappa }<0$ to be inferred from values drawn from a log-normal distribution when *σ* is small, here *σ* < 0.5. **B**. Inverse simulation: A truncated log-normal model is fitted to the largest 25, 50, 75, 100% among 500 values (i.e., 75, 50, 25, 0% truncation) drawn from a Pareto model with parameters *τ* = 0.115, s* = 0.001 and varying *κ* (x-axis). The black dotted line in Fig 4 corresponds to the 25% truncation.(TIF)

S15 Fig Definition of the threshold s* above which enrichments *s* are considered for the experimental results reported here (A) and in Ref. [16] (B-F). As in S3 Fig, the definition is based on a comparison between counts at the 2nd and 3rd cycles. The horizontal and vertical lines correspond to the criteria *n*^2^(*x*) ≥ 10 and *n*^3^(*x*) ≥ 10. The plain oblique line corresponds to the definition of s* in this work. In the case of the selection of the Frog3 library against the DNA1 target, it differs from the value of s* used in our previous work [16] (dotted oblique line) which failed to discard many enrichments coming from unspecific binding. In the case of the selection of the Frog3 library against the PVP target, all measured enrichments may be attributed to unspecific binding and we are therefore not including the inferred values of *σ* and *κ* in Fig 4.
(TIF)

S16 Fig Assessments of the qualities of the fits of the enrichments to generalized Pareto distributions (cyan) and to log-normal distributions (black) for selections of the Germ library. The different graphs correspond to selections against different targets. For the protein targets prot1 and prot2, results from two replicate experiments are presented. All enrichments are computed by comparing the frequencies at the 2nd and 3rd cycle. The graphs on the right show the P-P and Q-Q (inset) plots for each fit. Perfect fits would correspond to the red dotted lines.
(TIF)

S17 Fig Same as S16 Fig but for the Lim library instead of the Germ library. (TIF)

S18 Fig Same as S16 Fig but for the Bnab library instead of the Germ library. (TIF)

S19 Fig Same as S16 Fig for the Germ library selected in isolation rather in a mixture with the two other libraries. (TIF)

S20 Fig Same as S16 Fig but for enrichments computed from a comparison between the 3rd and 4th cycle instead of the 2nd and 3rd cycle. (TIF)

S21 Fig Same as S20 Fig (enrichments computed from a comparison between the 3rd and 4th cycle) but for the Bnab library instead of the Germ library. (TIF)

S22 Fig Same as S20 Fig but for the experimental results reported in Ref. [16]. (TIF)

S23 FigAnalysis of data from the Germ library selected against the DNA1 target (in Mix) with the stochastic model presented in S1 Text, Sec 1.5.The data consists in the counts *n*^1^(*x*), *n*^2^(*x*), *n*^3^(*x*) at the different rounds (panels C and F), from which enrichments are inferred in different ways that we compare. As in the main text, we define *s*^1- 2^(*x*) ∝ *n*^2^(*x*)/*n*^1^(*x*) when *n*^1^(*x*) ≥ 10 and *n*^2^(*x*) ≥ 10, and *s*^2-3^(*x*) ∝ *n*^3^(*x*)/*n*^2^(*x*) when *n*^2^(*x*) ≥ 10 and *n*^3^(*x*) ≥ 10: they are shown in panel G to give consistent results (undefined values are represented as small values). Alternatively, we can infer enrichments by maximum likelihood using the model in section 1.5 of S1 Text. For each successive rounds *c*-(*c* + 1) with *c* = 1 or 2, two solutions are considered: $s_{0}^{c-(c+1)}\left(x\right)$ where unspecific binding is neglected (Δ*G*_us_ = ∞) and $s_{1}^{c-(c+1)}\left(x\right)$ where it is not (Δ*G*_us_ treated as variable in addition to the *h*_*i*_(*a*)). They are compared to *s*^*c*-(*c*+1)^ in panels A, B, D, E. In B and E, where unspecific binding is present, the sequences that are predicted to be selected through specific binding ($e^{-\beta G(x)}>e^{-\beta G_{us}}$ Eq. 21 of S1 Text) are represented in orange. When considering data between rounds 1- 2, a good agreement is found between *s*^1-2^(*x*) and $s_{1}^{1-2}\left(x\right)$ (panel B) and the sequences identified as binding specifically (in orange) correspond indeed to those above a threshold, *s*^1-2^(*x*) > s*(panel C). This is not the case when considering the data between rounds 2-3 where the model predicts many sequences with high enrichments $s_{1}^{2-3}\left(x\right)$ that are not reported in *s*^2-3^(*x*) (panel E). In this case, the solution without non-specific binding $s_{0}^{2-3}\left(x\right)$ appears to be more relevant. This is confirmed in panels H and I where *s*^1-2^(*x*) is seen to correlate better with $s_{0}^{2-3}\left(x\right)$ than with $s_{1}^{2-3}\left(x\right)$. Panel J represents the maximum value of the log-likelihood for fixed values of Δ*G*_us_, showing the presence of a non-trivial optimum (data from rounds 1-2). The fields *h*_*i*_(*a*) of this model are shown in panel K in the zero-sum gauge where $\sum_{a=1}^{q}h_{i}\left(a\right)=0$ for all *i*. The same information can also be represented in the form a sequence logo (panel L), to be compared to the sequence logo obtained from *s*(*x*) (Fig 2B, Germ-DNA1).(TIF)

S24 Fig Relative frequencies at round 1 (x-axis) and round 2 (y-axis) of sequences from the 3 libraries, Germ (blue), Lim (green) and Bnab (red) when selected in mixture against the DNA1 target. This figure shows that each library has a different background noise.
(TIF)

S25 Fig Reproducibility of enrichments inferred from experiments with mini-libraries. **A**. Enrichments from S13(A) Fig versus S13(C) Fig: the results from the two experiments are highly reproducible except for the Bnab sequences in red. This difference is due to the different batches of beads used in these two experiments. **B**. Enrichments from S13(B) Fig versus S13(D) Fig. Here the two experiments use the same batch of beads and the inferred enrichments are all very reproducible. **C**. Enrichments from S12(B) Fig versus S12(A) Fig, showing again high reproducibility. Error bars are enlarged 20 times to make them visible.
(TIF)

S26 FigDependence of the inferred values of $\hat{\kappa }$, when fitting the tail of the distribution of enrichments to a generalized Pareto distribution, and $\hat{\sigma }$, when fitting them to a truncated log-normal distribution, on the choice of the threshold s* or y* = ln(s*) that defines the tail.Here for the Germ library selected against different targets. When the threshold is too large, very few data points are left and the error bars, obtained from the Fisher information matrix via the Cramér-Rao bound, are large. In any case, however, the estimation of $\hat{\kappa }$ and $\hat{\sigma }$ is consistent across a range of values of the thresholds.(TIF)

S27 Fig Similar to S26 Fig but for the Lim and Bnab libraries. (TIF)

S28 Fig Inference from simulated data, in analogy to S12 and S27 Figs. The 3 examples correspond to different draws of *N* = 10^4^ samples from a mixture model with two equiprobable modes: a bottom (“unspecific”) mode described by a lognormal distribution with parameters *μ*_us_ = −10 and *σ*_us_ = 1 and a top mode described by a lognormal distribution with parameters *μ* = −9 and *σ* = 1. The parameters of this top mode are recovered for an intermediate range of thresholds. For too small thresholds, the presence of the bottom mode leads to inconsistent values while for too high thresholds the number of samples becomes insufficient.
(TIF)

S1 Code Jupyter notebooks with code to reproduce our analysis. (RAR)
