# Supplementary material for: Parameters and determinants of responses to selection in antibody libraries
Source: PLoS Comput Biol. 2021 Mar 25;17(3):e1008751. doi: 10.1371/journal.pcbi.1008751 (PMC7993935; doi:10.1371/journal.pcbi.1008751)
Supplement: S1 Text — (PDF) [file pcbi.1008751.s001.pdf]

SUPPLEMENTARY APPENDIX

Parameters and determinants of responses to selection in antibody libraries

Steven Schulz[a], Sébastien Boyer[b], Matteo Smerlak[c], Simona Cocco[d], Rémi Monasson[d], Clément Nizak[e], and Olivier Rivoire[a]

[a] *Center for Interdisciplinary Research in Biology (CIRB), Collège de France, CNRS UMR 7241, INSERM U1050, PSL University, Paris, France*
[b] *Département de biochimie, Faculté de Médecine, Université de Montréal, Montréal, Canada*
[c] *Max Planck Institute for Mathematics in the Sciences, Leipzig, Germany*
[d] *Laboratory of Physics of École Normale Supérieure, UMR 8023, CNRS & PSL University, Paris, France*
[e] *Chimie Biologie Innovation, ESPCI Paris, CNRS, PSL University, Paris, France*

Contents

1 Theoretical methods **2**
1.1 Physics of selection . . . 2
1.1.1 Enrichments and binding energies . . . 2
1.1.2 Justification and limitations of log-normal distributions . . . 3
1.2 Alternative statistical model from extreme value theory . . . 3
1.2.1 Extreme value statistics . . . 3
1.2.2 Effective shape parameter of log-normal distributions . . . 4
1.2.3 κ versus σ in the data . . . 4
1.3 Information theory of selection . . . 4
1.3.1 Relative entropies . . . 4
1.3.2 Information theory of specific interactions . . . 5
1.3.3 Equivalence with the parameter σ . . . 6
1.3.4 Sequence motifs . . . 6
1.4 Dynamics of selection . . . 6
1.4.1 Recursion for the sequence frequencies . . . 6
1.4.2 Recursion for the library frequencies . . . 7
1.5 Noise cleaning with a stochastic model . . . 7

2 Experimental methods **8**
2.1 Phage production . . . 8
2.2 Target immobilization . . . 9
2.3 Phage display selection . . . 9
2.4 Illumina sequencing . . . 10
2.5 Data preprocessing . . . 10

1. Theoretical methods

1.1. Physics of selection

1.1.1. Enrichments and binding energies

When assuming that selection is controlled by equilibrium binding to the target, the distribution of enrichments is constrained by physical principles. Starting with a population of identical antibodies A and a single target T in excess relative to antibodies, $[T]_{\rm tot} \gg [A]_{\rm tot}$, the probability for an antibody to be bound to a target is

$$s_{AT} = \frac{[AT]_{\rm eq}}{[AT]_{\rm eq} + [A]_{\rm eq}} = \frac{1}{1 + K_{AT}[T]_{\rm eq}^{-1}} \simeq \frac{1}{1 + K_{AT}[T]_{\rm tot}^{-1}} \quad (1)$$

where $[AT]_{\rm eq}$ and $[A]_{\rm eq}$ are, respectively, the equilibrium concentration of bound and free antibodies and where $K_{AT} = [A]_{\rm eq}[T]_{\rm eq}/[AT]_{\rm eq}$ is the dissociation constant that characterizes the equilibrium. We used here the fact that most of the targets are unbound so that $[T]_{\rm eq} = [T]_{\rm tot} - [AT]_{\rm eq} \simeq [T]_{\rm tot}$, which is justified for our experiments where the total number of targets far exceeds the total number of antibodies, $[AT]_{\rm eq} < [A]_{\rm tot} \ll [T]_{\rm tot}$. The dissociation constant can also be written as $K_{AT} = k_-/k_+$, where k_+ and k_- denote respectively the association and dissociation rates of an antibody-target pair.

We can equivalently write

$$s_{AT} = \frac{1}{1 + {\rm e}^{\beta(\Delta G_{AT} - \mu)}} \quad (2)$$

by introducing a binding free energy $\Delta G_{AT} = \beta^{-1} \ln K_{AT}$ and a chemical potential $\mu = \beta^{-1} \ln [T]_{\rm tot}$, where β sets the energy scale [1]. This Fermi-Dirac statistics is approximated by Boltzmann statistics

$$s_{AT} \simeq {\rm e}^{-\beta(\Delta G_{AT} - \mu)}. \quad (3)$$

when $\Delta G_{AT} \gg \mu$. This approximation is justified when $[T]_{\rm tot} \ll K_{AT}$ or, equivalently, $[AT]_{\rm eq} \ll [A]_{\rm eq}$, i.e., when the concentration of the targets or the binding affinity are sufficiently low for most of the antibodies to be unbound. Working in this regime is important for the enrichments to reflect binding free energies. Otherwise, the targets are saturating, which cause antibodies to be bound with high probability irrespectively of their dissociation constant.

These conclusions are unchanged when considering a population consisting of different antibodies A with different dissociation contants K_{AT} and binding free energies $\Delta G_{AT} = \beta^{-1} \ln K_{AT}$. In summary, when considering different antibodies A, each with its own dissociation constant K_{AT}, the choice of the target concentration $[T]_{\rm tot}$ is subject to the two constraints

$$\sum_A [A]_{\rm tot} \ll [T]_{\rm tot} \ll \min_A K_{AT}. \quad (4)$$

The first constraint $\sum_A [A]_{\rm tot} \ll [T]_{\rm tot}$ guarantees an absence of competition between antibodies so that the enrichments s_{AT} are intrinsic properties of the sequences of A, independent of the composition of the population and therefore independent of the round c when successive cycles of selection are performed; formally, $[T]_{\rm eq}$, which depends on all A present, can then be replaced by $[T]_{\rm tot}$ in Eq. (1). The second constraint $[T]_{\rm tot} \ll \min_A K_{AT}$ guarantees that even the best binders are not in a saturation regime with $s_A \simeq 1$ independently of differences in their dissociation constants K_{AT}. In our phage display experiments,

$\sum_A [A]_{\rm tot} \simeq 10^{11}\,{\rm mL}^{-1}$ and $[T]_{\rm tot} \simeq 10^{14}\,{\rm mL}^{-1}$, which satisfies the first constraint. The concentration $\sum_A [AT]_{\rm eq}$ of selected antibodies before amplification is estimated between $10^5\,{\rm mL}^{-1}$ at the first round of selection and $10^7 - 10^8\,{\rm mL}^{-1}$ at the fourth. Considering this last number to reflect properties of the best binders, we estimate that $\min_A K_{AT}/[T]_{\rm tot} \simeq \sum_A [A]_{\rm tot}/\sum_A [AT]_{\rm eq} \simeq 10^3$, which satisfies the second constraint.

1.1.2. Justification and limitations of log-normal distributions

Assuming an additive model for the interaction where the binding energy between sequence $x = (x_1, \dots, x_\ell)$ and its target takes is of the form $\Delta G(x) = \sum_{i=1}^{\ell} h_i(x_i)$ with the $h_i(x_i)$ taking random values, the central limit theorem indicates that for sufficiently large ℓ the energies $\Delta G(x)$ are distributed normally with a mean $\mu \simeq -\ell \langle h \rangle$ and a variance $\sigma^2 \simeq \ell(\langle h^2 \rangle - \langle h \rangle^2)$, where $\langle h \rangle$ and $\langle h^2 \rangle - \langle h \rangle^2$ are respectively the mean and variance of the values of binding energies per position $h_i(x_i)$. Given Eq. (3), this leads to a log-normal distribution for the enrichments $s(x) \propto e^{-\beta \Delta G(x)}$.

The assumptions involved in this derivation may not be justified, starting from the assumption that enrichment can be equated to binding affinity. However, essentially all deviations from this model, sequence-dependent amplification differences, saturation of the targets, multiple binding sites or non-additive interactions, can be incorporated in a more refined model, at the expense of introducing additional parameters [2]. Deviations from a log-normal distribution of enrichments can therefore, at least in principle, be systematically analyzed and understood.

1.2. Alternative statistical model from extreme value theory

In our previous work [3], we fitted the tail of the distribution of enrichments with generalized Pareto distributions from extreme value theory. For different libraries L and different targets T, we found that generalized Pareto distributions provide a good fit of the upper tail of the distribution of enrichments, with, depending on the scaffold L and target T either $\kappa > 0$ (heavy tail), $\kappa < 0$ (bounded tail) or $\kappa = 0$ (exponential tail). The origin of these different values of κ was, however, unclear. Here, we show that κ captures essentially the same information as σ, one of the two parameters of the model based on the log-normal distribution.

1.2.1. Extreme value statistics

Extreme value theory states that for any random variable S, the probability to have $S = s \geq s^\star$ conditioned to $S \geq s^\star$ converges to a generalized Pareto distribution $f_{\kappa,s^\star,\tau}(s) = \tau^{-1} f_\kappa\left((s - s^\star)/\tau\right)$ as $s^\star \to \infty$ [4], where

$$f_\kappa(x) = \begin{cases} (1 + \kappa x)^{-\left(1+\frac{1}{\kappa}\right)} & \text{if} \quad \kappa \neq 0, \\ \mathrm{e}^{-x} & \text{if} \quad \kappa = 0. \end{cases} \tag{5}$$

The shape parameter κ is determined by the tail of the distribution of S. In particular, $\kappa < 0$ for bounded distributions and $\kappa = 0$ for distributions with exponentially decreasing tails, including log-normal distributions. On the other hand, $\kappa > 0$ for distributions whose tail decays as a power-law. For such distributions, when considering a large number N of random values $s_1 > s_2 > \cdots > s_N$, $s_r \sim s_1 r^{-\kappa}$ for $r \ll N$, which is represented in a log-log plot of s_r versus the rank r by the linear relationship $\ln(s_r/s_1) \sim -\kappa \ln r$ for the smallest values of r.

1.2.2. Effective shape parameter of log-normal distributions

In the asymptotic limit where $N \to \infty$ followed by $s^\star \to \infty$, log-normal distributions are described by a shape parameter $\kappa = 0$, but their tail decays only slowly. As a result, a large but finite number N of random values drawn from a log-normal distribution may appear to be drawn from a distribution with a non-zero shape parameter $\kappa_N \neq 0$.

More precisely, it can be shown that N values $s_1 > s_2 > \cdots > s_N$ drawn from a log-normal distribution with parameters σ, μ satisfy for $r \ll N$ the relation

$$\mathbb{E}[\ln s_r] \simeq \mu + \left((2\ln N)^{1/2} - \frac{\ln\ln N + \ln 4\pi}{2\sqrt{2\ln N}}\right)\sigma - \frac{\sigma}{(2\ln N)^{1/2}}\ln r, \tag{6}$$

which corresponds to an apparent shape parameter $\kappa_N = \sigma(2\ln N)^{-1/2}$ [5]. As κ_N vanishes only very slowly with N, it is difficult to determine whether N data points arise from a log-normal distribution or from a distribution with a shape parameter $\kappa > 0$. For instance, increasing the sample size from $N = 10^5$ to $N = 10^6$ changes κ_N by only 8 %.

Eq. (6) itself assumes that N is large enough. Numerically, we observe that for a given value of N, it breaks down when σ is below some value $\sigma^\star$. In such cases, the data may appear to arise from a bounded distribution with $\kappa_N < 0$. S14 Fig shows the relationship between κ_N and σ obtained from numerical simulations when fixing $N = 10^4$ and $\mu = 0$, in which case $\sigma^\star \simeq 0.5$. The same relationship appears as a black dotted line in Fig 4.

1.2.3. κ versus σ in the data

Comparing probability-probability plots to assess the quality of the fits, our data appear equally well fitted by generalized Pareto distributions and log-normal distributions (S16 Fig to S22 Fig). These results are consistent with theoretical expectations. The (effective) shape parameter κ from extreme value theory and the parameter σ from log-normal distributions thus report essentially the same information (Fig 4). The data thus support the hypothesis that maturation lead to a loss of selective potential irrespective of the statistical model used to quantify selective potentials, whether it is a generalized Pareto distribution motivated by extreme-value theory or a log-normal distribution.

1.3. Information theory of selection

Several relationships are known between evolutionary dynamics and information theory. In particular, the change of Malthusian fitness $\ln s(x)$ satisfies [8]

$$\Delta \ln s = D(\tilde{s} \| N^{-1}) + D(N^{-1} \| \tilde{s}). \tag{7}$$

Here we present a different relationship, which also involves the relative entropy $D(\tilde{s}\|N^{-1})$. This new relationship extends the work of Ref. [9].

1.3.1. Relative entropies

A general statistical approach to quantify how random variables drawn from a probability P^1 are consistent with a reference probability distribution P^0 is to use their relative entropy $D(P^1\|P^0)$, also known

as the Kullback-Leibler divergence [7], which is defined by

$$D(P^1\|P^0)=\sum_x P^1(x)\ln\frac{P^1(x)}{P^0(x)}. \tag{8}$$

The inverse of this quantity corresponds roughly to the number of samples required to discriminate P^1 from P^0. More precisely, the probability under P^0 of N samples drawn from P^1 scales as $e^{-ND(P^1\|P^0)}$ [7].

1.3.2. Information theory of specific interactions

The problem of quantifying specificity arises when two classes of objects or properties A and T may be associated. If this association is described by the probability $P^1(A,T)$ that A is associated with T, a natural measure of specificity is $D(P^1\|P^0)$ where $P^0(A,T)$ represents the expectation from random associations. If $P^0(A,T)=P^1(A)P^1(T)$ where $P^1(A)=\sum_T P^1(A,T)$ and $P^1(T)=\sum_A P^1(A,T)$ are the marginal distributions of A and T, $D(P^1\|P^0)$ corresponds to the mutual information $I(A;T)$ between the random variables A and T [7]. This choice of P^0, however, generally does not reflect the expectation from random associations and the relevant measure of specificity is therefore generically not captured by a mutual information but by the more general relative entropy $D(P^1\|P^0)$.

In the case of association between a set of ligands A and a set of targets T controlled by equilibrium binding, the probability $P^1(A,T)$ to find A bound to T is

$$P^1(A,T)=\frac{[AT]_{\rm eq}}{[A]_{\rm eq}+\sum_{T'}[AT']_{\rm eq}}\simeq\frac{[AT]_{\rm eq}}{[A]_{\rm eq}}=K_{AT}^{-1}[T]_{\rm eq}\simeq K_{AT}^{-1}[T]_{\rm tot} \tag{9}$$

where K_{AT} is the dissociation constant between A and T and where the approximations are justified in Section 1.1. A random association is defined here by considering equal dissociation constants,

$$P^0(A,T)=\frac{[A]_{\rm tot}[T]_{\rm tot}}{\sum_{A',T'}[A']_{\rm tot}[T']_{\rm tot}}. \tag{10}$$

This distribution generally differs from $P^1(A)P^1(T)$.

A enrichment s_{AT} can be defined for each pair A,T as $s_{AT}=P^1(A,T)/P^0(A,T)$ so that

$$D(P^1\|P^0)=\left\langle\ln\left(\frac{P^1}{P^0}\right)\right\rangle_1=\sum_{A,T}P^1(A,T)\ln\frac{P^1(A,T)}{P^0(A,T)}=\sum_{A,T}P^0(A,T)s_{AT}\ln s_{AT}=\langle s\ln s\rangle_0 \tag{11}$$

where $\langle\cdot\rangle_0$ and $\langle\cdot\rangle_1$ denote averages taken with $P^0(A,T)$ and $P^1(A,T)$ respectively.

More generally, $s_{AT}=\lambda P^1(A,T)/P^0(A,T)$ with an arbitrary multiplicative constant λ that can always be written $\lambda=\langle s\rangle_0$. This corresponds to replacing s by $s/\langle s\rangle_0$ in the previous formula,

$$D(P^1\|P^0)=\left\langle\frac{s}{\langle s\rangle_0}\ln\frac{s}{\langle s\rangle_0}\right\rangle_0 \tag{12}$$

When a single target T is considered with $P^0(A,T)=1/N$ and $P^1(A,T)=s(x)$ where x represents the sequence of A, this becomes

$$D(\tilde{s}\|N^{-1})=\left\langle\frac{s}{\langle s\rangle}\ln\frac{s}{\langle s\rangle}\right\rangle \tag{13}$$

Eq. (12) is valid for any initial distribution $f^0(x)$ as long as $f^1(x) \propto s(x)f^0(x)$ while Eq. (13), where averages $\langle \cdot \rangle$ are taken with a distribution $P(s)$ of the enrichments over the different sequences x, is valid only when considering as initial distribution a uniform distribution over the sequences. The notation $D(s\|N^{-1})$ assumes, besides, that $\sum_x s(x) = 1$ so that $s(x)$ can be interpreted as a probability distribution.

1.3.3. Equivalence with the parameter σ

If further assuming that $P(s)$ is a log-normal distribution with parameters σ and μ, $\langle s \rangle = e^{\mu+\sigma^2/2}$ and $\langle s \ln s \rangle = \langle s \rangle (\mu + \sigma^2)$ so that

$$D(s\|N^{-1}) = \frac{\sigma^2}{2} \tag{14}$$

irrespectively of the value of μ. This reflects the fact that specificity quantifies only relative differences in binding free energies between different ligands.

A previous study proposed the mutual information as a measure of specificity [9]. It is justified, however, only within the special model considered in [9] where, because of the overall symmetry of the interactions between the M locks A and M keys T, $P^1(A) \simeq P^1(T) \simeq 1/M$, and therefore $P^0(A,T) = 1/M^2 \simeq P^1(A)P^1(T)$.

1.3.4. Sequence motifs

Assuming that the different sites i along the sequence contribute independently to the enrichment, $\tilde{s}(x) = \prod_i \tilde{s}_i(x_i)$, the specificity $D(\tilde{s}\|N^{-1})$ is nothing but $\sum_i D(\tilde{s}_i\|A^{-1}) = \sum_i \sum_{a_i} \tilde{s}_i(a_i) \ln[\tilde{s}_i(a_i)A]$, the total area under the sequence logos of $\tilde{s}_i(a)$, where $A = 20$ is the total number of amino acids. By displaying both amino acid specificities and an overall measure of specificity of selection $D(\tilde{s}\|N^{-1})$, sequence logos thus provide a convenient summary of selection within a library.

This comes, however, with an important caveat when enrichments are available only for a small subset of $N' \ll N$ sequences, as it is the case in experiments. If ignoring unobserved sequences when computing $\tilde{s}_i(a_i)$, the empirically determined quantity $\sum_i D(\tilde{s}_i\|A^{-1})$ overestimates the true value of $D(\tilde{s}\|N^{-1})$, all the more as N' is smaller (S9 Fig). Because of this effect, the areas under the curve of the sequence logos based on $\tilde{s}_i(a)$ are not comparable to $\sigma^2/2$ as Eq. (14) would suggest. They are also not comparable across different experiments when the sampling sizes N' differ (Fig 2B and C). Finally, even with $N' = N$, deviations between $\sum_i D(\tilde{s}_i\|A^{-1})$ and $D(\tilde{s}\|N^{-1})$ may arise if the contributions of the different positions are not additive.

1.4. Dynamics of selection

1.4.1. Recursion for the sequence frequencies

If $n^c(x)$ denotes the number of copies of sequence x at cycle c, the dynamics of selection satisfies the recursion

$$n^c(x) = \alpha_c s(x) n^{c-1}(x) \tag{15}$$

where α_c represents an amplification factor to reach at every round the same total population size N, i.e., $\sum_x n^c(x) = N$ independent of c. In terms of frequencies $f^c(x) = n^c(x)/N$, this gives $\alpha_c = (\sum_x s(x)f^c(x))^{-1}$ and

$$f^c(x) = \frac{s(x)f^{c-1}(x)}{\sum_{x'} s(x')f^{c-1}(x')} = \frac{(s(x))^c f^0(x)}{\sum_{x'} (s(x'))^c f^0(x')}. \tag{16}$$

These recursions assume a large N, so that the frequencies $f^c(x) = n^c(x)/N$ are meaningful; in particular, they assume that no sequence disappears.

Note the similarity with a Boltzmann distribution with the cycle c playing the role of an inverse temperature.

1.4.2. Recursion for the library frequencies

When considering a population consisting of an equal mix of different libraries L, the frequency $f^c(L) = \sum_{x\in L} f^c(x)$ of library L satisfies the recursion

$$f^c(L) = \frac{\langle s^c\rangle_L}{\sum_{L'}\langle s^c\rangle_{L'}} \tag{17}$$

with

$$\langle s^c\rangle_L = \sum_{x\in L}(s(x))^c f^0(x) = \int_0^\infty \mathrm{d}s\, P_L(s)s^c = \exp\left(c\mu_L + \frac{c^2\sigma_L^2}{2}\right). \tag{18}$$

Here, the first equality defines the average $\langle\cdot\rangle_L$ within each library L. The second equality, on the other hand, makes two assumptions: first, that enrichments s within library L are described by a distribution of enrichments $P_S(s)$ and, second, that sequences within a library are uniformly represented in the initial population. The third equality makes the additional assumption that $P_L(s)$ is a log-normal distribution with parameters σ_L and μ_L.

Under these different assumptions, the frequency of library L at cycle c is given by

$$f^c(L) = \left(\sum_{L'} \mathrm{e}^{c(\mu_{L'}-\mu_L)+c^2\left(\sigma_{L'}^2-\sigma_L^2\right)/2}\right)^{-1}. \tag{19}$$

This shows that for small c, the dynamics is controlled by the μ_L, with in limit $c\to 0$, $(f^c(L)-f^0(L))/f^0(L) \simeq c(\mu_L - \langle\mu\rangle)$, i.e., at the first cycle, the frequency of library L increases if its μ_S exceeds the average $\langle\mu\rangle$ across libraries and it decreases otherwise. For large c, on the other hand, the dynamics is controlled by the σ_Ls with $f^c(L)\to 1$ for the library L that has largest σ_L, regardless of the values of μ_L.

These calculations rely on several assumptions, in particular the assumption that sequences within a library have initially uniform frequencies, which is not satisfied in the experiments. This explains the differences between the model and the data in Fig 3.

1.5. Noise cleaning with a stochastic model

Another approach was previously proposed to clean the noise when analyzing data comparable to ours, which introduces a stochastic model for the sequencing bias and for the mapping $x \mapsto \Delta G(x)$ [2, 10]. We illustrate here how it gives results consistent with those of our simpler approach.

In this alternative approach, the sampling noise from sequencing noise is described by a Poissonian distribution (a description that may be elaborated to take into account the non-Poissonian effects of PCR amplification [11]). Given reads between two successive rounds $\{n^{c-1}(x)\}$, $\{n^c(x)\}$, the log-likelihood of the enrichments $s(x)$ has the form [10]

$$\mathcal{L}(s(x)|\{n^{c-1}(x)\},\{n^c(x)\}) = \sum_x n^c(x)\ln s(x) - (n^{c-1}(x)+n^c(x))\ln(1+s(x)) \tag{20}$$

where the sum is over all sequences x. Optimizing over $s(x)$ for each x independently gives $\hat{s}(x) = n^c(x)/n^{c-1}(x)$, consistent with Eq. (4) in the Box.

To identify specific binding, the relation between $s(x)$ and $\Delta G(x)$ must be specified. In the approximation of weak binding (see Sec. 1.1), $s(x)$ takes the form

$$s(x) = e^{-\beta\Delta G(x)} + e^{-\beta\Delta G_{\rm us}}. \tag{21}$$

To differentiate specific from unspecific binding, we further assume that $\Delta G(x)$ takes the form [2, 10]

$$\beta\Delta G(x) = \sum_{i=1}^{\ell} h_i(x_i), \tag{22}$$

which corresponds to ignoring epistasis between the sites i. With $\ell = 4$ denoting the length of the variable sequence x and $q = 20$ denoting the number of possible amino acids, this model has one parameter $\Delta G_{\rm us}$ for unspecific binding and ℓq parameters $h_i(x_i)$ for specific binding, of which only $\ell(q-1)+1 = 77$ are independent due of the invariance under the transformation $h_i(a) \leftarrow h_i(a)+g_i$ for any g_i satisfying $\sum_{i=1}^{\ell} g_i = 0$.

These parameters are obtained from the data by optimizing the log-likelihood given in Eq. (20). In practice, we find as in Ref. [2] that introducing a small ℓ_2 regularization on the fields $h_i(x_i)$ is necessary to prevent them from taking excessively large values. Two sets of parameters are of interest: the parameters maximizing the log-likelihood when $e^{-\beta\Delta G_{\rm us}} = 0$, corresponding to a model $s_0(x)$ without unspecific binding, and the parameters maximizing the log-likelihood with $\Delta G_{\rm us}$ treated as a variable, corresponding to a model $s_1(x)$ integrating unspecific binding. Which solution is most relevant depends on whether unspecific binding is negligible or not. If unspecific binding is negligible, $s_1(x)$ tends to under-fit the data while if unspecific binding is not negligible, $s_0(x)$ tends to over-fit it.

This is demonstrated in S23 Fig with the example of the Germ library selected against the DNA1 target, where unspecific binding is significant between rounds 1 and 2 but becomes negligible between rounds 2 and 3. In any case, the results are consistent with the choice of a cut-off s^*.

The results indicate that an additive model can provide a valid approximation of $\Delta G(x)$. S23G Fig also illustrates how the data are consistent between rounds: a refined analysis may infer a model that fits the data over all available rounds.

2. Experimental methods

Experimental methods are as in our previous work [3], except for target immobilization and sequencing data analysis as summarized below.

2.1. Phage production

Production of antibody-displaying phage was performed through infection of library cells (TG1 strain) with M13KO7 helper phage and growth at 30°C for 7 h in selective 2xYT medium containing 100 μg/mL ampicillin (Sigma-Aldrich, Saint-Louis, MO, USA) and 50 μg/mL kanamycin (Sigma-Aldrich, Saint-Louis, MO, USA). Cells were then centrifuged and the supernatant containing displaying phages was kept and stored

at 4°C overnight. All selections were performed on the day immediately following the phage production step.

2.2. Target immobilization

Target molecules were immobilized on streptavidin-coated magnetic beads (Dynabeads(R) M-280 Streptavidin) purchased from Invitrogen Life Technologies (Carlsbad, CA, USA). The DNA hairpin targets (DNA1 and DNA2) in fusion with a biotin at their $5'$ end were purchased from IDT (Leuven, Belgium) diluted in MilliQ water and stored at -20°C. The genes of protein targets (eGFP and mCherry, corresponding respectively to PDB IDs 2Y0G and 2H5Q) in fusion with a SBP tag were kindly provided by Sandrine Moutel (Institut Curie, Paris, France). They were produced in liquid T7 Express *E. Coli* cultures induced at $OD_{600} = 0.5$ with $300\,\mu$M Isopropyl β-D-1-thiogalactopyranoside (IPTG, Sigma-Aldrich, Saint-Louis, MO, USA) final and incubated overnight at 30°C. The proteins were harvested by threefold flash freezing in liquid nitrogen and quick thawing in a water bath at 42°C, followed by incubation with $50\,\mu$g/mL lyzozyme final and 2.5 U/mL DNase I final at 30°C for 15 minutes and centrifugation at $15,000\,$g and 4°C for 30 minutes. The supernatant was aliquoted in protein low-bind tubes (Protein LoBind, Eppendorf, Hamburg, Germany), flash frozen in liquid nitrogen and stored at -80°C until use.

Binding of target molecules to streptavidin-coated magnetic beads was performed in DNA low-bind tubes (DNA LoBind tubes, Eppendorf, Hamburg, Germany) for the DNA targets or protein low-bind tubes (Protein LoBind tubes, Eppendorf, Hamburg, Germany) for the protein targets. Beads and targets were incubated in 0.5x PBS for protein targets and 0.9x PBS for DNA targets at ambient temperature on a rocker for 15 min, followed by removal of all liquid and 3 washing steps: addition of $500\,\mu$L washing solution, vortexing, separation of beads using a magnet and removal of all liquid. Finally, the beads were stored in washing buffer at 4°C for use on the following day. Bw1X buffer (1 M NaCl, 5 mM Trizma at pH = 7.4, 0.5 mM EDTA) was used as washing buffer for DNA targets (to screen electrostatic interactions), 1x PBS with 0.1 % Tween20 for protein targets (to screen hydrophobic interactions). The same procedure was followed for negative/null selection tubes, with MilliQ water instead of target solutions.

Successful immobilization of protein targets was confirmed by fluorescence measurements of treated beads against untreated and MilliQ water-treated beads as negative controls.

2.3. Phage display selection

The selection protocol is as previously published in [3]. The washing buffer was removed from the target-covered beads. Then, 1 mL of culture supernatant from the phage production step containing $\approx 10^{11}$ phages was added to the negative selection tube (containing no targets) and incubated for 90 minutes at ambient temperature, shaking. The beads were separated by a magnet and the liquid was transferred to the positive selection tube (containing the targets) and incubated for 90 minutes at room temperature, shaking. Finally, all liquid containing unbound phage was removed and the beads were subjected to a 10-fold washing using 10 mL of 1x PBS with 0.1 % Tween20. Bound phage were eluted from beads with 1.4 % triethylamine (Sigma-Aldrich, Saint-Louis, MO, USA) in MilliQ water and used for infection of fresh exponential TG1 cells to obtain the selected library.

2.4. Illumina sequencing

Glycerol stocks of library cells at relevant selection cycles were defrosted and plasmids were extracted using purification kits from Macherey-Nagel (Düren, Germany). We used glycerol stocks obtained from infecting fresh cells with output phages from the selection steps (which represent the selected libraries at various cycles $c = 1, 2, \dots$) and glycerol stocks obtained from infecting fresh cells with input phages from the first selection round (which represent the initial library $c = 0$). No liquid culture was performed prior to plasmid extraction to avoid potential additional biases from growing an overnight culture beforehand. Resulting plasmids were used as input for Illumina sequencing preparation PCR: a first reaction using primer sequences common to all three libraries downstream CDR_3 (`GCTCGAGACGGTAACCAGG`, forward) and halfway inside V_H (`ACAACCCGTCTCTTAAGTCTCGT`, reverse) added random barcodes of length 5 nt to discriminate between neighboring clusters. A second reaction added P5 and P7 indices to identify library, target and selection round corresponding to each cluster, as well as the adapter for the sequencing procedure. Illumina sequencing and demultiplexing were performed at I2BC, Gif-sur-Yvette, France. Due to the particular treatment of the initial library ($c = 0$), enrichments from a first round of selection are between the output and input of the selection step, while those from later rounds of selection are between the output of consecutive selection steps. As a consequence, the amplification bias which results from the phage production step is eliminated in enrichments computed from a first round of selection (as was done in mini-library selections).

2.5. Data preprocessing

The Illumina sequencing yields for each sample (i.e., each library, target and selection round) between 10^5 and 5.10^6 sequencing clusters. The data files contain the entirely overlapping forward and reverse reads for all clusters of a given sample. Each cluster was accepted or discarded based on the following procedure: Both the forward and reverse reads were screened for the presence of the primer sequences (up to 4 nt mismatch accepted for each) and cut to keep only the part between the primers (including the primers). Either one was discarded if the primer search was unsuccessful. We then checked if the remaining forward and/or reverse sequence fragments have the expected length of 170 nt, corresponding to the region of interest. If only one direction had the expected length, only this direction was kept and the other one was discarded. If both directions did not have expected length, the complete cluster was discarded. Finally, if both reads had expected length, a consensus sequence was generated by taking on each position with disagreement between both reads the nucleotide measured with highest quality read. A final check was performed for (i) a sufficient average quality read over the whole region of interest ($\langle Q \rangle \geq 59$) and (ii) the restriction sites immediately up- and downstream CDR3 (`TGTGCGCGC` and `TTCGACTAC`) are located at their expected positions (108-116 and 129-137 in reverse direction; up to 4 nt mismatch accepted for each). The cluster was discarded if either of these two criteria was not fulfilled.

After completion of this procedure, (i) the framework (Germ, Lim or Bnab) and (ii) the CDR3 sequence for all remaining sequencing reads in the full-library experiments were identified. Step (i) was performed by measuring the Hamming distance of the visible library-specific framework part upstream the CDR3 of the read (of length 116 nt) to all three framework reference sequences. The read was assigned to the nearest framework if the Hamming distance to the nearest framework was ≤ 7 nt *and* the difference in Hamming distance to the nearest and next-nearest frameworks was ≥ 3 nt. For step (ii), the CDR3 sequence was simply extracted from the read for the full-library experiments. For the selections with reduced diversity a

similar procedure as for the framework part was applied: the measured CDR3 sequence was assigned to the nearest among ~ 20 reference sequences if the Hamming distance was ≤ 3 nt and the difference in Hamming distance between nearest and next-nearest was ≥ 1 nt. After assessment of the sequence identity of all clusters in a dataset, the CDR3 sequences were translated into amino acids and the number of occurrences of each clone (determined by its framework and its CDR3 sequence) was counted.

The nucleotide sequences of the visible framework parts upstream the CDR3 of all three libraries as well as the Hamming distances d_H between the pairs is as follows:

Germ:

`ACAACCCGTCTCTTAAGTCTCGTGTTACCATCTCTGTTGACACCTCTAAAAACCAGTT...`

`CTCTCTGAAACTGTCTTCTGTTACTGCGGCGGACACTGCGGTTTACTACTGTGCGCGC`

Lim:

`ACAACCCGTCTCTTAAGTCTCGTGTTACCATCTCTATCGACACCTCTAAAAACCACTT...`

`CTCTCTGCGTCTGATCTCTGTTACTGCGGCGGACACTGCGGTTTACCACTGTGCGCGC`

Bnab:

`ACAACCCGTCTCTTAAGTCTCGTCTGACCCTGGCGCTGGACACCCCGAAAAACCTGGT...`

`TTTCCTGAAACTGAACTCTGTTACTGCGGCGGACACCGCGACCTACTACTGTGCGCGC`

$d_H(\text{Germ}, \text{Lim}) = 10$ nt, $d_H(\text{Lim}, \text{Bnab}) = 25$ nt and $d_H(\text{Germ}, \text{Bnab}) = 22$ nt.

For the mixed full-library selections, final data files contain three columns: 1) framework identity ('`germ`' for Germline, '`lmtd`' for Limited, '`bnAb`' for Bnab, '`????`' if framework inference failed), 2) CDR3 identity given by the sequence of 4 amino acids or the sequence of 12 nucleotides or by '`????`' if the CDR3 readout failed, 3) number of occurrences in the dataset. The preprocessed data from the experiments reported in this paper is made available in this format.

We checked that the results are unaffected by the choice of the parameters in the preprocessing procedure described here.

References

[1] M. Djordjevic, A. M. Sengupta, Quantitative modeling and data analysis of selex experiments, Physical biology 3 (1) (2005) 13.

[2] C. Rastogi, H. T. Rube, J. F. Kribelbauer, J. Crocker, R. E. Loker, G. D. Martini, O. Laptenko, W. A. Freed-Pastor, C. Prives, D. L. Stern, R. S. Mann, H. J. Bussemaker, Accurate and sensitive quantification of protein-DNA binding affinity., Proc. Natl. Acad. Sci. U.S.A. 115 (16) (2018) E3692–E3701.

[3] S. Boyer, D. Biswas, A. Kumar Soshee, N. Scaramozzino, C. Nizak, O. Rivoire, Hierarchy and extremes in selections from pools of randomized proteins., Proc. Natl. Acad. Sci. U.S.A. 113 (13) (2016) 3482–3487.

[4] S. Coles, J. Bawa, L. Trenner, P. Dorazio, An introduction to statistical modeling of extreme values, Springer, 2001.

[5] R. Perline, Strong, weak and false inverse power laws, Statistical Science 20 (1) (2005) 66–88.

[6] E. J. Gümbel, Statistics of extremes, Columbia Univ. Press, 1958.

[7] T. M. Cover, J. A. Thomas, Elements of information theory, John Wiley & Sons, 2012.

[8] S. A. Frank, Natural selection. V. How to read the fundamental equations of evolutionary change in terms of information theory., Journal of evolutionary biology 25 (12) (2012) 2377–2396.

[9] M. H. Huntley, A. Murugan, M. P. Brenner, Information capacity of specific interactions., Proc. Natl. Acad. Sci. U.S.A. 113 (21) (2016) 5841–5846.

[10] J. Otwinowski, Biophysical inference of epistasis and the effects of mutations on protein stability and function. Molecular biology and evolution., 35 (10) (2018) 2345–2354.

[11] S. F. Levy, J. R. Blundell, S. Venkataram, D. A. Petrov, D.S. Fisher, , G. Sherlock, Quantitative evolutionary dynamics using high-resolution lineage tracking. Nature 519(7542) (2015) 181–186.
