# Supplementary figures and images for: Parameters and determinants of responses to selection in antibody libraries
Source: PLoS Comput Biol. 2021 Mar 25;17(3):e1008751. doi: 10.1371/journal.pcbi.1008751 (PMC7993935; doi:10.1371/journal.pcbi.1008751)

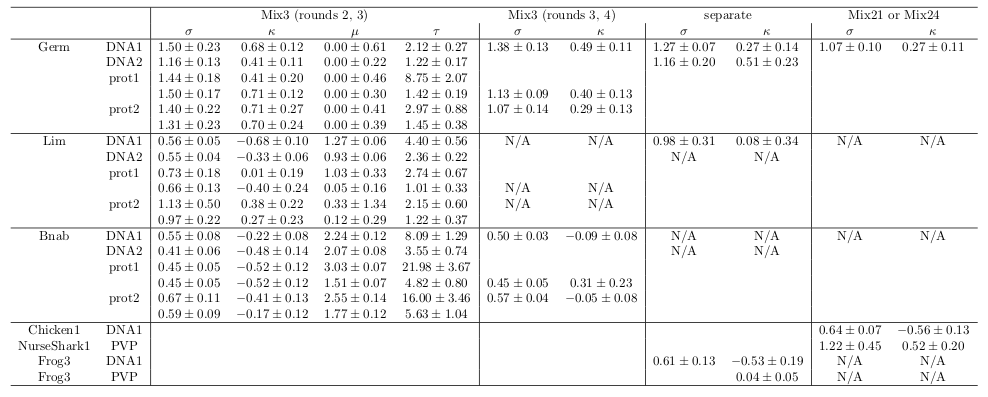

Supplement: S1 Table — N/A indicates that the data was insufficient to make a meaningful fit. For enrichments against the protein targets between rounds c = 2 and c + 1 = 3, values are given for two independent replica of the experiment. The given uncertainties correspond to a single standard deviation around the maximum likelihood estimate as given by the Cramér-Rao bound. In the case of Frog3 against DNA1, and only in this case, the value of κ differs from the one reported in our previous work [16] for reasons explained in S15 Fig. (TIF) [file pcbi.1008751.s002.tif]

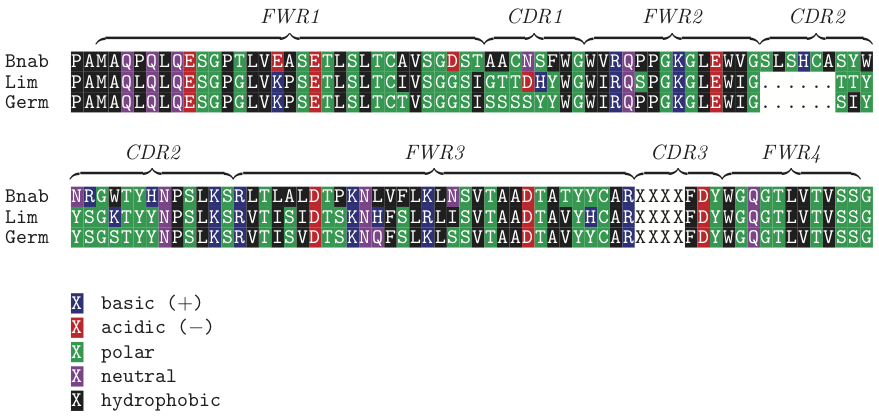

Supplement: S1 Fig — The 4 randomized positions correspond to the part of the CDR3 indicated by XXXX. (TIF) [file pcbi.1008751.s003.tif]

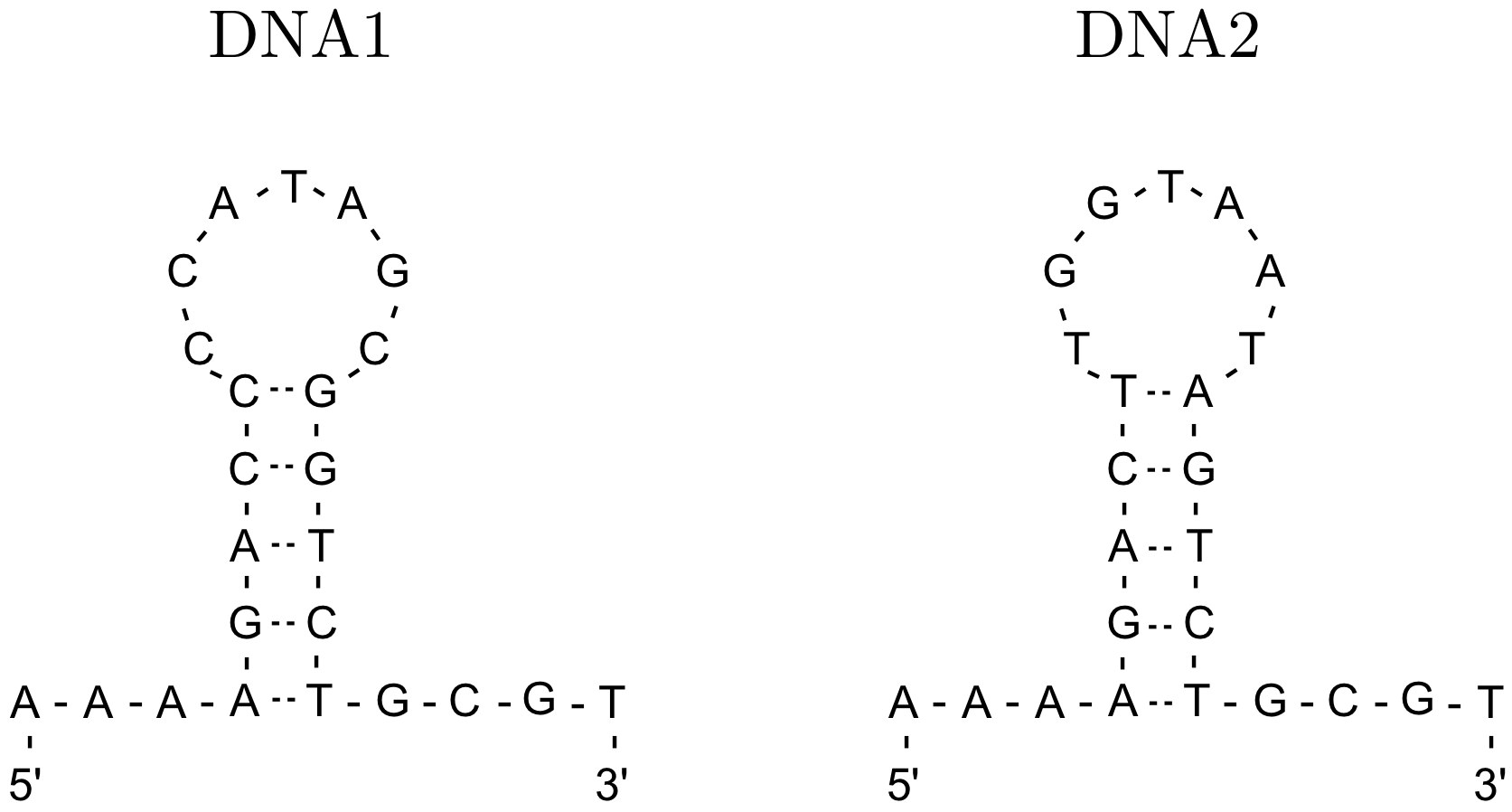

Supplement: S2 Fig — The targets display a hairpin structure at room temperature. They share a common stem sequence but the sequence of their loop differ. A biotin is placed at the 5’ ends to allow for immobilization on streptavidin-coated magnetic beads. (TIF) [file pcbi.1008751.s004.tif]

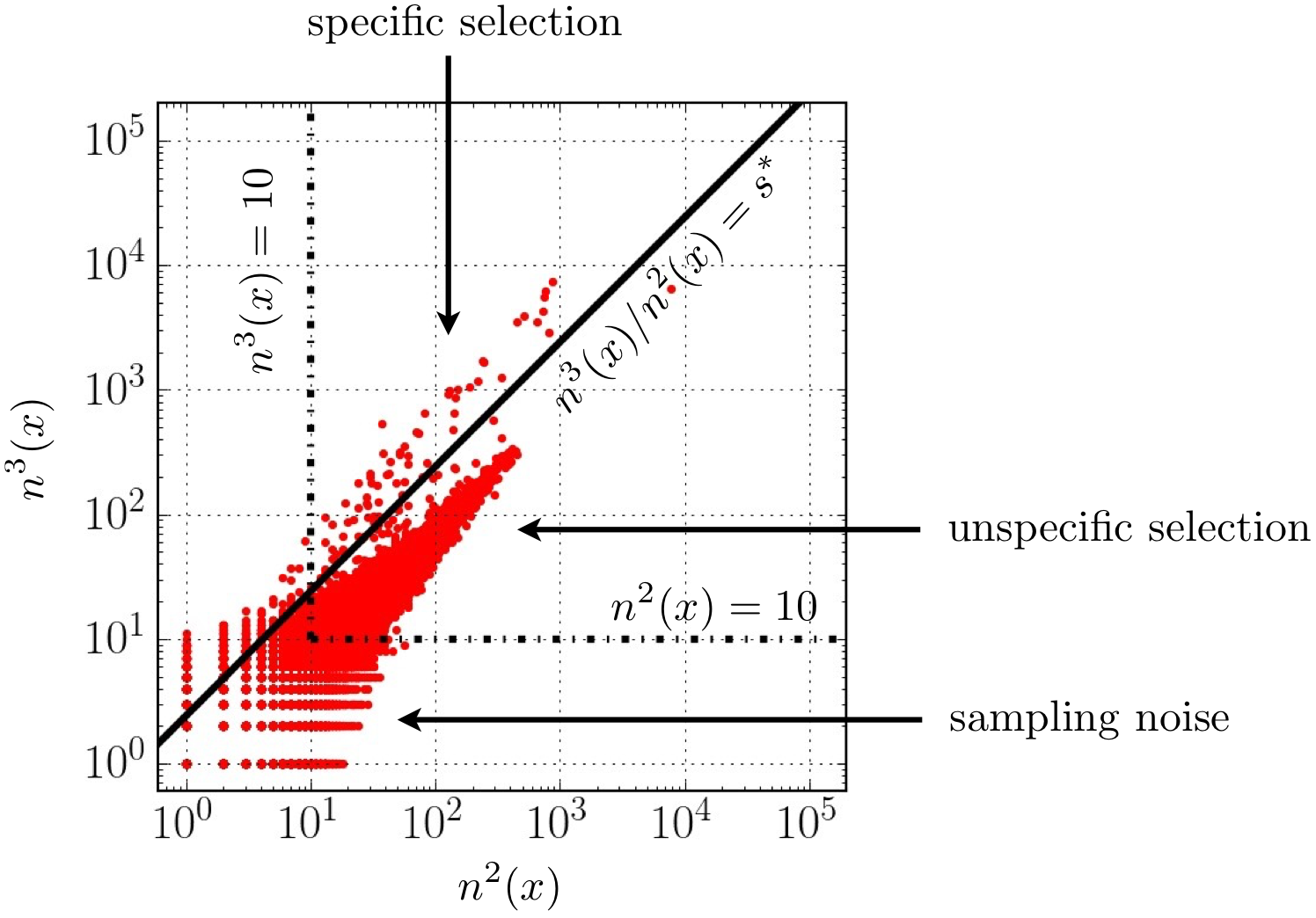

Supplement: S3 Fig — The number n3(x) of counts in the sequencing data at round c = 3 is plotted against the number n2(x) of counts at round c − 1 = 2 for a selection of the Bnab library mixed with the two other libraries against the DNA1 target. An accumulation of sequences with similar enrichments is observed along the diagonal, with larger variance for smaller values as expected from an increased sampling noise. This is interpreted as arising from unspecific selection, e.g., through unspecific binding, associated with an enrichment sus independent of the sequence. We define a cut-off s* such that sequences x with s = n3(x)/n2(x) ≥ s* cannot be attributed to unspecific selection. In addition, we restrict to sequences x with n2(x) ≥ 10 and n3(x) ≥ 10, as represented by the vertical and horizontal lines, to ensure that the inferred enrichments are not dominated by sampling noise. (TIF) [file pcbi.1008751.s005.tif]

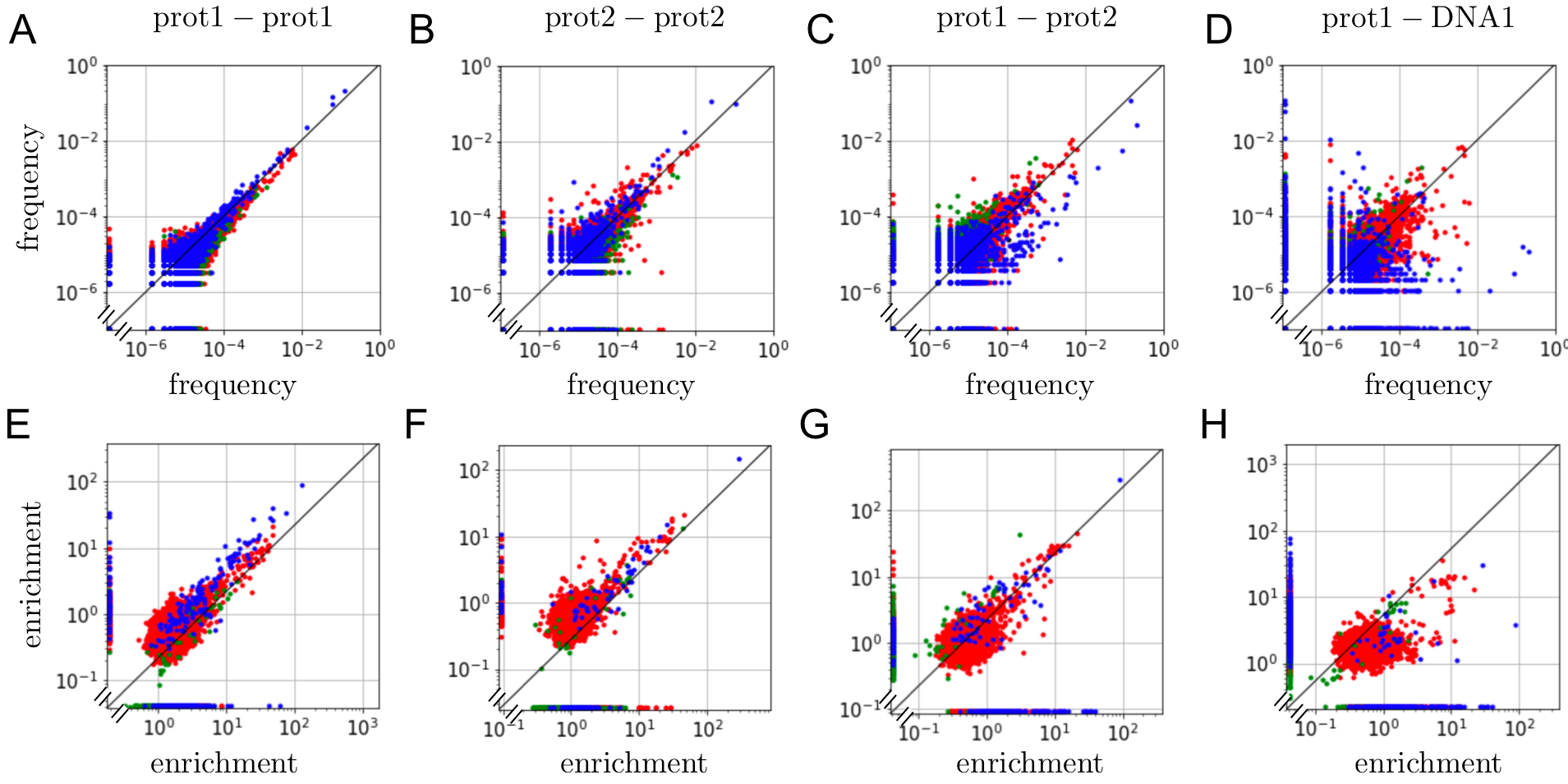

Supplement: S4 Fig — A. Comparison of the frequencies f3(x) = n3(x)/∑x′ n3(x′) computed after the third cycle (c = 3) between two independent replicate experiments where a mixture of the Germ (in blue), Lim (in green) and Bnab (in red) libraries is selected against the protein target prot1. Due to stochastic sampling, some sequences x are well represented in one experiment (n3(x) ≥ 10) but not in the other; they are represented by the points along the two axes. As expected, the frequencies of the most prevalent sequences are the most reproducible. B. As in A but for protein target prot2. C. Comparing an experiment with prot1 as target with another with prot2 as target: common sequences are enriched in the two cases, although with not exactly the same frequencies. D. Comparing an experiment with prot1 as target with another with DNA1 as target, showing that different sequences are enriched in each case. In particular, the most frequent sequences when selecting against one target are absent in the third round when selecting against the other (points along the axes). E,F,G,H. Comparison of enrichments s(x) calculated from the frequencies between the second and third rounds as s(x) = λn3(x)/n2(x). Points along the axes correspond to sequences for which the enrichment could be estimated only for one of the two experiments. We verify that in cases E,F,G where the targets are similar the same top enrichments are recovered (up to a multiplicative constant corresponding to a shift in log-log plots). Beyond stochastic effects, reproducibility is mainly limited by the differences in the production of the targets, as shown in S12 Fig. (TIF) [file pcbi.1008751.s006.tif]

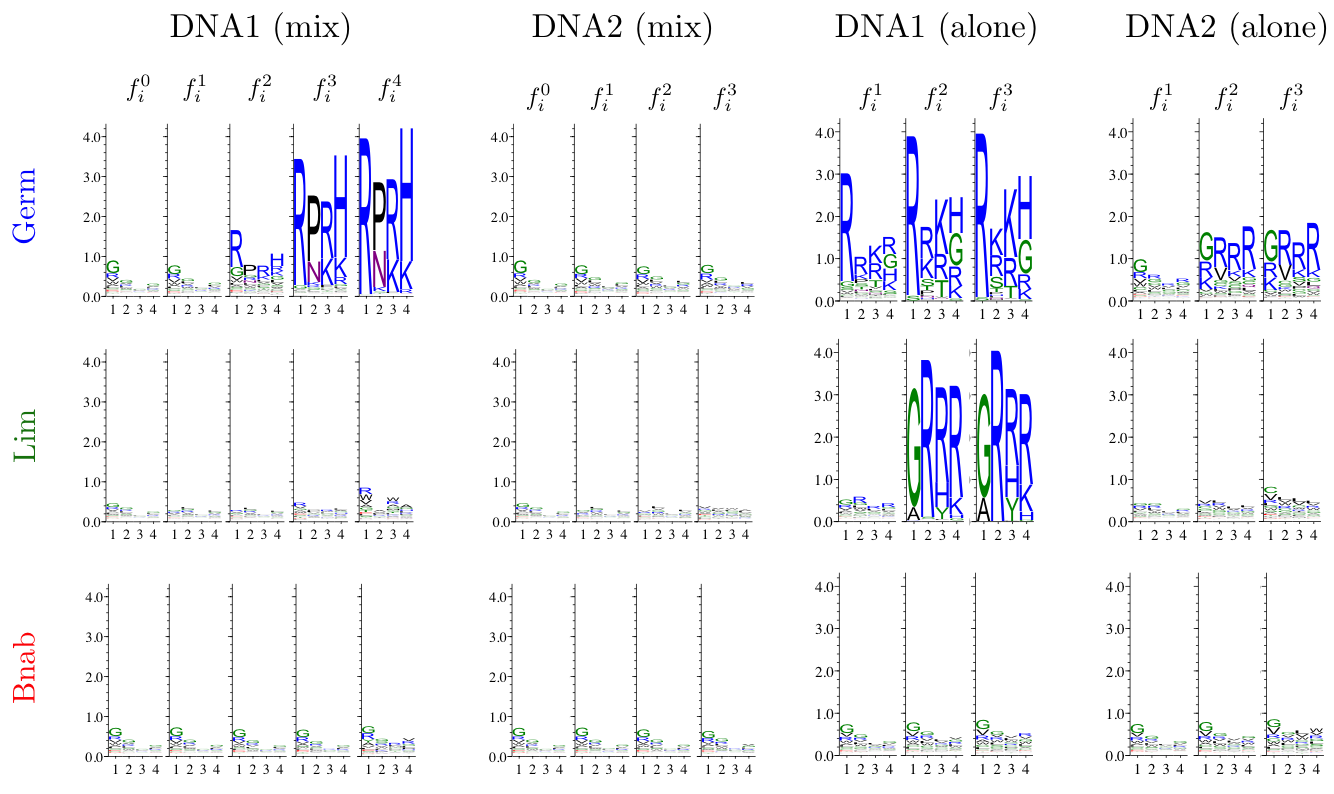

Supplement: S5 Fig — The sequences logos represent the frequencies fic(a) of amino acids at each successive cycle c = 0, 1, 2, 3, 4. (TIF) [file pcbi.1008751.s007.tif]

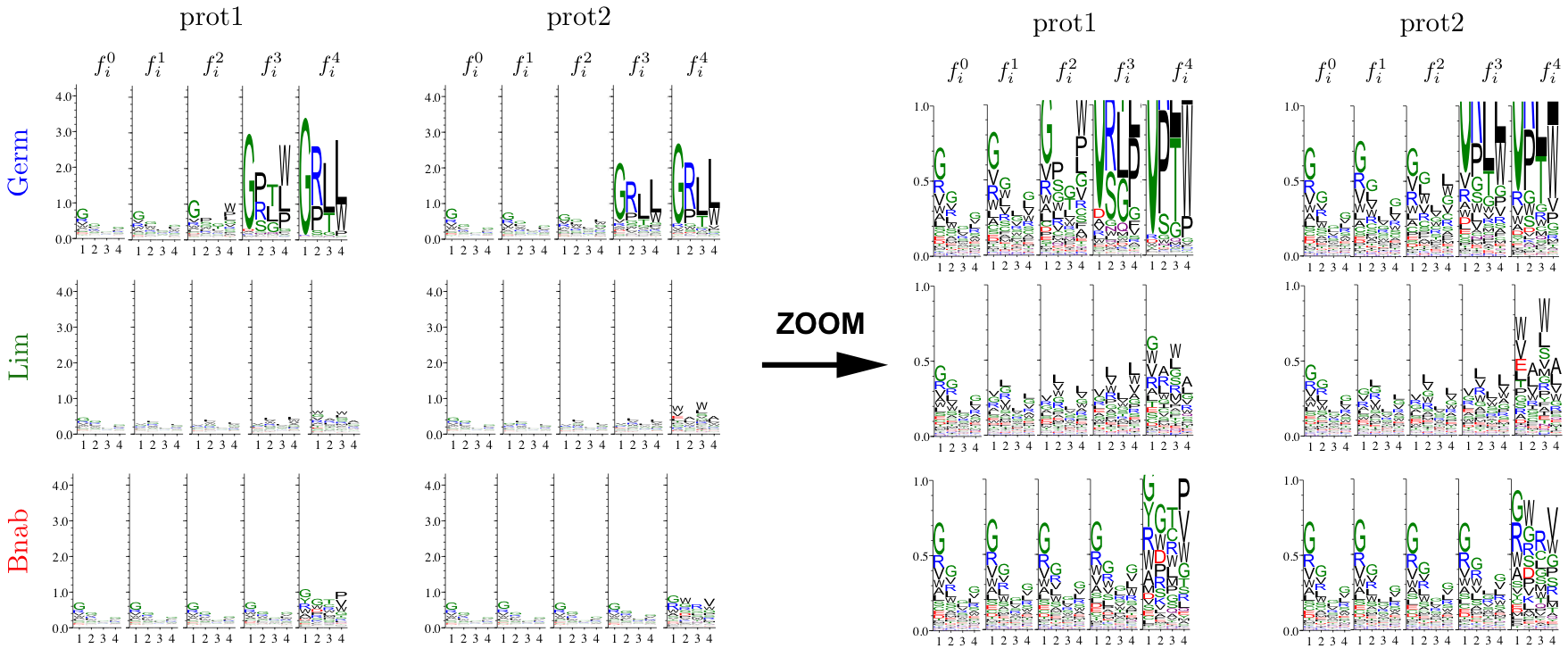

Supplement: S6 Fig — The sequences logos represent the frequencies fic(a) of amino acids at each successive cycle c = 0, 1, 2, 3, 4. The data is presented at two different scales for better readability. (TIF) [file pcbi.1008751.s008.tif]

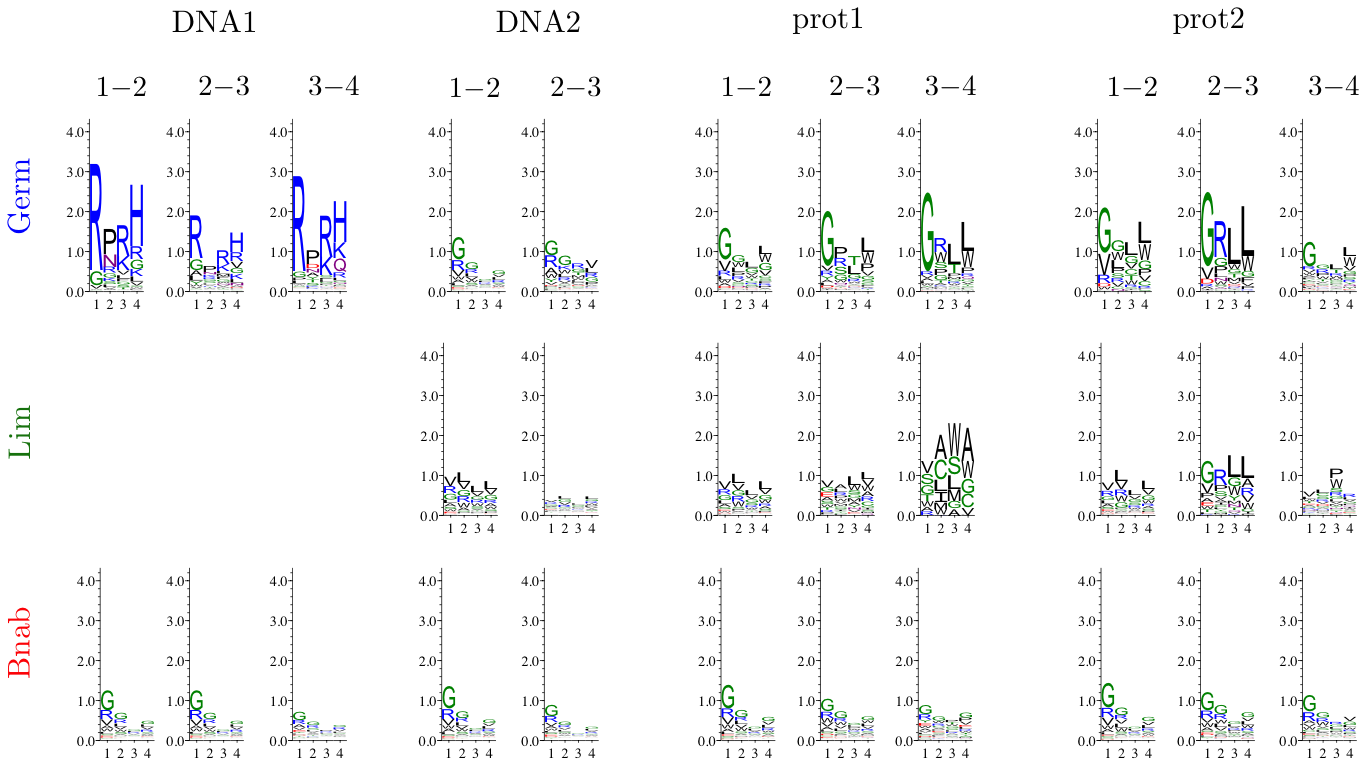

Supplement: S7 Fig — The differences between rounds reflect sampling fluctuations. (TIF) [file pcbi.1008751.s009.tif]

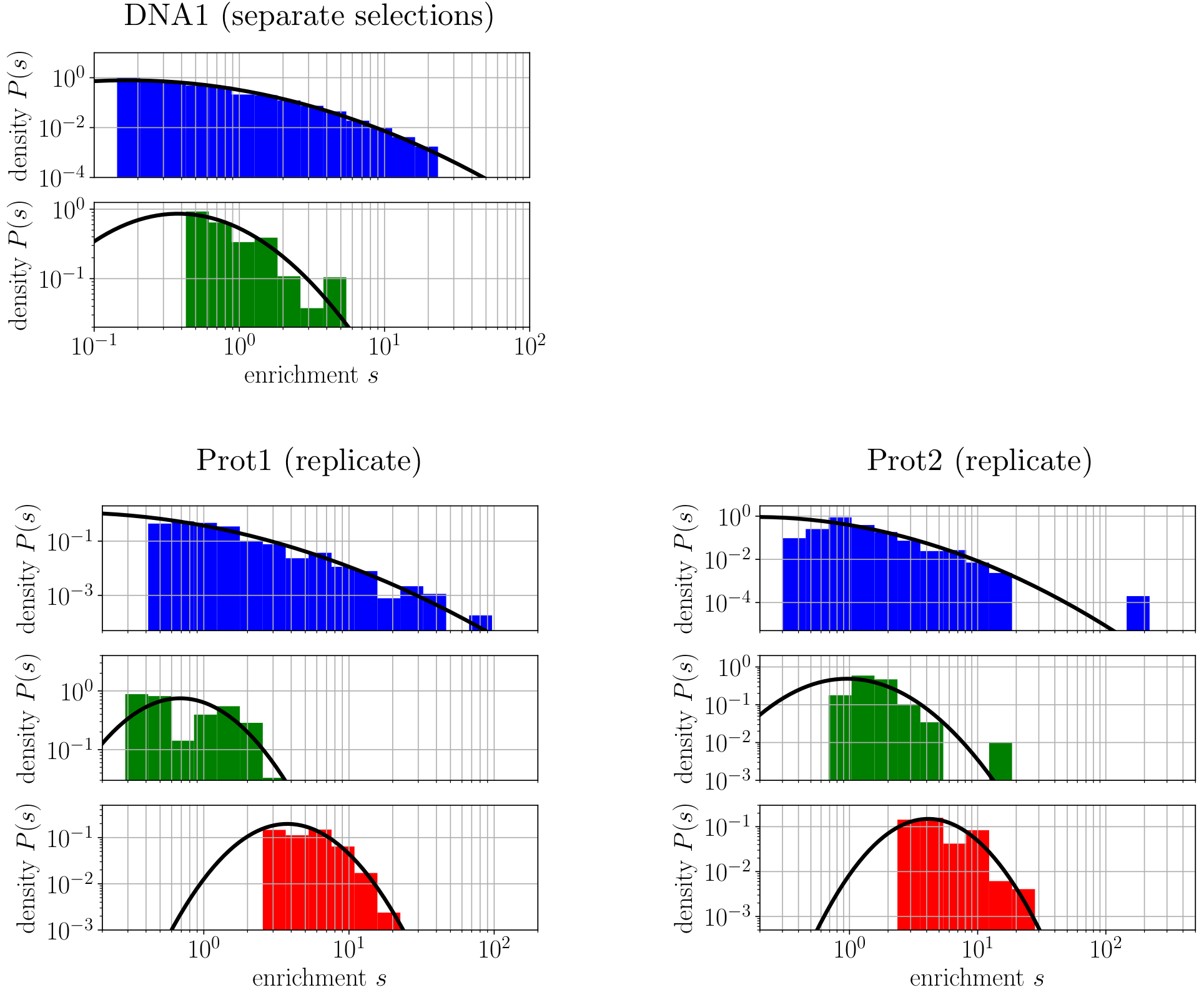

Supplement: S8 Fig — Top: separate selections of the Germ and Lim libraries against the DNA1 target. Here enrichments are computed between rounds 1 and 2. Note that the μ cannot be compared and is fixed to μ = 0 in both cases. These experiments complement those of Fig 1 where the libraries are selected together, which does not leave sufficient data for the analysis of the Lim library against the DNA1 target. Bottom: analyses of replicate experiments where the three libraries are jointly selected against the two protein targets, as in the bottom panels of Fig 1. (TIF) [file pcbi.1008751.s010.tif]

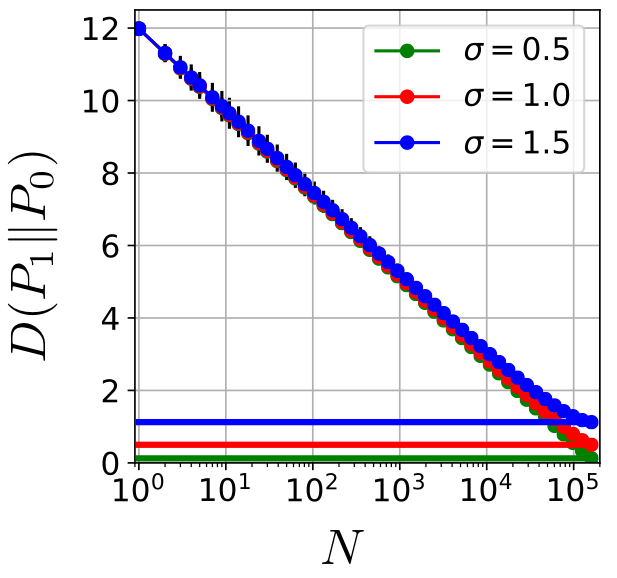

Supplement: S9 Fig — 105 values were drawn from a log-normal distribution with parameters μ = 0 and σ = 0.5 (green), 1 (red) and 1.5 (blue). The relative entropy D(P1‖P0) was then estimated using a random subsample of size N. For any N < 105, this leads to an overestimation of D(P1‖P0) whose actual value σ2/2 (see Eq. 14 in S1 Text) is represented by the horizontal lines at the bottom. (TIF) [file pcbi.1008751.s011.tif]

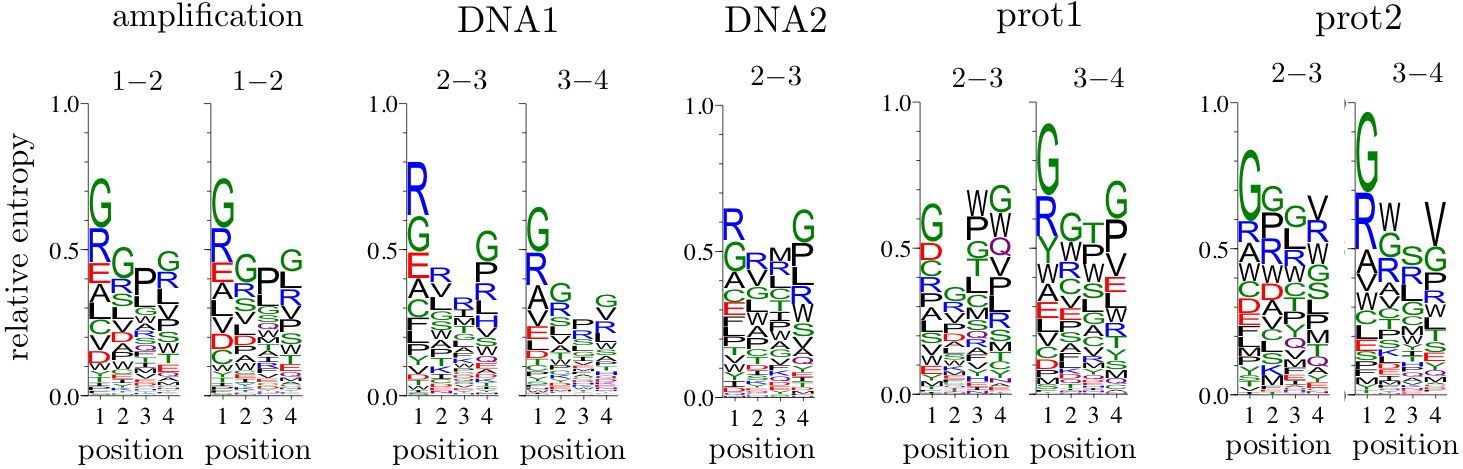

Supplement: S10 Fig — The enrichments are computed between the first and second cycles (1-2) or between the third and fourth cycles (3-4); for amplification only, the results of two replicate experiments are shown. The sequence logos of enrichments calculated between rounds 2 and 3 are the same as those shown in Fig 2 (Bnab library), except for the scale along the y-axis. All sequences logos share common patterns reflecting a common contribution from amplification biases. Sequence logos against the protein targets show, however, an enrichment for tryptophane (symbol W) that is not observed when selection involves amplification only. Selections of the Bnab library thus have a target-dependent contribution from binding affinity of similar order of magnitude as a common target-independent contribution from amplification biases. (TIF) [file pcbi.1008751.s012.tif]

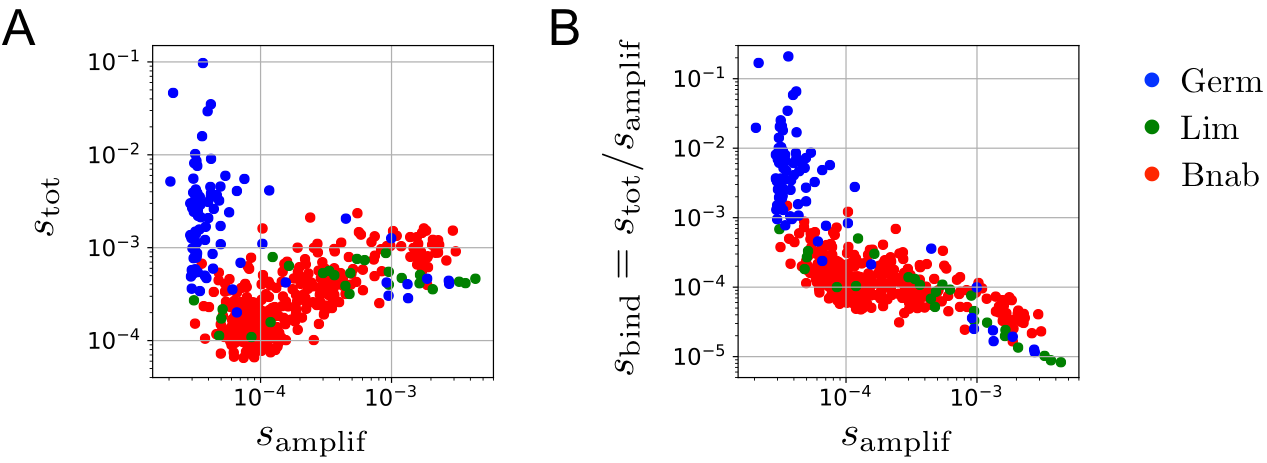

Supplement: S11 Fig — A separate experiment without any selection for binding was performed to estimate the difference of enrichments arising from the amplification step alone. A. The resulting samplif is here compared to the enrichments stot from an experiment including a selection for binding. The sequences with top stot, which all belong to the Germ library (in blue), are among the sequences with lowest samplif, which indicate that they are selected for binding with no contribution from the amplification bias. On the other hand, the sequences with top stot from the Lim and Bnab libraries (respectively in green and red), have also top samplif, which indicate a significant contribution from amplification biases. B. The ratio stot/samplif represents the contribution to enrichment of binding alone. The two selective pressures, binding and amplification, appear here to be orthogonal. (TIF) [file pcbi.1008751.s013.tif]

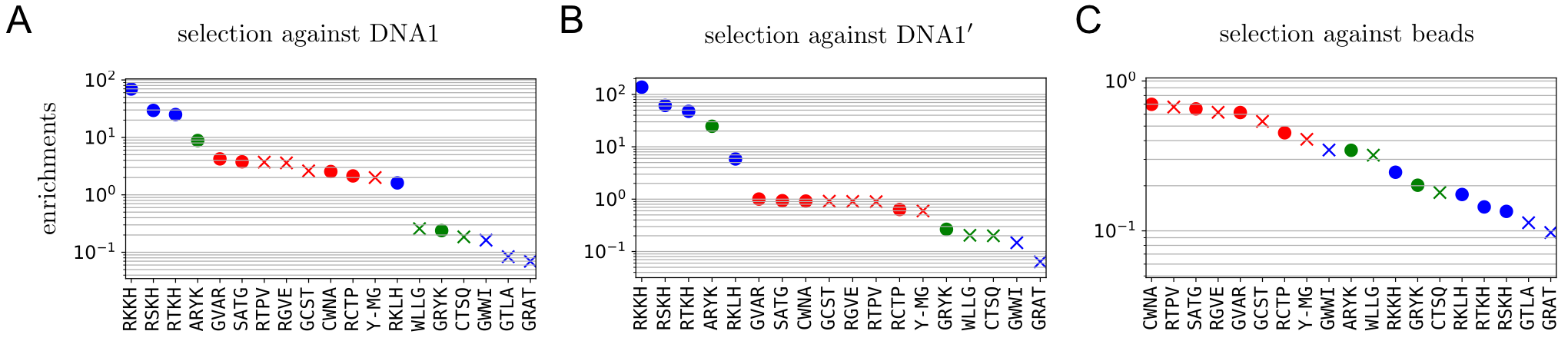

Supplement: S12 Fig — A. Enrichments of top and random sequences from the three libraries, Germ (in blue), Lim (in green) and Bnab (in red), against DNA1. B. Results from a replicate experiment using a different stock of beads, showing that the enrichments are reproduced except for the Bnab sequences (in red), which have a systematically higher enrichment. C. Similar to A, but when selecting for binding to the beads in absence of the DNA1 target. The top enrichments are from the Bnab sequences (in red), indicating that they bind to the beads, a finding consistent with the discrepancy between A and B. Here, the differences in enrichments are also coming from differences of enrichment during amplification (S11 Fig). Consistent with S11 Fig, the top Germ sequences (blue dots) have in absence of the DNA1 target the worst enrichments. (TIF) [file pcbi.1008751.s014.tif]

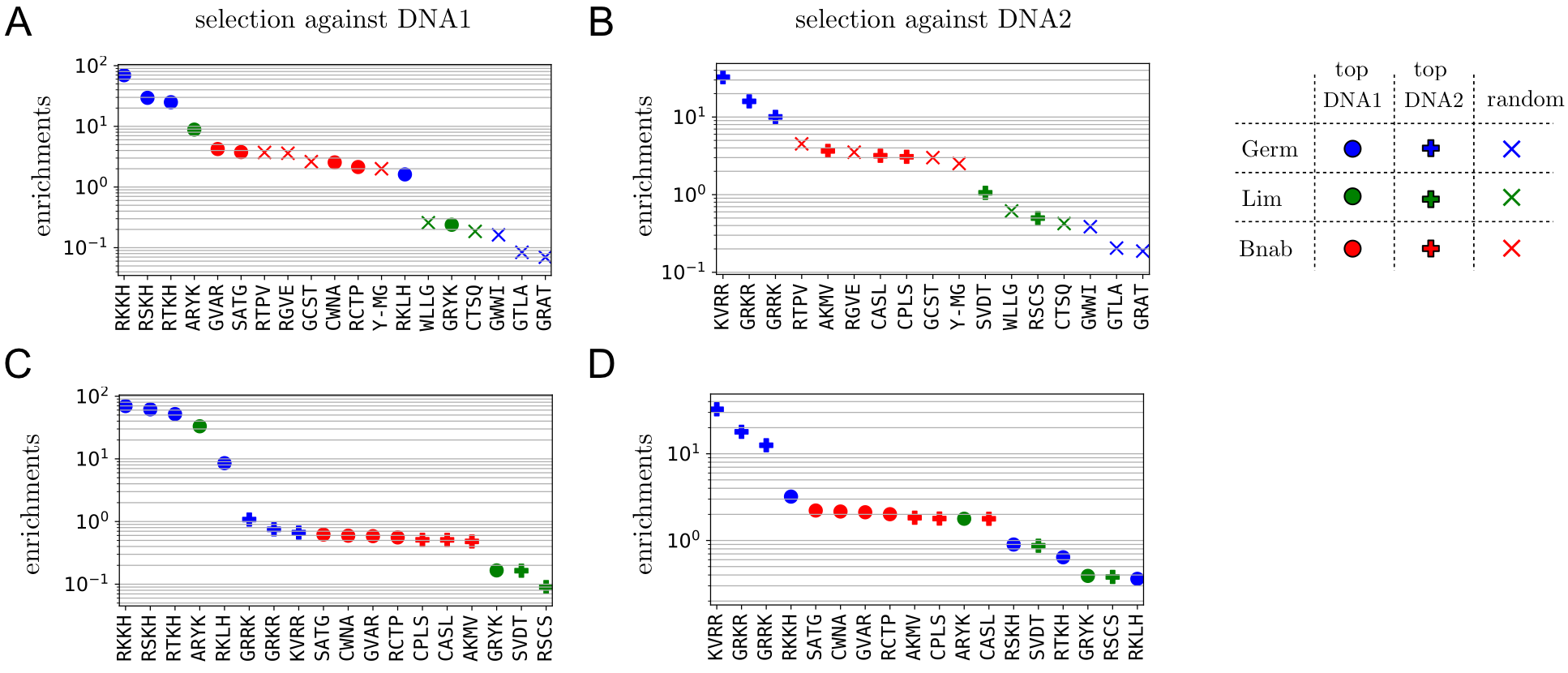

Supplement: S13 Fig — A,C. Selection against the DNA1 target (same as in S12 Fig). B,D. Selection against the DNA2 target. The results confirm that some sequences from the Germ and Lim libraries bind specifically to the DNA1 target (blue dots and one of the green dots) and some sequences from the Germ library to the DNA2 target (blue crosses). (TIF) [file pcbi.1008751.s015.tif]

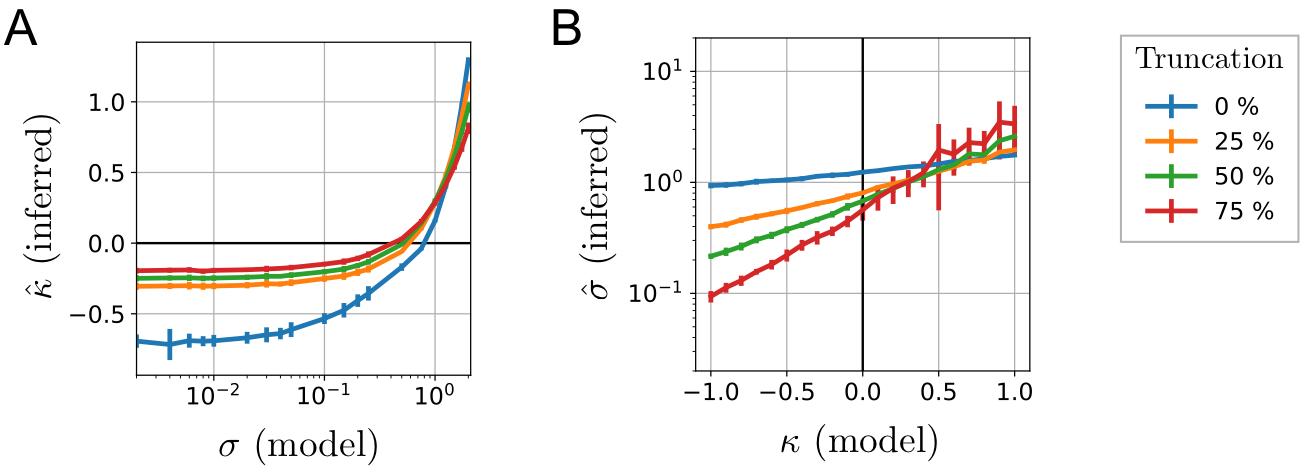

Supplement: S14 Fig — A. N = 104 values were drawn from a log-normal distribution with parameters μ = 0 and varying σ (x-axis). The largest 25, 50, 75, 100% of these values (i.e., 75, 50, 25, 0% truncation) were fitted to a Pareto model with parameters κ and τ. The plot shows the estimation κ^ as a function of σ. Averages and standard deviations are taken over 25 independent realizations of the numerical experiment. It shows that limited sampling may cause a κ^<0 to be inferred from values drawn from a log-normal distribution when σ is small, here σ < 0.5. B. Inverse simulation: A truncated log-normal model is fitted to the largest 25, 50, 75, 100% among 500 values (i.e., 75, 50, 25, 0% truncation) drawn from a Pareto model with parameters τ = 0.115, s* = 0.001 and varying κ (x-axis). The black dotted line in Fig 4 corresponds to the 25% truncation. (TIF) [file pcbi.1008751.s016.tif]

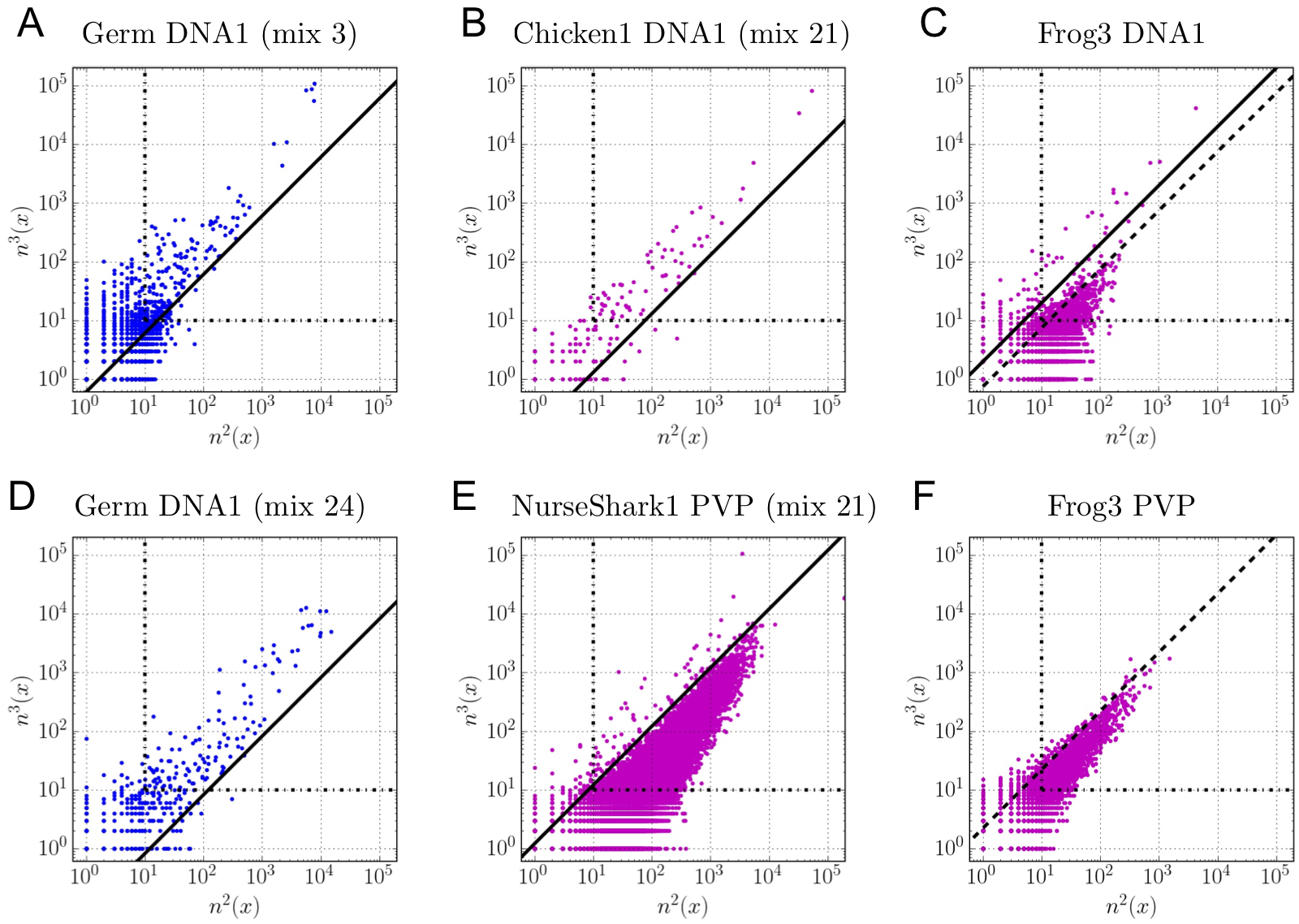

Supplement: S15 Fig — As in S3 Fig, the definition is based on a comparison between counts at the 2nd and 3rd cycles. The horizontal and vertical lines correspond to the criteria n2(x) ≥ 10 and n3(x) ≥ 10. The plain oblique line corresponds to the definition of s* in this work. In the case of the selection of the Frog3 library against the DNA1 target, it differs from the value of s* used in our previous work [16] (dotted oblique line) which failed to discard many enrichments coming from unspecific binding. In the case of the selection of the Frog3 library against the PVP target, all measured enrichments may be attributed to unspecific binding and we are therefore not including the inferred values of σ and κ in Fig 4. (TIF) [file pcbi.1008751.s017.tif]

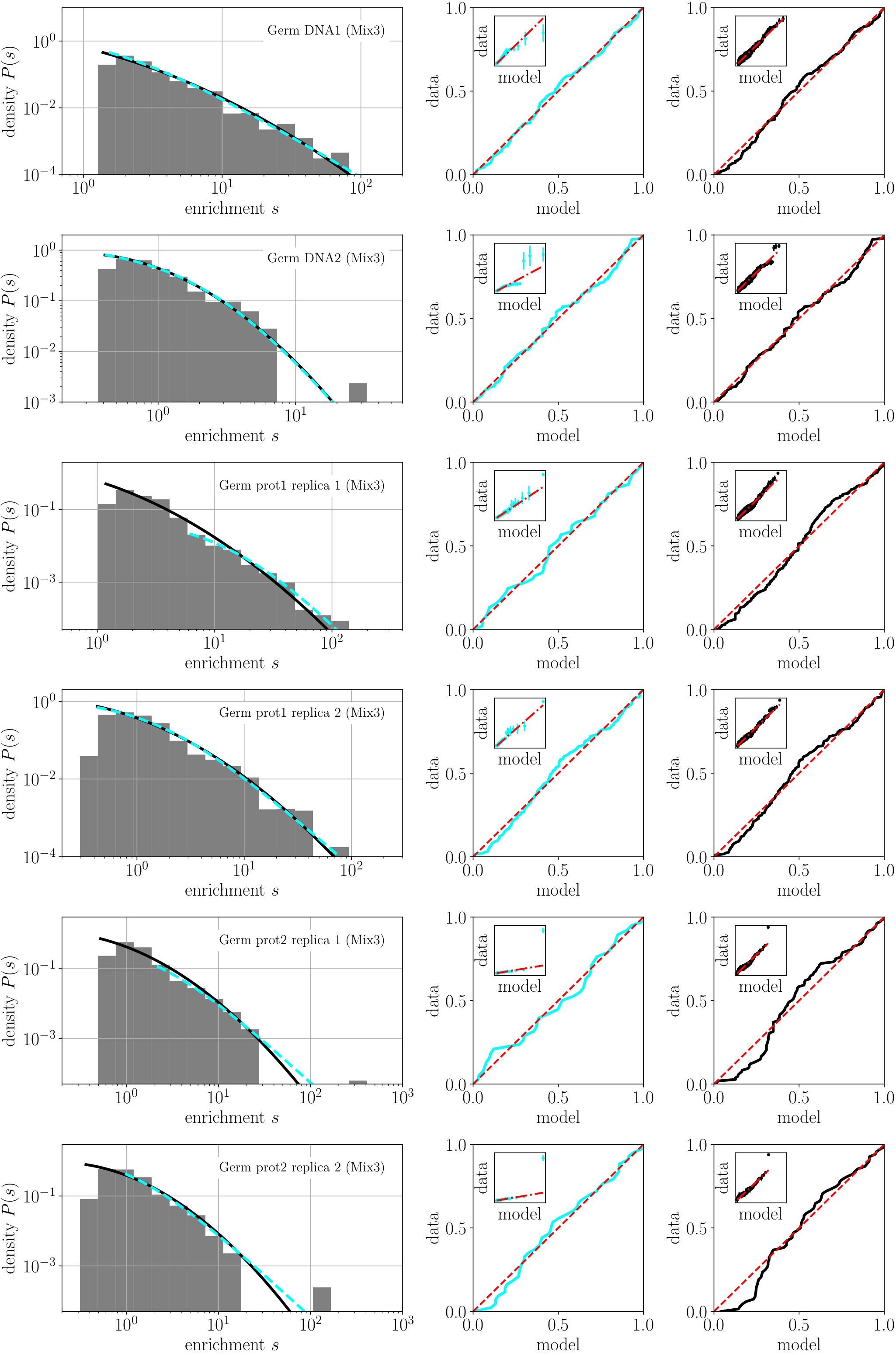

Supplement: S16 Fig — The different graphs correspond to selections against different targets. For the protein targets prot1 and prot2, results from two replicate experiments are presented. All enrichments are computed by comparing the frequencies at the 2nd and 3rd cycle. The graphs on the right show the P-P and Q-Q (inset) plots for each fit. Perfect fits would correspond to the red dotted lines. (TIF) [file pcbi.1008751.s018.tif]

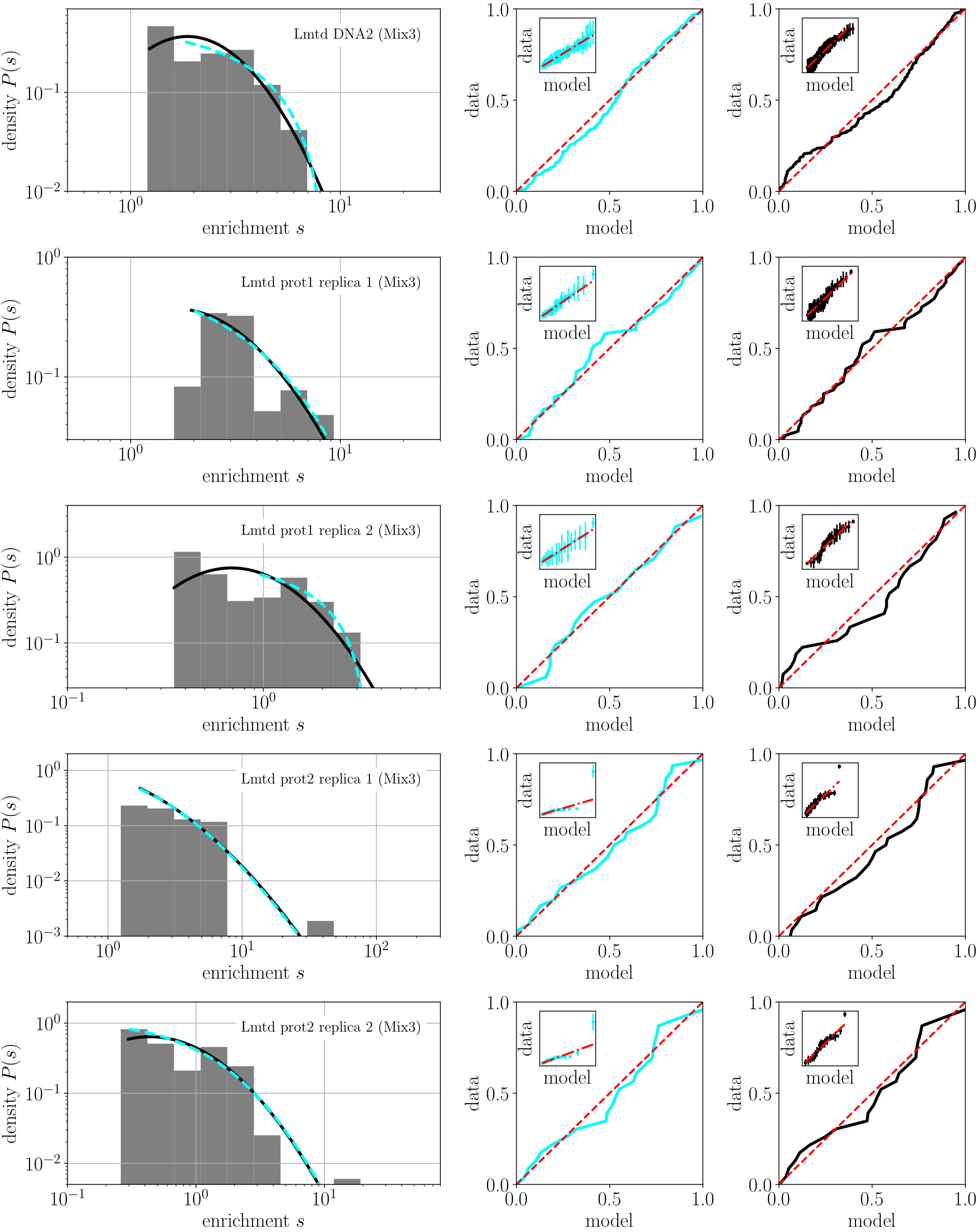

Supplement: S17 Fig — (TIF) [file pcbi.1008751.s019.tif]

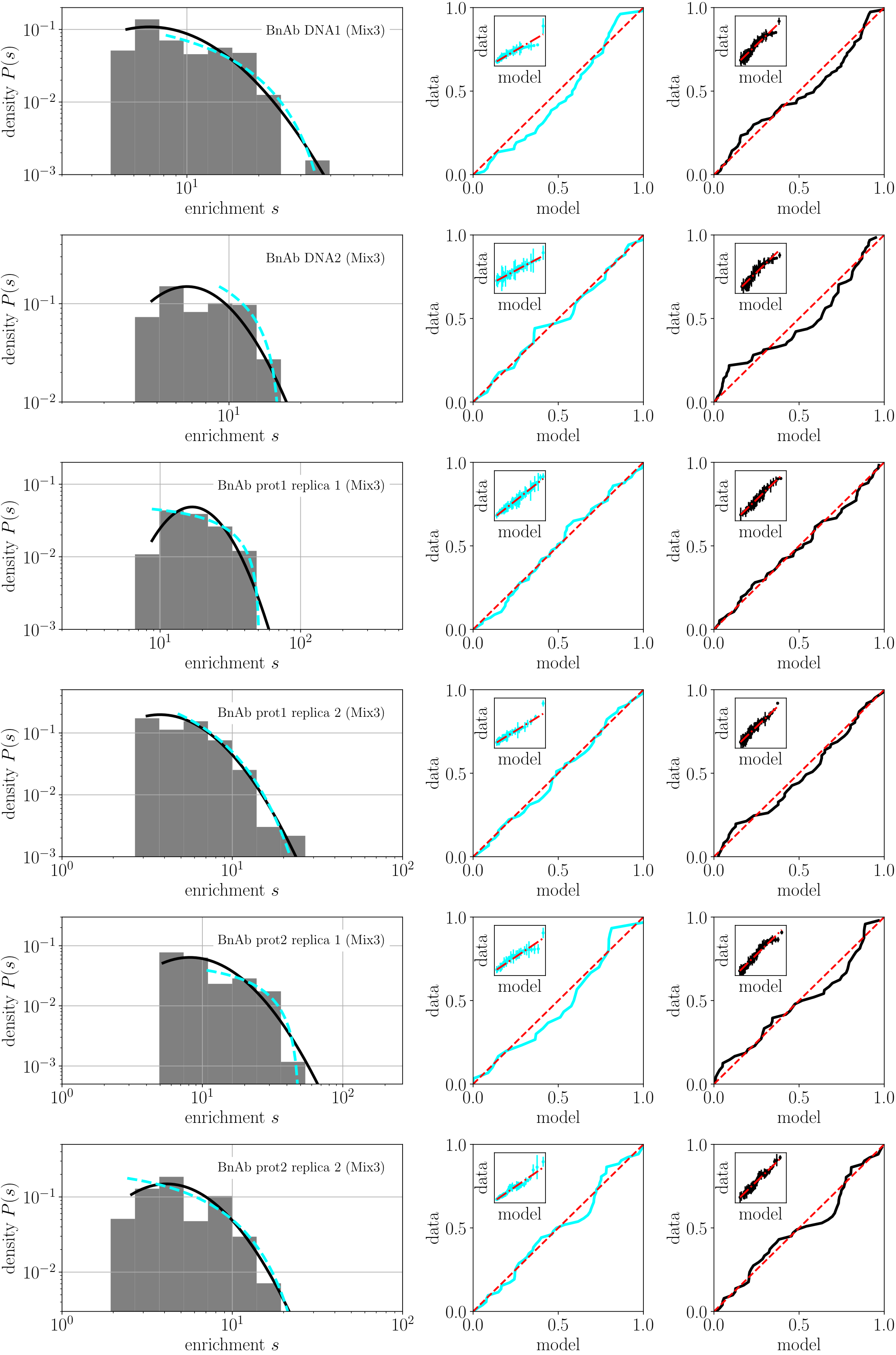

Supplement: S18 Fig — (TIF) [file pcbi.1008751.s020.tif]

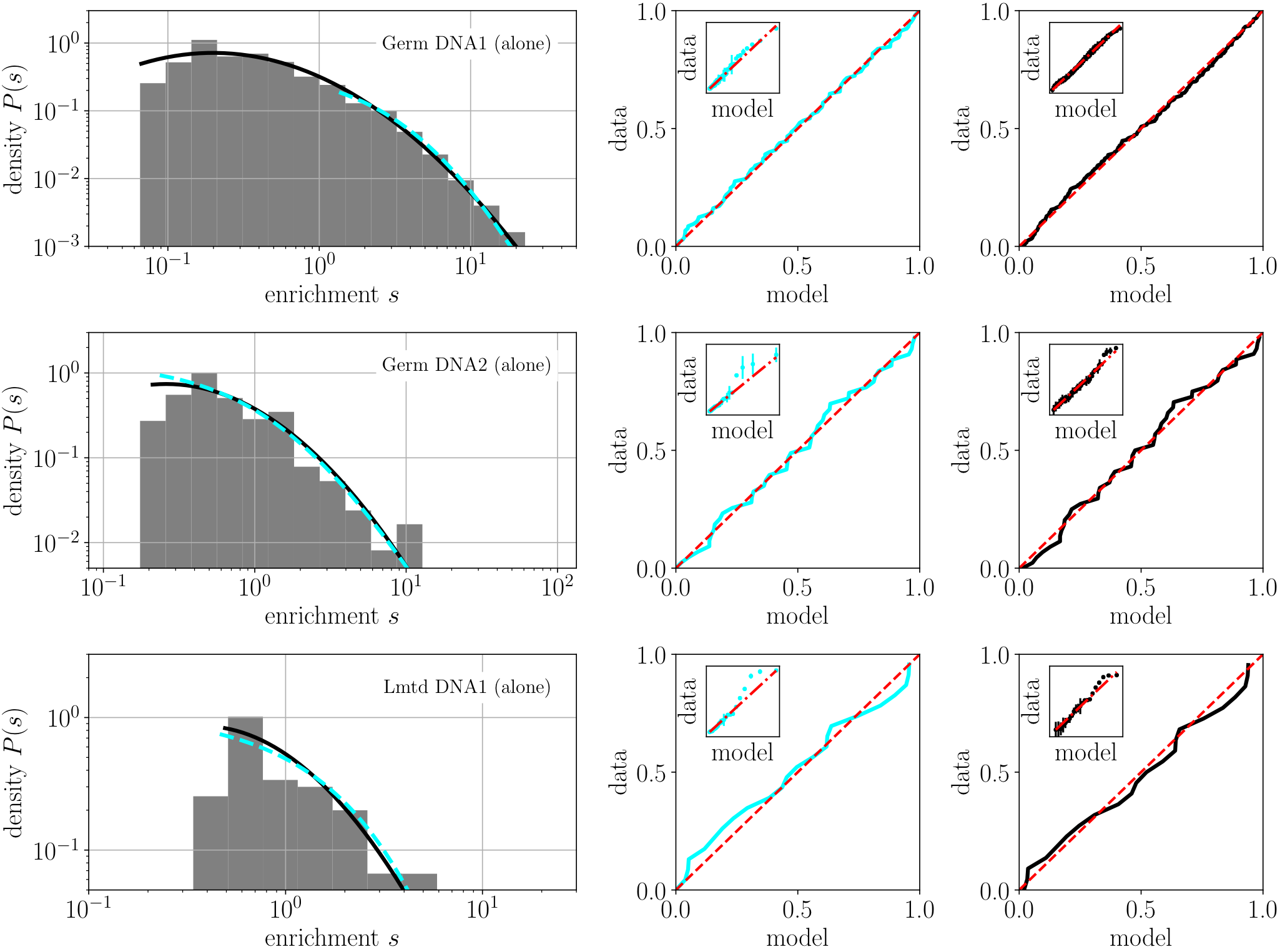

Supplement: S19 Fig — (TIF) [file pcbi.1008751.s021.tif]

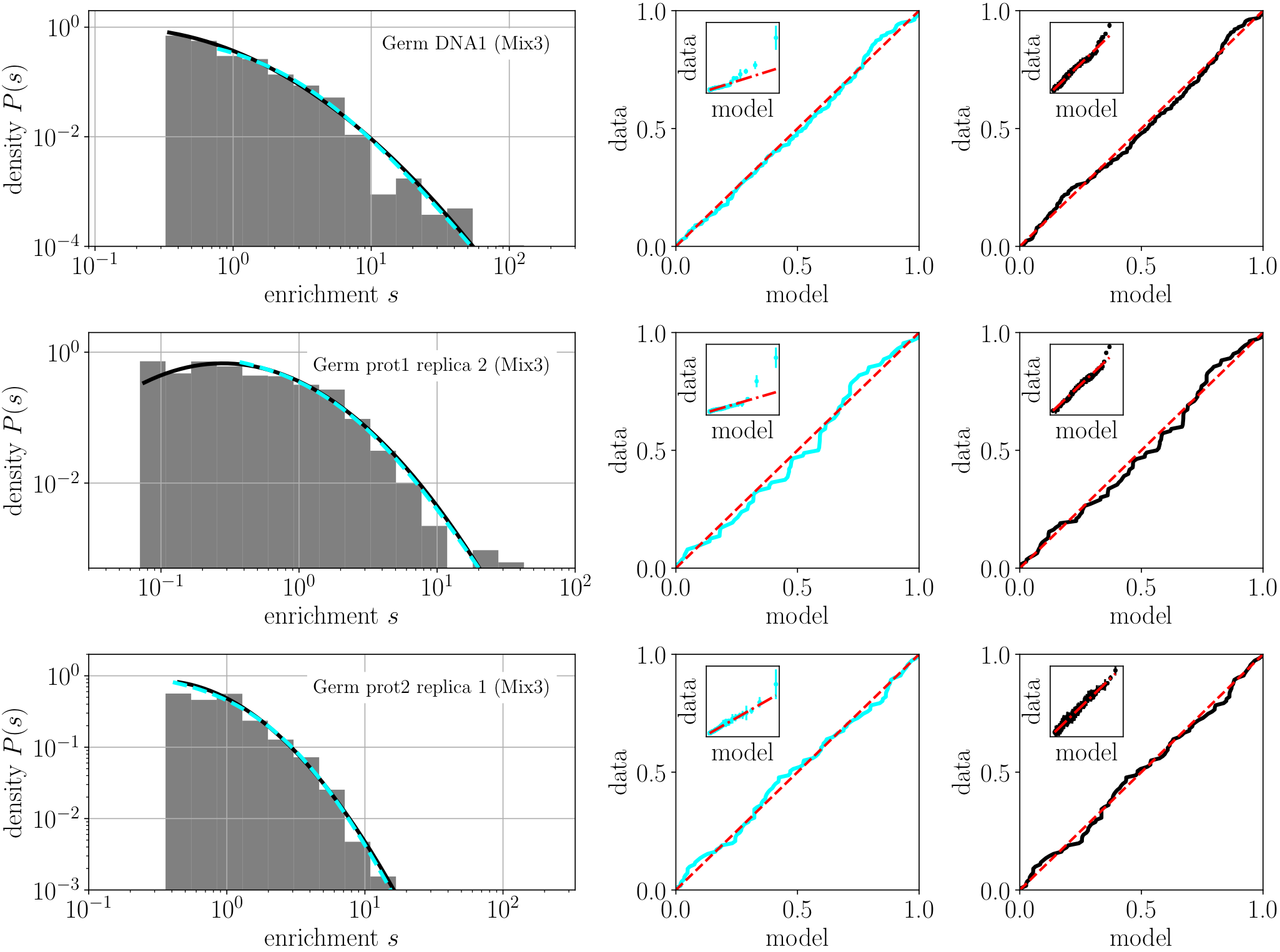

Supplement: S20 Fig — (TIF) [file pcbi.1008751.s022.tif]

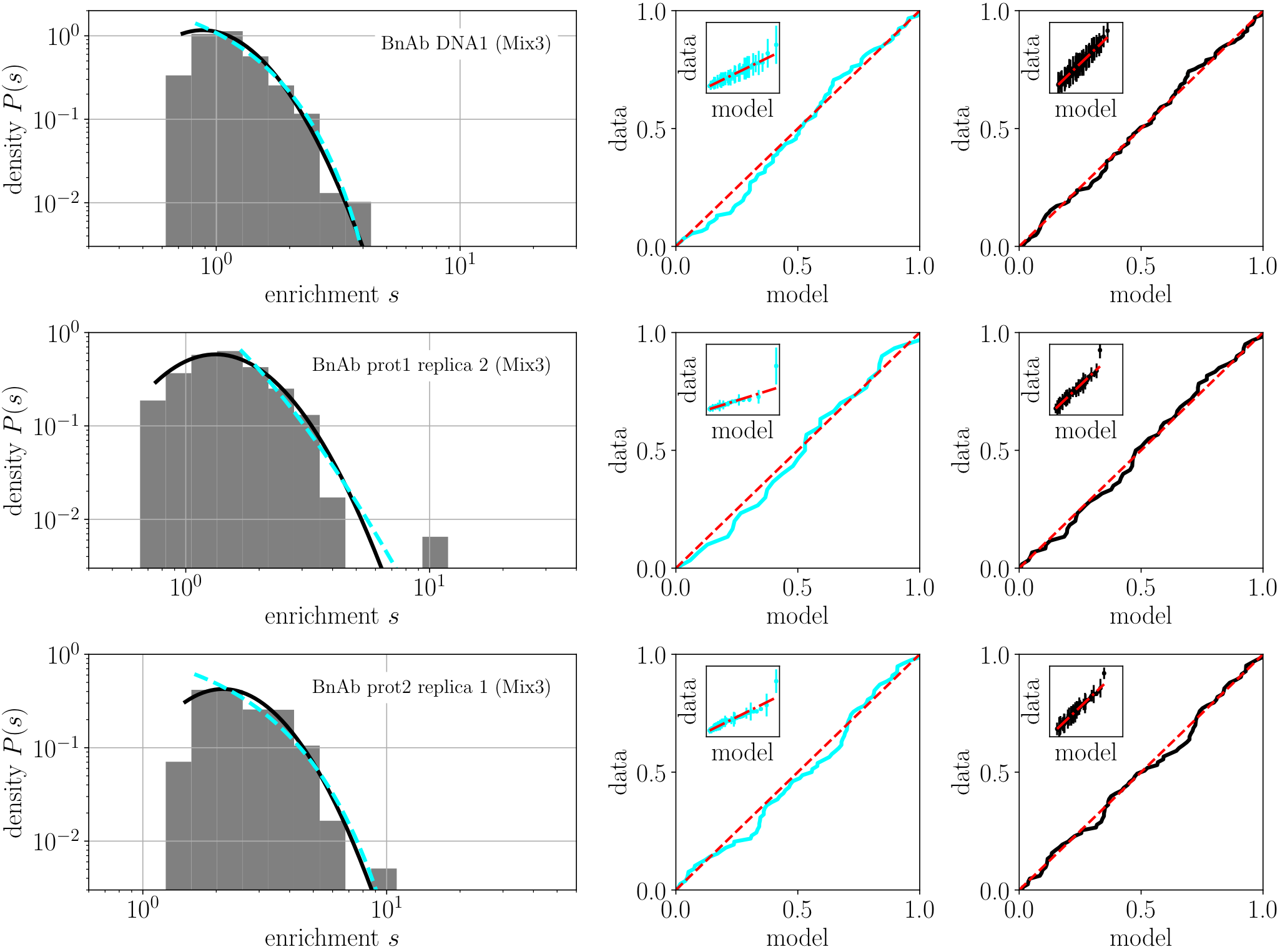

Supplement: S21 Fig — (TIF) [file pcbi.1008751.s023.tif]

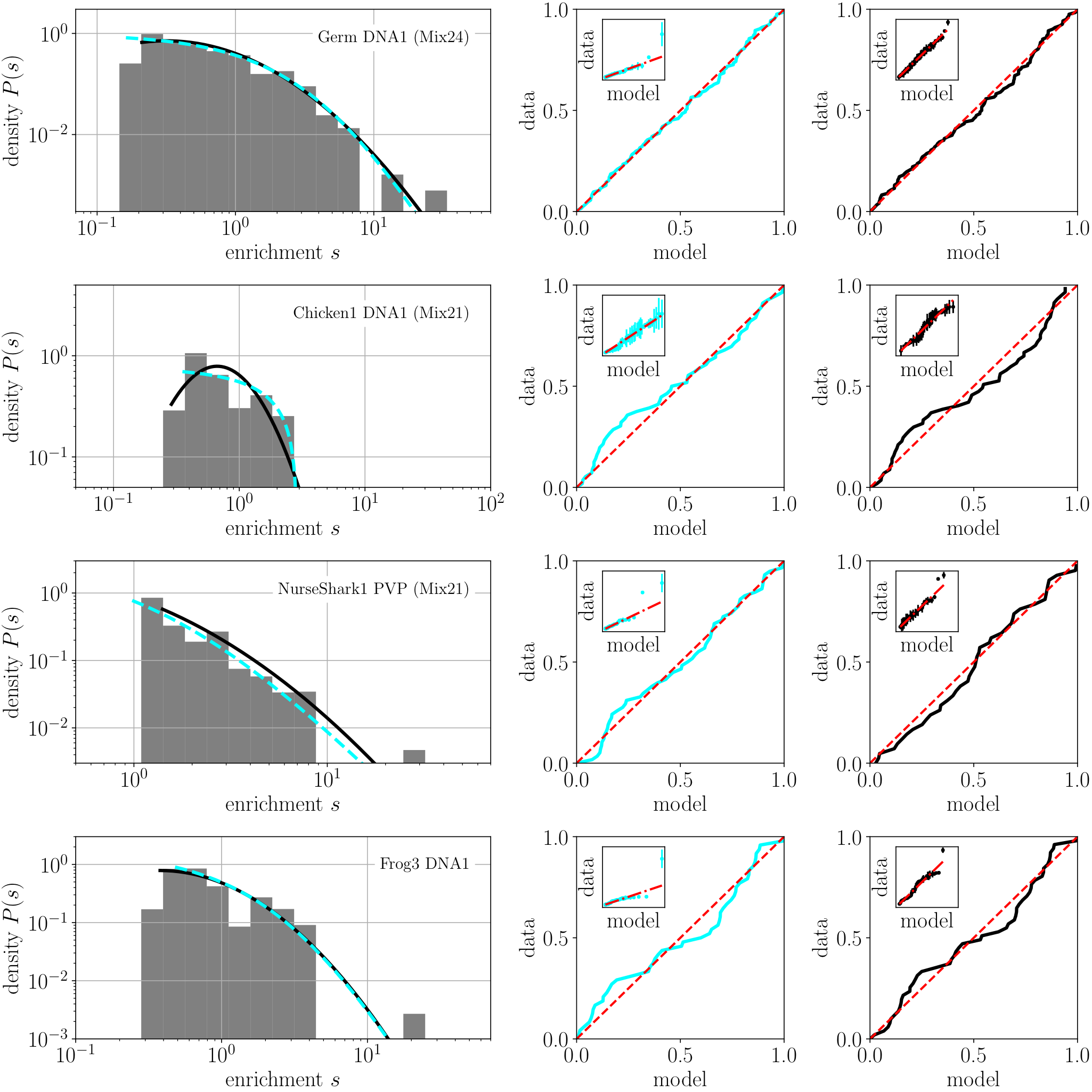

Supplement: S22 Fig — (TIF) [file pcbi.1008751.s024.tif]

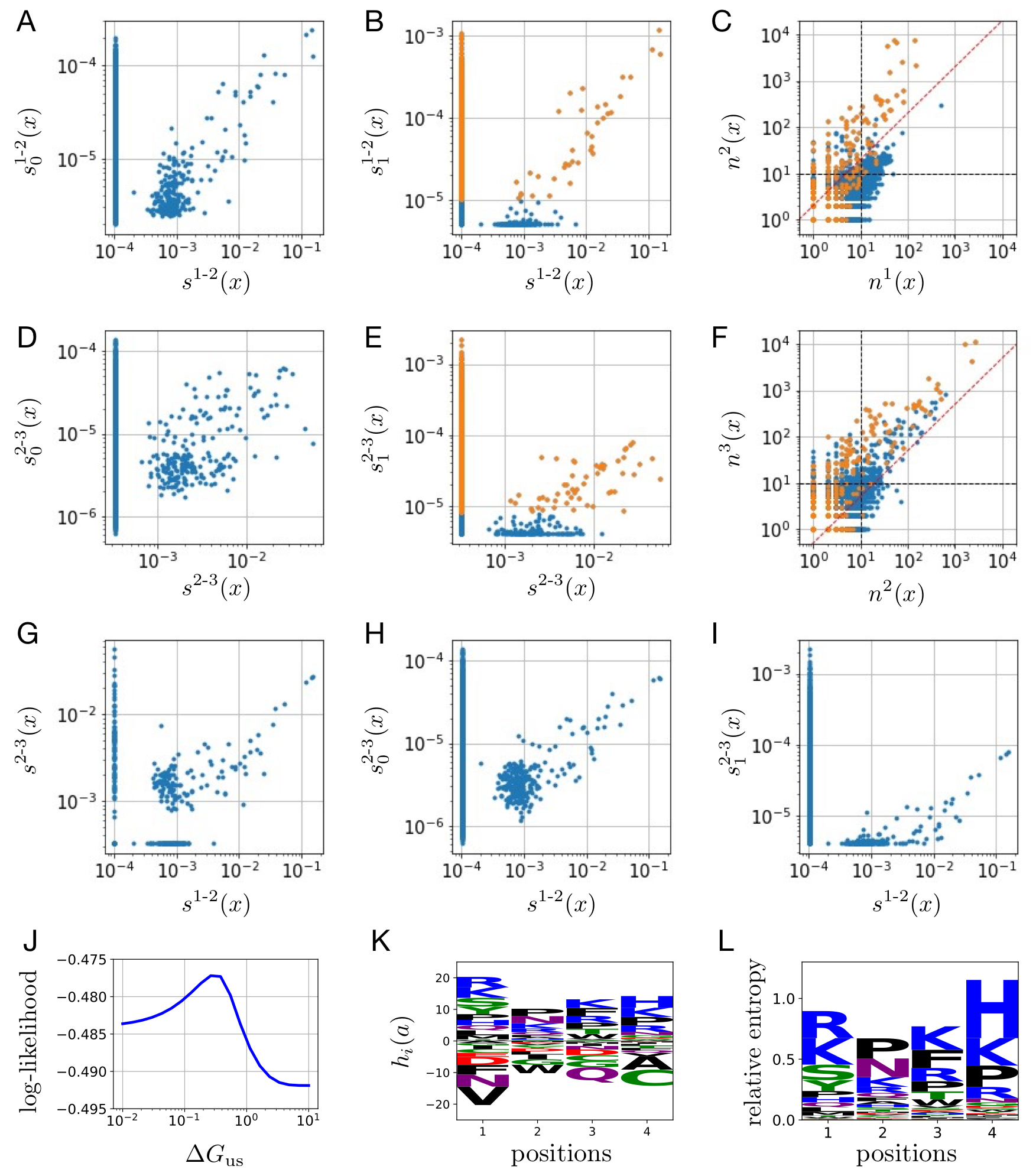

Supplement: S23 Fig — The data consists in the counts n1(x), n2(x), n3(x) at the different rounds (panels C and F), from which enrichments are inferred in different ways that we compare. As in the main text, we define s1- 2(x) ∝ n2(x)/n1(x) when n1(x) ≥ 10 and n2(x) ≥ 10, and s2-3(x) ∝ n3(x)/n2(x) when n2(x) ≥ 10 and n3(x) ≥ 10: they are shown in panel G to give consistent results (undefined values are represented as small values). Alternatively, we can infer enrichments by maximum likelihood using the model in section 1.5 of S1 Text. For each successive rounds c-(c + 1) with c = 1 or 2, two solutions are considered: s0c-(c+1)(x) where unspecific binding is neglected (ΔGus = ∞) and s1c-(c+1)(x) where it is not (ΔGus treated as variable in addition to the hi(a)). They are compared to sc-(c+1) in panels A, B, D, E. In B and E, where unspecific binding is present, the sequences that are predicted to be selected through specific binding (e-βG(x)>e-βGus Eq. 21 of S1 Text) are represented in orange. When considering data between rounds 1- 2, a good agreement is found between s1-2(x) and s11-2(x) (panel B) and the sequences identified as binding specifically (in orange) correspond indeed to those above a threshold, s1-2(x) > s*(panel C). This is not the case when considering the data between rounds 2-3 where the model predicts many sequences with high enrichments s12-3(x) that are not reported in s2-3(x) (panel E). In this case, the solution without non-specific binding s02-3(x) appears to be more relevant. This is confirmed in panels H and I where s1-2(x) is seen to correlate better with s02-3(x) than with s12-3(x). Panel J represents the maximum value of the log-likelihood for fixed values of ΔGus, showing the presence of a non-trivial optimum (data from rounds 1-2). The fields hi(a) of this model are shown in panel K in the zero-sum gauge where ∑a=1qhi(a)=0 for all i. The same information can also be represented in the form a sequence logo (panel L), to be compared to the sequence logo obtai [file pcbi.1008751.s025.tif]

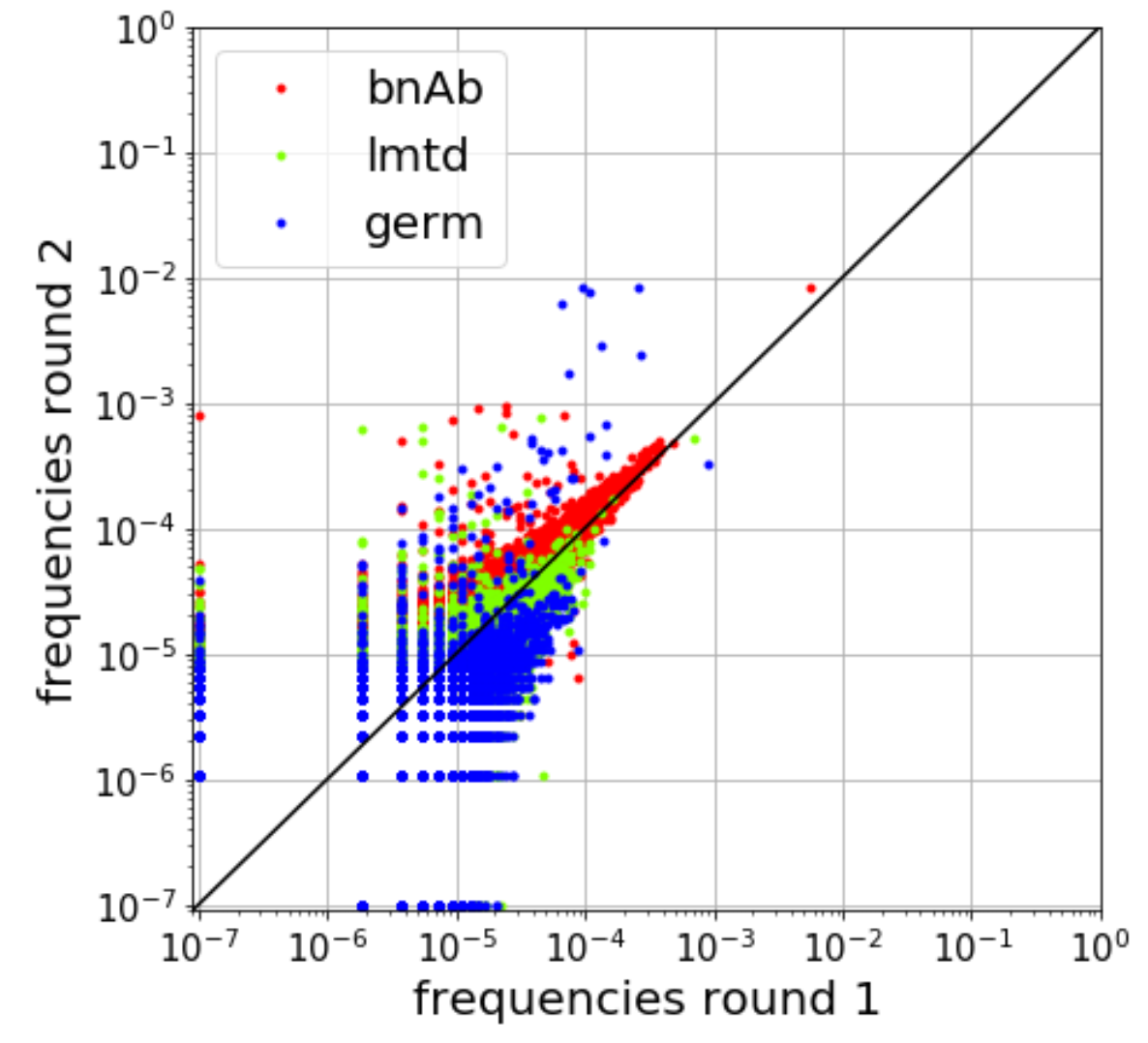

Supplement: S24 Fig — This figure shows that each library has a different background noise. (TIF) [file pcbi.1008751.s026.tif]

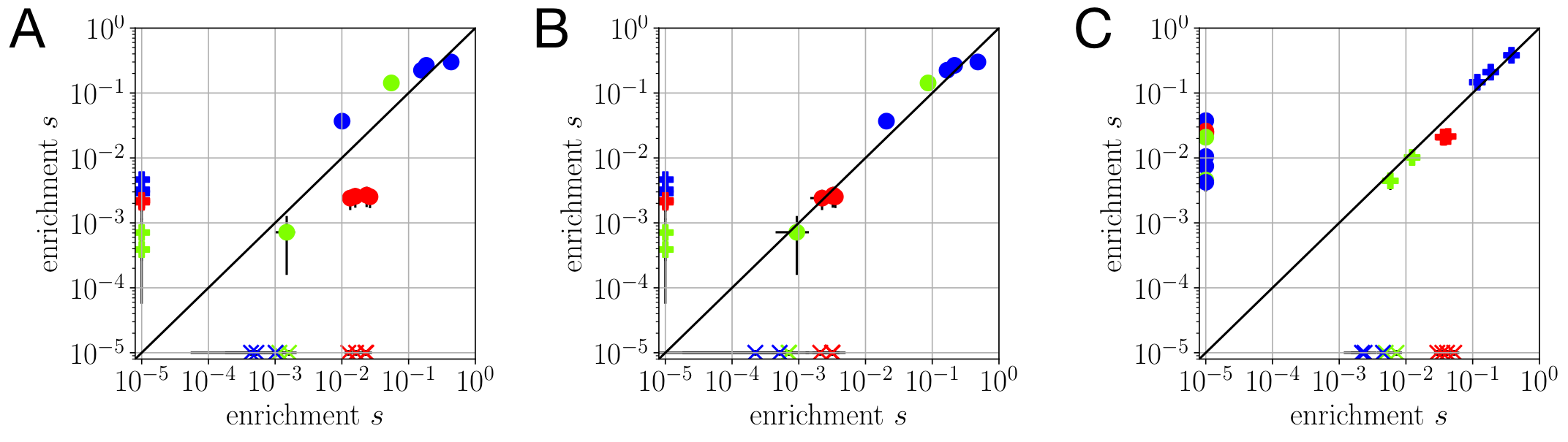

Supplement: S25 Fig — A. Enrichments from S13(A) Fig versus S13(C) Fig: the results from the two experiments are highly reproducible except for the Bnab sequences in red. This difference is due to the different batches of beads used in these two experiments. B. Enrichments from S13(B) Fig versus S13(D) Fig. Here the two experiments use the same batch of beads and the inferred enrichments are all very reproducible. C. Enrichments from S12(B) Fig versus S12(A) Fig, showing again high reproducibility. Error bars are enlarged 20 times to make them visible. (TIF) [file pcbi.1008751.s027.tif]

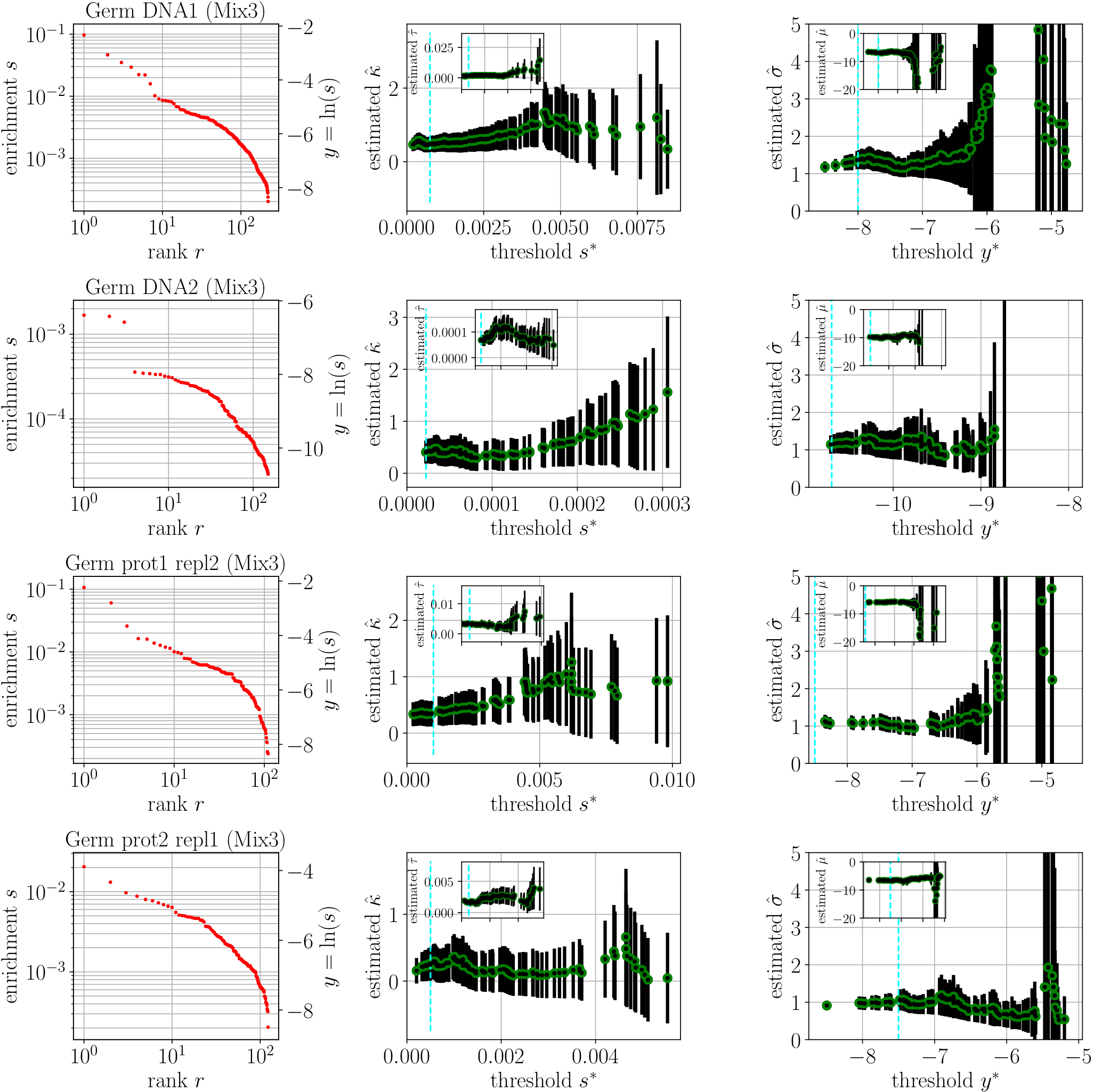

Supplement: S26 Fig — Here for the Germ library selected against different targets. When the threshold is too large, very few data points are left and the error bars, obtained from the Fisher information matrix via the Cramér-Rao bound, are large. In any case, however, the estimation of κ^ and σ^ is consistent across a range of values of the thresholds. (TIF) [file pcbi.1008751.s028.tif]

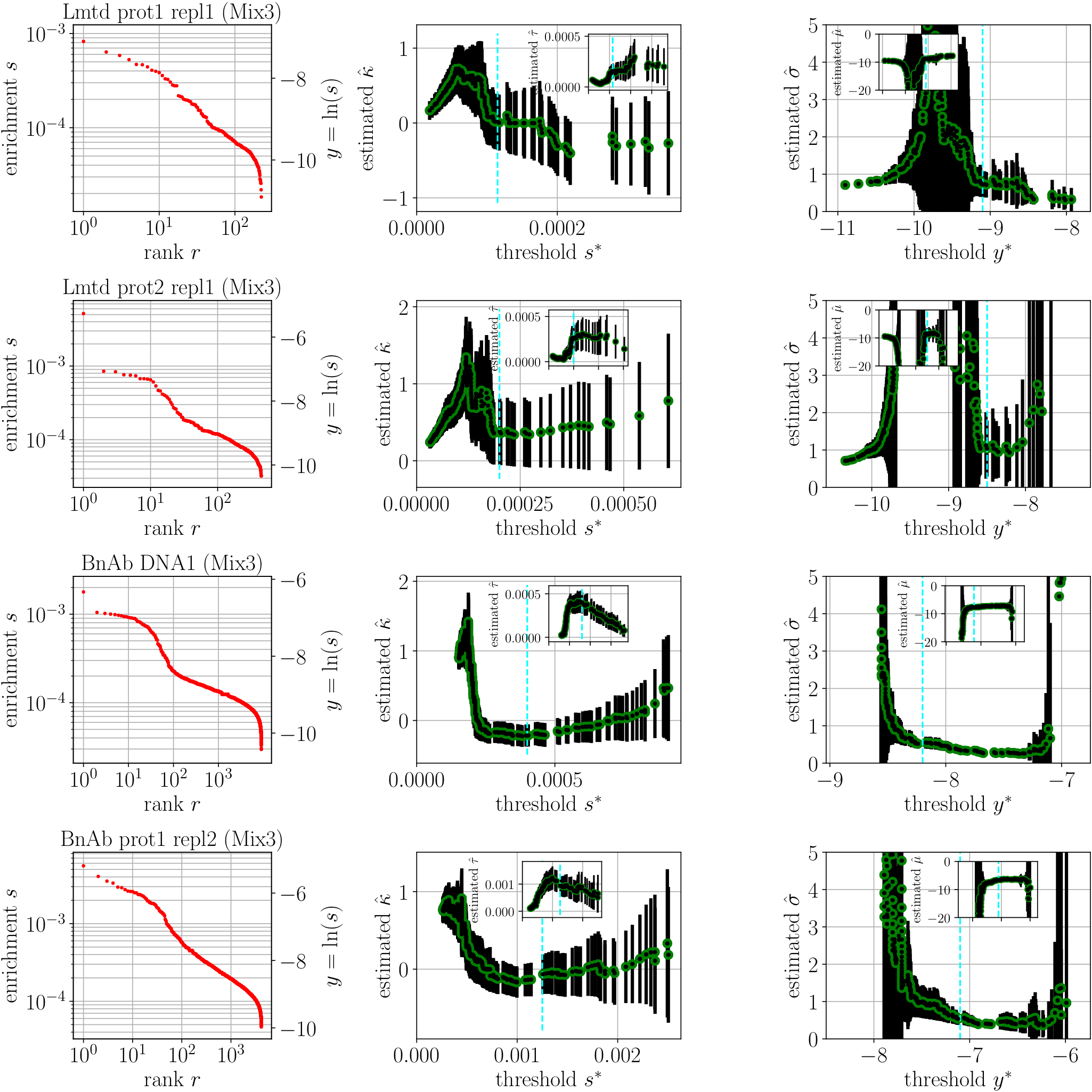

Supplement: S27 Fig — (TIF) [file pcbi.1008751.s029.tif]

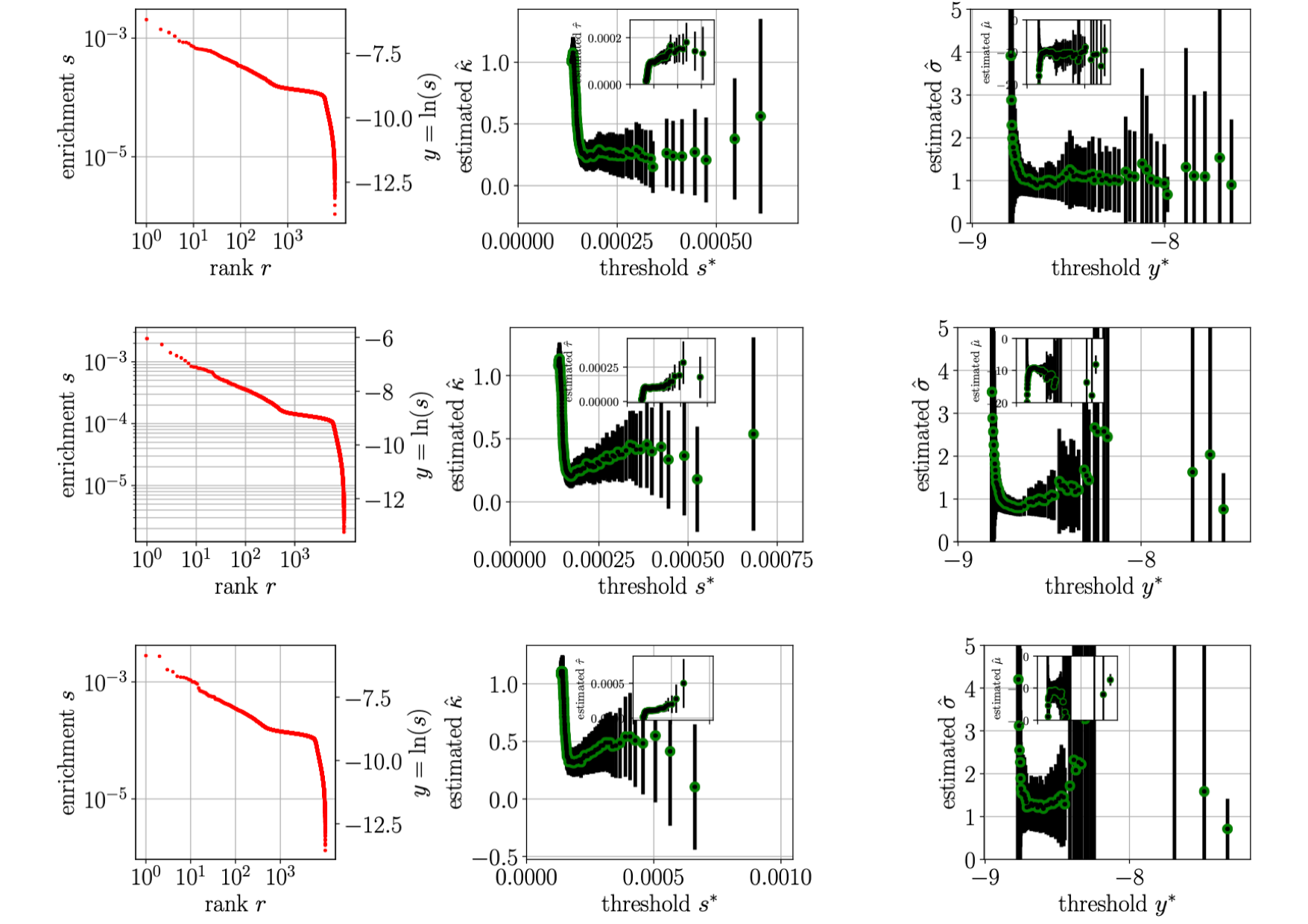

Supplement: S28 Fig — The 3 examples correspond to different draws of N = 104 samples from a mixture model with two equiprobable modes: a bottom (“unspecific”) mode described by a lognormal distribution with parameters μus = −10 and σus = 1 and a top mode described by a lognormal distribution with parameters μ = −9 and σ = 1. The parameters of this top mode are recovered for an intermediate range of thresholds. For too small thresholds, the presence of the bottom mode leads to inconsistent values while for too high thresholds the number of samples becomes insufficient. (TIF) [file pcbi.1008751.s030.tif]
